# New Pinane Derivatives Found in Essential Oils of *Calocedrus decurrens*

**DOI:** 10.3390/molecules22060921

**Published:** 2017-06-02

**Authors:** Gabriel Garcia, Loïc Tissandié, Jean-Jacques Filippi, Félix Tomi

**Affiliations:** 1 Université de Corse-CNRS, UMR 6134 SPE, Équipe Chimie et Biomasse, Route des Sanguinaires, Site de Vignola, 20000 Ajaccio, France; gabrielgarcia@sfr.fr; 2 Institut de Chimie de Nice, Université de Nice-Sophia Antipolis, UMR 7272 CNRS, Parc Valrose, 06108 Nice CEDEX 2, France; loic.tissandie@unice.fr (L.T.); jean-jacques.filippi@unice.fr (J.-J.F.)

**Keywords:** *Calocedrus decurrens*, pin-2-en-8-al, methyl pin-2-en-8-oate, pin-2-en-8-yl Acetate, pin-2-en-8-ol, 2D-NMR, structural elucidation

## Abstract

The main objective of this study was to determine the chemical composition of essential oils (EOs) obtained from leaf, old branches, and young branches of a coniferous species *Calocedrus decurrens* acclimated to Corsica. The analytical investigation was conducted by GC(RI), GC-MS, pc-GC, and NMR. *C. decurrens* leaf, old branches, and young branches EOs contained α-pinene (11.2; 56.6; 22.3%), myrcene (13.4; 8.4; 9.7%), Δ-3-carene (31.3; 5.2; 11.1%), limonene (6.4; 5.1; 5.5%), terpinolene (6.9; 1.5; 3.2%), and pin-2-en-8-ol (4.2; 4.5; 10.4%) as major components, respectively. Special attention was paid to purifying and identifying four unusual pinane derivatives: pin-2-en-8-ol, pin-2-en-8-yl Acetate, pin-2-en-8-al, and methyl pin-2-en-8-oate. The last two are reported for the first time.

## 1. Introduction

Over the last century, the French National Forest Office (“Office National des Forêts”) has introduced several coniferous species in Corsica. These introductions were made for the purposes of testing the growing abilities of these species out of their natural ecosystems. In the course of our studies, several essential oils from these introduced species such as *Pinus halepensis* [1,2], *Larix decidua*, *Pseudotsuga menziesii*, *Pinus ponderosa*, *Sequoiadendron giganteum*, and *Cryptomeria japonica* [3] were investigated. However, another species formerly introduced in Corsica, *Calocedrus decurrens*, was still not studied.

*C. decurrens*, commonly known as incense cedar, is an aromatic conifer belonging to the Cupressaceae family, native to Oregon and California. Its natural area extends to 31° at 46° parallel of north latitude and it is found between 900 and 2500 m altitude. It is widely used in reforestation in the USA because of its rusticity and its adaptation in difficult sites (limestone, shallow, dry land). The bark of *Calocedrus* is thick, reaching 20 cm, and is an excellent protection against forest fires which are very frequent in its native area. Furthermore, *C. decurrens* wood is considered as precious wood, owing to its highly aromatic wood, and is often used for the manufacture of pencils. Its wood is also highly valued for its decorative appearance and durability in carpentry, interior cladding, and furniture [4,5].

In France, this species was introduced around 1850. Since, it has been widespread in many parks and gardens, particularly in the Mediterranean area, for example, Gréoux-les-Bains and Châteauvert [5]. By contrast, in Corsica, the *C. decurrens* species was introduced in 1989 in the forest of Ospedale.

There are already a few articles dealing with the chemical composition of *C. decurrens* essential oils. The first study, conducted in 1954 by Nakatsuka et al., reported the chemical composition of the phenolic fraction from wood EO, which contained as its major components carvacrol and an unknown constituent, most likely either *p*-methoxy-carvacrol or *p*-methoxy-thymol [6]. Wood EO seemed to be mostly constituted by aromatic terpenes. That was corroborated by a heartwood EO studied by Veluthoor et al. also exhibits phenolic terpenes as its major constituents: thymoquinone (35.9%), carvacrol (29.2%), thymol methyl ether (11.0%), and carvacrol methyl ether (3.2%) [4]. In contrast, the leaf EOs were qualitatively different. In 1981, Von Rudloff reported the composition of a leaf EO obtained from a vegetal harvested in Gasquet (Northern California). The main constituents were identified as limonene (31.3%), Δ-3-carene (21.0%), α-pinene (9.2%), myrcene (8.0%), and α-terpenyl acetate (5.7%) [7]. A later study performed in 2006 by Adams et al. reported the chemical composition of leaf EOs of *C. decurrens* from Oregon and California, and described the presence of limonene (18.2–23.6%), Δ-3-carene (15.2–20.2%), α-pinene (8.7–15.8%), myrcene (6.2–8.2%), α-fenchyl acetate (3.5–9.7%), and terpinolene (5.7–8.0%) [8].

In this study, we focus on the analytical investigation of essential oils obtained from leaf, old branches, and young branches of *C. decurrens* acclimated to Corsica. We also give a comparison of their chemical compositions with EOs obtained from trees grown in their native areas. Special attention was paid to the identification of unusual pinane derivatives, such as pin-2-en-8-ol (**A**) and pin-2-en-8-yl Acetate (**B**), and two additional compounds, pin-2-en-8-al (**C**) and methyl pin-2-en-8-oate (**D**), never reported so far in the literature.

## 2. Results

### 2.1. Analysis of C. decurrens Leaf, Old Branches and Young Branches Essential Oils by GC(RI), GC-MS and ^13^C-NMR

For quantitative purposes, essential oils of *C. decurrens* were analyzed by GC(RI). In combination with their retention indices (RI) determined by both polar and apolar stationary phases, volatile constituents were identified by means of GC-MS and ^13^C-NMR, following a computerized methodology developed in our laboratory [9]. The leaf essential oil contained monoterpene hydrocarbons as major constituents such as Δ-3-carene (31.3%), myrcene (13.4%), α-pinene (11.2%), terpinolene (6.9%), and limonene (6.4%), while the EO isolated from old branches was characterized by the preeminence of α-pinene (56.6%), myrcene (8.4%), Δ-3-carene (5.2%), and limonene (5.1%). The young branches oil contained mostly α-pinene (22.3%), along with Δ-3-carene (11.1%), myrcene (9.6%), and limonene (5.5%) (Table 1). Altogether, the three essential oils were qualitatively similar in terms of chemical composition, differing only by their respective content in major monoterpene hydrocarbons. In addition, we noticed the presence of monoterpene esters, among these α-terpinyl acetate (1.0–2.4%) and the uncommon methyl myrtenate (1.6–3.5%). Sesqui- and diterpenes were always present in low amounts.

**Figure 1:**
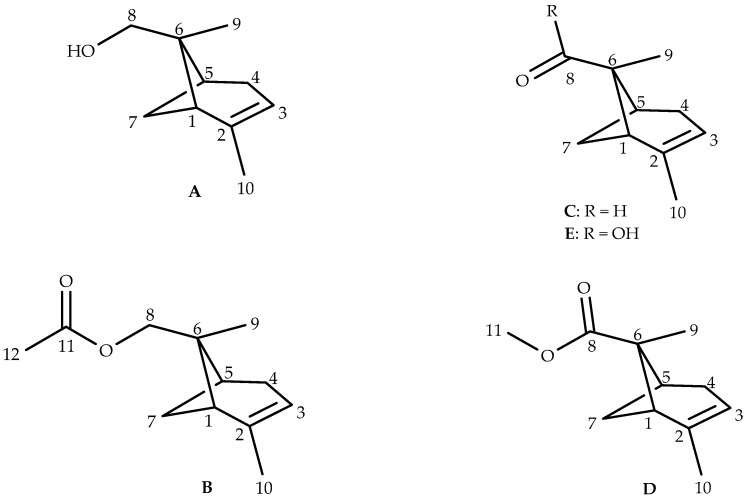
Structure of compounds **A**–**E**.

**Figure 2:**
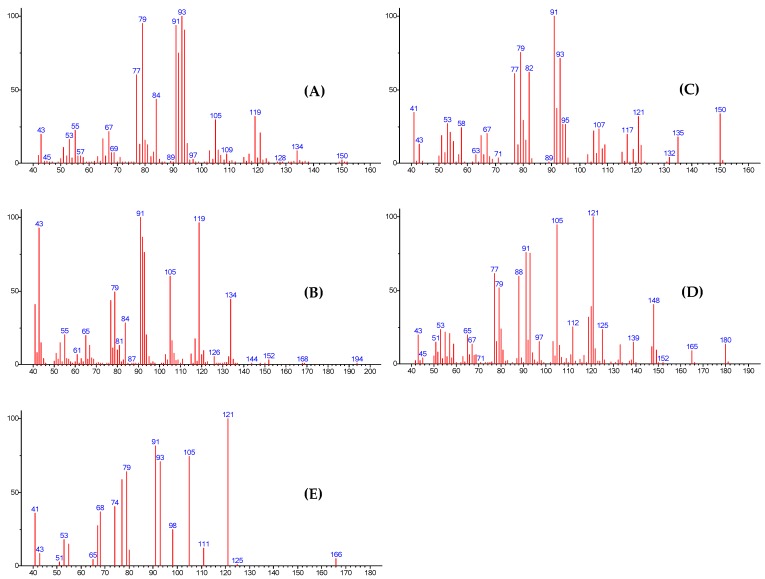
EI-MS spectra of compounds **A**–**E**.

Surprisingly, four unidentified components (probably oxygenated according to their polar retentions indices) monoterpenes **A**–**D** (Figure 1 and Figure 2) were present at appreciable amounts in the leaf (L), old branches (OB), and young branches (YB) essential oils: (i) compound **A** accounting for 4.2% (L), 4.5% (OB), and 10.3% (YB); (ii) **B**: 0.6%, 0.6% and 1.4%; (iii) **C**: 1.6%, 0.8% and 2.0%; and (iv) **D**: 3.0, 1.8, and 3.6%. The structural elucidation of these four compounds is reported below (Figure S1).

### 2.2. Structure Elucidation of Unidentified Compounds

#### 2.2.1. Identification of Pin-2-en-8-ol (**A**)

The first unknown constituent named **A** was identified neither by computer matching against commercial and lab-constructed SM libraries, nor by NMR using our in-house ^13^C-NMR databases. As determined by GC(RI) and GC-MS, the compounds **A**, accounted for 4.2%, 4.5% and 10.3% in the leaf, old branches, and young branches essential oils of *C. decurrens*. RI values measured for this compound, 1189/1800 (RI_a_/RI_p_), suggested a monoterpenic alcohol.

The young branches essential oil containing 10.3% of **A** was subjected to column chromatography (CC) on silica gel using a gradient of solvents (pentane/Et_2_O). Fraction F3 (eluted at 100% Et_2_O), contained 51.8% of the unknown compound. Compound **A** was finally isolated from F3 by means of preparative capillary-gas chromatography (pc-GC), with 95% purity (GC).

The ^13^C-NMR spectrum of the isolated compound displayed 10 signals. Taken altogether, MS and NMR data, especially those provided by the DEPT spectra (2 C, 3 CH, 3 CH_2_ and 2 CH_3_) and by ^1^H and ^13^C-NMR (occurrence of C=CH and methylene alcohol function at 69.00 ppm), suggested the formula C_10_H_16_O.

By taking into account the degree of unsaturation and the multiplicity of carbon signals, compound **A** corresponded to a bicyclic unsaturated monoterpene alcohol. Moreover, from characteristic chemical shifts and signals multiplicity of H-7a/7b (Table 2), we deduced the occurrence of bicyclic unsaturated monoterpene alcohol having a pinane skeleton.

The CH_2_ at 69.00 ppm indicated a primary alcohol, potentially located on carbons 8, 9 or 10. The possibility of a hydroxy group on C-10 was immediately discarded because the ^13^C-NMR values of adjacent carbons 2 and 10 remained close to those of α-pinene [10] (Table 2). The lack of HMBC correlations between hydrogens at 3.50–3.54 ppm (H-8b/8a) and C-2 confirmed the previous observation.

The ^13^C-NMR data of pin-2-en-9-ol are available in the literature [11], and did not match with that of compound **A** (Table 2). Compound **A** was thus identified as pin-2-en-8-ol (**A**). In the case of α-pinene, methyl-8 and methyl-9 strongly differ in their respective ^13^C-NMR chemical shifts, due to the presence of a γ-steric effect between C-9 and C-2 [10]. The structure was further corroborated by examination of the NOESY spectrum, (i) showing no correlation between hydrogens at 3.50–3.54 ppm (H-8b/8a) and the olefinic proton H-3; and (ii) by the presence of a clear correlation between methyl-9 (0.95 ppm) and H-3. The total assignment of compound **A** was finally achieved by the detailed examination of 2D-NMR data (Table 2). We noticed that the compound **A** was partially described over thirty years ago by De Pascual Teresa et al. [12], with a partial description of ^1^H-NMR chemical shifts. In the course of this investigation, we reported for the first time the ^13^C-NMR values, along with an assignment of ^1^H and ^13^C chemical shifts.

#### 2.2.2. Identification of Pin-2-en-8-yl Acetate (**B**)

Compound **B** is the second unknown component of SM libraries and ^13^C-NMR databases, which was present at 0.6, 0.6, and 1.4% in leaf, old branches, and young branches essential oils, respectively. Retention indices for **B** (RIa/RIp = 1310/1683) suggested an ester. The presence of common odd-electron ions (OE^•+^: 84, 92, 134 *m*/*z*) in both mass spectra of **A** and **B** indicated a possible structural relationship between the two compounds. Moreover, the presence of OE^•+^ at *m*/*z* = 134 potentially indicated a [M^•+^ − AcOH] loss from the molecular ion observed at *m/z* = 194 (Figure 2). Compound **B** was finally isolated from a fraction of the young branches essential oil. Fraction F2 (eluted with 95/5 pentane/Et_2_O) contained 8.0% of **B**, which was subsequently isolated by pc-GC with 76% purity (GC).

The ^13^C-NMR spectrum of **B** displayed twelve signals. Multiple DEPT experiments allowed for differentiating of three quaternary carbons, three CH, three CH_2_, and three CH_3_. Moreover, the quaternary carbon at 170.48 ppm and the methylene at 70.58 ppm confirmed the presence of an ester. The presence of a singlet at 1.72 ppm in the ^1^H spectrum correlated to a methyl signal at 20.53 ppm in the HSQC spectrum suggested an acetate group. This was confirmed by the correlation between H-12 and C-11 in the HMBC spectrum. Altogether, MS and NMR data permitted to envisage a pinane ester having the formula C_12_H_18_O_2_. By comparison of its ^13^C-NMR data with that of pin-2-en-8-ol (**A**), we noted that only C-8 and C-6 were impacted by the presence of the acetate. C-8 moved upfield by 1.58 ppm, and C6 moved downfield by 1.76 ppm. Such variations of chemical shifts are commonly encountered when alcohols are compared to their corresponding acetates [13].

As final proof, the examination of the NOESY spectrum allowed us to notice correlations between hydrogen at 0.96 ppm (H-9) with H-3, and the absence of a correlation between hydrogens at 4.26–4.32 ppm (H-8b/8a) and H-3, thus confirming the orientation of the acetate in exo-position to the main cycle. All these data permitted the identification of compound **B** as pin-2-en-8-yl Acetate (Table 3). As with compound **A**, **B** was partially described by De Pascual Teresa et al. [12], with some ^1^H-NMR chemical shifts and MS data. Herein, we detail a full set NMR data, among which includes: (i) a complete ^1^H-NMR spectrum; (ii) ^13^C-NMR values (described for the first time); and (iii) full 2D-NMR data. Interestingly, Adams et al. reported in 2006 the chemical composition of a leaf essential oil from *C. decurrens* [8]. In their study, the authors mentioned an unidentified component at RI = 1330 (DB-5). Although their RI value is shifted 20 points compared to our value, owing to the difference of stationary phase, the MS fragmentation previously reported is very similar to that we observed for **B**.

#### 2.2.3. Identification of Pin-2-en-8-al (**C**)

A third compound (**C**), having RI values at 1102/1460 (RIa/RIp), could not be identified by GC–MS and ^13^C-NMR analysis, even by using all of the computerized commercial MS libraries at our disposal, as well as our laboratory-built ^13^C-NMR data library—it was unknown from our MS and ^13^C-NMR databases. This component was present in leaf, old branches, and young branches EO of *C. decurrens* in 1.6%, 0.8%, and 2.0%, respectively. The examination of the MS spectrum showed a M^•+^ at *m*/*z* = 150, suggesting the possible presence of an aldehyde function. However, despite our many tests, any attempt of purification by pc-GC failed, since the isolated product was never pure, prompting us to adopt another strategy.

Thus, the pyridinium chlorochromate (PCC) oxidation of pin-2-en-8-ol (**A**) available in F3 at 51.8% yielded an aldehyde (45.6% in oxidized F3 named F3’), perfectly corresponding with both the RI values and MS data observed for compound **C**. The compound was further purified by means of column chromatography (CC) on silica gel using the following gradient of solvents pentane/Et2O 95/5 to yielded F3’-1 (11.6 mg) containing 80% of pin-2-en-8-al (**C**). However, we noticed a rapid oxidation of this aldehyde between GC (directly recorded after CC) and NMR/GC-MS analysis (recording after 2 h). The compound **C** seemed to oxidize under atmospheric conditions—indeed, GC-MS and NMR analysis indicated the formation of corresponding acid: pin-2-en-8-oic acid (**E**) (Figure 2 and Table 4).

The ^13^C-NMR spectrum displayed ten signals with strong intensity corresponding at the compound **C**. Subsequent DEPT experiments allowed us to determine the multiplicity of the different carbon signals, and suggested from NMR values, once more, a component having the pinane skeleton (C_10_H_14_O). The methine (CH) observed at 204.19 ppm confirmed the presence of the aldehyde. Additionally, the ^13^C chemical shifts of the two olefinic carbons were similar to those of pin-2-en-8-ol (**A**), and a comparison with the chemical shifts reported for pin-2-en-9-al [11] confirmed we had isolated its epimer. Finally, after examination of all 2D-NMR data, compound **C** was unambiguously identified as pin-2-en-8 al (**C**) (Table 4).

Herein again, pin-2-en-8-al (**C**) might correspond to an unknown compound reported by Adams et al. in 2006 [8]. Both the RI value (1120) and MS data published by the authors are in fair concordance with our results, thus suggesting it is probably the same molecule.

#### 2.2.4. Identification of Methyl Pin-2-en-8-oate (**D**)

A fourth compound (**D**) respectively present in 3.0%, 1.8% and 3.6% in leaf, old branches, and young branches oils remained unknown from our MS and ^13^C-NMR database. Its RI values, 1207/1543 (RIa/RIp), suggested an oxygenated monoterpene. Fraction F2 (eluted at 95/5 pentane/Et_2_O) obtained from the young branches EO contained 17.8% of compound **D**. This compound was isolated from F2 by means of pc-GC, giving **D** in 91% purity (GC).

The MS spectrum indicated an M^•+^ at *m*/*z* = 180. The ^13^C-NMR spectrum exhibited eleven signals having the following multiplicity: three quaternary carbons, three CH, two CH_2_, and three CH_3_. The two chemical shifts at 51.47 ppm (CH_3_-O) and 178.28 ppm indicated the presence of a methyl ester. Here again, the degree of unsaturation calculated from the formula C_11_H_16_O_2_ suggested a pinane derivative. Interestingly, by comparison of chemical shifts of the compound **D** with those of pin-2-en-8-ol (**A**), we noticed that only C-6, C-8, and C-11 were impacted by the presence of the ester. The comparison with ^13^C-NMR values of pin-2-en-8-oic acid (**E**) was even more revealing—indeed, all chemical shifts are very similar excepted: (i) C-8, which was moved downfield by 6.63 ppm (indicating an ester function) and (ii) C-11, which was not present in (**E**) and being characteristic of methoxy group. As described above for others compounds, the analysis of the NOESY spectrum indicated that the ester function is positioned on C-8 (Table 5). According to the HMBC spectrum, the signal at 51.47 ppm, characteristic of a methoxy group, was correlated with C-8, thus confirming the presence of a methyl ester.

## 3. Discussion

The combined use of GC-MS and ^13^C-NMR for the analysis essential oils obtained from leaf, old branches, and young branches of *Calocedrus decurrens* led to the identification of 45, 68 and 77 components. These accounted for 93.8%, 99.8% and 89.9% of the respective essential oil compositions. The major monoterpenic constituents identified in leaf, old branches, and young branches EOs were α-pinene (11.2; 56.6; 22.3%), myrcene (13.4; 8.4; 9.6%), Δ-3-carene (31.3; 5.2; 11.1%), limonene (6.4; 5.1; 5.5%), and terpinolene (6.9; 1.5; 3.2%). Other components present at appreciable amounts were pin-2-en-8-ol (**A**) (up to 10.3% in young branches oil); and pin-2-en-8-yl Acetate (**B**) (up to 1.4% in young branches oil), whose presence was previously reported in *Aristolochia longa* EO [12]. Hence, the ^13^C-NMR data for this both compounds are reported here for the first time. Additionally, two new compounds, pin-2-en-8-al (**C**) (up to 2.0% in young branches oil) and methyl pin-2-en-8-oate (**D**) (up to 3.6% in young branches oil) were isolated and fully characterized. Furthermore, we reported the ^13^C-NMR values of pin-2-en-8-oic acid (**E**), which is the oxidation product of pin-2-en-8-al (**C**).

The essential oils obtained from the different parts of *Calocedrus decurrens* from Corsica are qualitatively close in terms of chemical composition, but they present significant differences related to their content of each major monoterpenes. Taken altogether, these EOs are qualitatively close to that described by Adams et al. and Von Rudloff [7,8]. Even if it was difficult to make any further comparison since the old and young branches were studied for the first time, we can nevertheless note the total absence of phenolic compounds in our samples.

From an ecological point of view, the *C. decurrens* species seems to be adaptable to the hard conditions in Corsica, and could be a good alternative for reforestation in some areas, due to its strong resistance against the forest fire.

## 4. Materials and Methods

### 4.1. Plant Material, Isolation of Essential Oils

The experimental Ospedale forest (southeast of the Corsica) located at 960 m altitude (GPS coordinates: 41°39,578′ N, 009°11,413′ E) was created by the French National Forest Office (“Office National des Forêts”) in 1989 [14]. Thus, these trees are about thirty years old.

We harvested leaf, old branches (parts of branches previously growing), and young branches (parts of branches actually growing), that we will sometimes refer to as, respectively: L; OB, and YB.

The essential oil samples (L EO = 4.8 g; OB EO = 298.1 mg; YB EO = 307.4 mg) were obtained by water distillation (during 3 hours) using a Clevenger-type apparatus from leaf (997.4 g), old branches (160.5 g), and young branches (78.5 g) of *C. decurrens*.

A voucher specimen was deposited at the Conservatoire Botanique National de Corse, (Corte, France), under accession numbers GG2776.

### 4.2. Fractionation of the Young Branches Essential Oil

The essential oil obtained from the young branches of *Calocedrus decurrens* (296.0 mg) was separated into three fractions F1-3 by column chromatography (CC) on silica gel (60–200 μm, 60 Å, 23.2 g) using the following gradient of solvents: pentane/Et_2_O 100/0 (F1 = 80.2 mg), 95/5 (F2 = 45.7 mg), and 0/100 (F3 = 76.2 mg).

Fraction F2 contained compounds **B** (8.0%), **C** (7.2%), and **D** (17.8%); Fraction F3 contained compound **A** (51.8%). F2 and F3 were directly used in preparative capillary-gas chromatography for the isolation of the target compounds **A**, **B**, **C** (attempts failed), and **D**. Sub-fractions F2-1 contained **B** (76%), F2-2 contained **D** (91%), and F3-1 contained **A** (95%). The three sub-fractions were subjected to spectroscopic analysis for structure elucidation.

### 4.3. Preparation of pin-2-en-8-al *(**C**)*

In a 50 mL round bottom flask equipped with a reflux condenser, 225.6 mg (1.05 mmol) of pyridinium chlorochromate (PCC) and 31.1 mg (0.23 mmol) of sodium acetate were suspended in 2 mL of anhydrous CH_2_Cl_2_. Fraction F3 (76.2 mg) containing 51.8% of **A** (39.5 mg, 0.26 mmol of **A**) was diluted in 3 mL of CH_2_Cl_2_ and added to the PCC solution under stirring in one portion. After 7 h, 20 mL of dry Et_2_O was added and the supernatant decanted from the black gum. The insoluble residue was washed thoroughly 3 times with 10 mL portions of anhydrous Et_2_O whereupon it became a black granular solid. The combined organic layers were passed through a short pad of Florisil, and the solvent was evaporated to yield fraction F3’ (42.1 mg) containing 45.6% of compound **C**. The compound was further purified by means of column chromatography on silica gel (60–200 μm, 60 Å, 30 g) using the following gradient of solvents: pentane/Et_2_O 95/5 to yielded F3’-1 (11.6 mg) containing 80% of pin-2-en-8-al (**C**).

### 4.4. Gas Chromatography

Analyses were performed on a Clarus 500 PerkinElmer (PerkinElmer, Courtaboeuf, France) system equipped with a FID and two fused-silica capillary columns (50 m × 0.22 mm, film thickness 0.25 µm), DB-1 (polydimethylsiloxane) and DB-WAX (polyethylene glycol). The oven temperature was programmed from 60 °C to 220 °C at 2 °C/min and then held isothermal at 220 °C for 20 min; injector temperature: 250 °C; temperature detector: 250 °C; carrier gas: helium (0.8 mL/min); split: 1/60; injected volume: 0.5 µL. Retention indices (RI) were determined against a series of n-alkanes with linear interpolation (Target Compounds software from PerkinElmer). The quantification method is according to Tissot et al. [15], (i) methyl octanoate was used as an internal reference; (ii) relative response factors (RRF) were calculated for each compound; and (iii) the relative proportions of each constituent were deduced using the formula detailed in the article previously cited and expressed in g/100 g.

### 4.5. Gas Chromatography-Mass Spectrometry in Electron Impact Mode

Essential oils samples were analyzed with a Perkin-Elmer TurboMass detector quadrupole (Perkin-Elmer, Courtaboeuf, France), directly coupled to a Perkin-Elmer Autosystem XL, equipped with a fused-silica capillary column (50 m × 0.22 mm i.d., film thickness 0.25 µm), DB-1 (polydimethylsiloxane). Carrier gas, helium at 0.8 mL/min; split: 1/74; injection volume: 0.5 µL; injector temperature: 250 °C; oven temperature programmed from 60 °C to 220 °C at 2 °C/min and then held isothermal at 220 °C for 20 min. Ion source temperature: 250 °C; energy ionisation: 70 eV; electron ionisation mass spectra were acquired over the mass range 35–350 Da.

### 4.6. Preparative Capillary-Gas Chromatography

Isolation of compounds **A**, **B** and **D** were performed using an Agilent 6890 Plus gas chromatograph coupled to a Gerstel preparative fraction collector (PFC) (Agilent, Santa Clara, CA, USA), operated under Chemstation Rev A.10.02/Gerstel Maestro 1.3.8.14. The GC was equipped with a Phenomenex ZB-5 megabore capillary column (30 m × 0.53 mm; 3.0 µm film thick). A Graphpack effluent splitter was connected to the column outlet, and additionally mounted with 0.1 mm and 0.32 mm deactivated fused-silica capillary restrictors (1 m each), to provide an FID/PFC ratio of ~1/9. The transfer line and the PFC were maintained at 230 °C. The injected volume was 1 μL in splitless mode. The oven temperature was increased from 70 to 120 °C at 10 °C/min, then from 120 °C to 250 °C at 20 °C/min. The system was operated in constant pressure mode at 35 kPa. Compounds were trapped at 5–10 °C in Gerstel U-type glass tubes by programming cutting times into the operating software, allowing for accurate automated operation. The isolation of any unknown compound in amounts sufficient for NMR analysis required 150–400 GC runs, and to avoid all contamination, each product was collected directly in a NMR tube.

### 4.7. Gas Chromatography-High Resolution Mass Spectrometry

High-resolution EI-mass spectra were recorded using an Agilent 7200 GC-QTOF system (Agilent, Santa Clara, CA, USA), equipped with a Agilent J&W, VF-waxMS capillary column (30 m × 0.25 mm; 0.25 μm film thick). The mass spectrometer was operated at 70 eV with an acquisition rate of 2 GHz over a 35−450 *m*/*z* range, affording a resolution of ~8000. Injection volume 1 μL; split ratio 1/20; inlet temperature 250 °C, detector temperature 230 °C; column flow (He) 1.2 mL/min; temperature program for oven 60 °C (5 min isotherm) to 240 °C at 5 °C/min (10 min final isotherm).

### 4.8. Nuclear Magnetic Resonance

NMR spectra for compounds **A**, **B**, **C**, **D** and **E** were recorded in C_6_D_6_ at 298 K on a Bruker Avance DRX 500 spectrometer (Bruker, Wissembourg, France) operating at 500.13 MHz for ^1^H, and 125.75 MHz for ^13^C. In order to increase sensitivity, ^13^C-NMR spectra, such as broadband-^13^C, DEPT 135, and DEPT 90, were run with a direct probe head (5 mm PADUL ^13^C-^1^H Z-GRD). 1D- and 2D-NMR spectra such as ^1^H, COSY, NOESY, HSQC, HMBC were run with an inverse probe head (5 mm PHTXI ^1^H-^13^C/^15^N Z-GRD). Spectrum calibration was performed by using the C_6_D_6_ signal as internal reference (7.16 ppm for ^1^H-NMR, 128.06 ppm for ^13^C-NMR). Chemical shifts (δ) are expressed in parts per million (ppm) and coupling constants (*J*) in hertz. All NMR experiments were carried out using pulse sequences supplied by the spectrometer manufacturer (Bruker Topspin^TM^, Bruker, Wissembourg, France) and processed via Mestrelab MestreNOVA software (Version 6.0.2-5475).

Other spectra were recorded on a Bruker AVANCE 400 (100.623 MHz for ^13^C) (Bruker, Wissembourg, France) equipped with a 5-mm probe, in deuterated chloroform (CDCl_3_), with all shifts referred to 7.26 ppm for ^1^H and 77.16 ppm for ^13^C. The ^1^H-NMR spectra were recorded with the following parameters: pulse width (PW) 4.3 ms; acquisition time 2.6 s for 32 K data table with a spectral width (SW) of 6000 Hz (15 ppm). ^13^C-NMR spectra were recorded with the following parameters: PW 4 μs (flip angle 45°); acquisition time 2.7 s for 128 K data table with a spectral width of 24,000 Hz (240 ppm); total repetition time 2.8 s; CPD (composite pulse decoupling) mode decoupling; digital resolution 0.183 Hz/point.

### 4.9. Identification and Quantification of Individual Components

Identification of individual components was based: (a) on a comparison of their GC retention indice (RI) values on both polar and apolar stationary phases, with the literature [16]; (b) on computer searches using digital libraries of mass spectral data and comparison with published data [16,17,18]; (c) on a comparison of the signals in the ^13^C-NMR spectra of the mixtures with those of reference spectra compiled in the laboratory spectral library, with the help of laboratory-made software [9].

### 4.10. Spectral Data

*Pin-2-en-8-ol* (**A**): C_10_H_16_O; HREIMS: *m*/*z* 152.1194 (calcd. for C_10_H_16_O, 152.1207); EI-MS 70 eV, *m*/*z* (rel. int.): 152 (1, M^•+^), 150 (2), 134 (8, M^•+^ − H_2_O), 121 (20), 119 (30), 105 (29), 94 (90), 93 (100), 92 (75), 91 (93), 84 (43), 79 (95), 77 (60), 67 (21), 65 (16), 55 (22), 53 (16), 43 (19). ^1^H-NMR (C_6_D_6_, 500 MHz) and ^13^C-NMR (C_6_D_6_, 125 MHz): see Table 2.

*Pin-2-en-8-yl Acetate* (**B**): C_12_H_18_O_2_; HREIMS: *m*/*z* 134.1086 ([M^•+^ − AcOH]) (calcd. for C_10_H_14_, 134.1101); EI-MS 70 eV, *m*/*z* (rel. int.): 194 (1, M^•+^), 152 (3), 134 (44, M^•+^ − AcOH), 119 (96), 105 (60), 93 (76), 92 (86), 91 (100), 84 (28), 79 ( 49), 77 (43), 65 (19), 55 (20), 43 (93), 41 (41). ^1^H-NMR (C_6_D_6_, 500 MHz) and ^13^C-NMR (C_6_D_6_, 125 MHz): see Table 3.

*Pin-2-en-8-al* (**C**): C_10_H_14_O; HREIMS: *m*/*z* 150.1034 (calcd. for C_10_H_14_O, 150.1050); EI-MS 70 eV, *m*/*z* (rel. int.): 150 (33, M^•+^), 135 (18), 121 (31, M^•+^ − CHO), 117 (20), 107 (23), 105 (22), 95 (26), 93 (71), 92 (37), 91 (100), 82 (62), 80 (29), 79 (75), 77 (61), 67 (20), 65 (19), 58 (24), 54 (21), 53 (27), 41 (34). ^1^H-NMR (C_6_D_6_, 500 MHz) and ^13^C-NMR (C_6_D_6_, 125 MHz): see Table 4.

*Methyl pin-2-en-8-oate* (**D**): C_11_H_16_O_2_; HREIMS: *m*/*z* 180.1137 (calcd. for C_11_H_16_O_2_, 180.1156); EI-MS 70 eV, *m*/*z* (rel. int.): 180 (13, M^•+^), 165 (9, M^•+^ − CH_3_), 148 (40, M^•+^ − MeOH), 139 (15), 133 (13), 125 (23), 121 (100), 120 (39), 119 (32), 112 (25), 105 (94), 93 (75), 91 (75), 88 (59), 79 (51), 77 (61), 65 (20), 53 (23), 43 (20). ^1^H-NMR (C_6_D_6_, 500 MHz) and ^13^C-NMR (C_6_D_6_, 125 MHz): see Table 5.

*Pin-2-en-8-oic* *acid* (**E**): C_10_H_14_O_2_; RI (DB-1/DB-WAX) 1288/2286; EI-MS 70 eV, *m*/*z* (rel. int.): 166 (4, M^•+^), 121 (100), 111 (18), 105 (76), 98 (25), 93 (69), 91 (73), 80 (11), 79 (61), 77 (62), 74 (41), 68 (35), 67 (28), 65 (6), 55 (13), 53 (16), 51 (5), 43 (14), 41 (36). ^13^C-NMR (C_6_D_6_, 125 MHz): see Table 4.

## Figures and Tables

**Table 1:** Chemical composition of essential oils of *Calocedrus decurrens* from Corsica.

No.	Compounds	RIa	RIp	RRF Calculated	L EO	OB EO	YB EO	Identification
1	1-(2-Methylene cyclopropyl) cyclopentene	870	1054	0.789	0.9	0.1	0.3	RI, MS
2	Tricyclene	920	1015	0.765	0.1	0.3	0.2	RI, MS
3	α-Thujene	922	1015	0.765	0.1	0.1	0.1	RI, MS
4	α-Pinene	932	1015	0.765	11.2	56.6	22.3	RI, MS, ^13^C-NMR
5	α-Fenchene	941	1047	0.765	0.7	0.2	0.2	RI, MS, ^13^C-NMR
6	Camphene	943	1063	0.765	0.2	0.5	0.2	RI, MS, ^13^C-NMR
7	Sabinene	964	1122	0.765	0.1	0.3	0.2	RI, MS
8	β-Pinene	970	1110	0.765	0.4	1.2	0.6	RI, MS, ^13^C-NMR
9	2-Pentylfuran	976	-	0.912	-	tr	-	RI, MS
10	Myrcene	981	1159	0.765	13.4	8.4	9.6	RI, MS, ^13^C-NMR
11	1,3,8-*p*-Menthatriene	995	-	0.779	-	tr	-	RI, MS
12	α-Phellandrene	996	1163	0.765	0.1	0.1	0.1	RI, MS
13	*p*-Methylanisole	998	1452	0.838	-	tr	-	RI, MS
14	Δ-3-Carene	1005	1146	0.765	31.3	5.2	11.1	RI, MS, ^13^C-NMR
15	α-Terpinene	1009	1178	0.765	0.2	0.1	0.2	RI, MS
16	*p*-Cymene	1011	1269	0.698	0.3	0.2	0.2	RI, MS, *¹³C-NMR*
17	Sylvestrene	1017	1199	0.765	0.5	0.1	0.2	RI, MS
18	Limonene *	1021	1200	0.765	6.4	5.1	5.5	RI, MS, ^13^C-NMR
19	β-Phellandrene *	1021	1209	0.765	1.7	1.2	1.4	RI, MS, ^13^C-NMR
20	γ-Terpinene	1048	1243	0.765	0.3	0.2	0.2	RI, MS
21	Methyl phenylethyl ether	1058	-	0.821	-	0.1	0.1	RI, MS
22	Fenchone	1068	1401	0.887	tr	0.1	0.1	RI, MS
23	*p*-Cymenene	1072	1434	0.709	0.3	0.1	0.2	RI, MS, *¹³C-NMR*
24	Terpinolene	1078	1280	0.765	6.9	1.5	3.2	RI, MS, ^13^C-NMR
25	Linalool	1083	1543	0.869	0.2	tr	0.2	RI, MS
26	Perillene	1086	1419	0.907	-	tr	tr	RI, MS
27	Pin-2-en-8-al (**C**)	1102	1460	0.907	1.6	0.8	2.0	RI, MS, 2D-NMR
28	Camphre	1120	1513	0.887	0.1	0.2	0.3	RI, MS
29	*trans*-Pinocarveol	1123	1549	0.876	-	0.1	-	RI, MS
30	*trans*-*p*-Menth-2-en-1-ol	1125	-	0.869	-	-	tr	RI, MS
31	*trans*-Verbenol	1128	1680	0.876	0.1	tr	0.1	RI, MS
32	Camphene hydrate	1132	1591	0.869	0.1	0.3	0.4	RI, MS
33	*trans*-Pinocamphone	1133	1507	0.887	-	-	tr	RI, MS
34	Myrtenyl methyl ether	1145	1380	0.868	tr	0.2	0.2	RI, MS, *¹³C-NMR*
35	Borneol	1150	1698	0.869	0.1	-	0.3	RI, MS
36	Isopinocamphone	1153	1547	0.887	-	-	tr	RI, MS
37	*p*-Methylacetophenone	1155	1776	0.839	0.1	-	-	RI, MS
38	*p*-Cymen-8-ol	1159	1803	0.809	0.2	0.2	0.3	RI, MS
39	Terpinen-4-ol	1161	1598	0.869	1.0	0.6	1.1	RI, MS, ^13^C-NMR
40	(*E*)-Dec-4-enal	1170	1537	0.869	0.7	0.2	0.6	RI, MS, ^13^C-NMR
41	α-Terpineol	1172	1692	0.869	0.2	0.3	0.3	RI, MS
42	Myrtenol	1178	1787	0.887	-	-	tr	RI, MS
43	Verbenone	1181	1708	0.907	0.3	0.4	0.6	RI, MS, ^13^C-NMR
44	α-Campholenol	1186	1782	0.887	0.1	0.3	0.5	RI, MS, ^13^C-NMR
45	Pin-2-en-8-ol (**A**)	1189	1800	0.887	4.2	4.5	10.3	RI, MS, 2D-NMR
46	Methyl pin-2-en-8-oate (**D**)	1207	1543	1.006	3.0	1.8	3.6	RI, MS, 2D-NMR
47	Thymyl methyl ether	1213	1589	0.798	-	-	tr	RI, MS
48	Carvone	1214	1733	0.907	0.1	0.3	0.5	RI, MS, ^13^C-NMR
49	Methyl campholenate	1222	1576	0.985	0.2	0.2	0.5	RI, MS, ^13^C-NMR
50	Carvacryl methyl ether	1225	1601	0.798	-	-	tr	RI, MS
51	Piperitone	1226	1730	0.887	0.3	0.3	0.5	RI, MS, ^13^C-NMR
52	(*Z*)-Dec-4-en-1-ol	1240	1789	0.852	-	-	tr	RI, MS
53	Bornyl acetate	1269	1576	0.958	0.4	0.5	0.7	RI, MS, ^13^C-NMR
54	Methyl myrtenate	1273	1685	1.006	2.3	1.6	3.5	RI, MS, ^13^C-NMR
55	(*E*,*E*)-Deca-2,4-dienal	1288	-	0.887	0.2	-	0.2	RI, MS
56	Myrtenyl acetate	1305	1680	0.976	-	-	tr	RI, MS
57	Pin-2-en-8-yl Acetate (**B**)	1310	1683	0.976	0.6	0.6	1.4	RI, MS, 2D-NMR
58	α-Terpinyl acetate	1332	1690	0.958	2.4	1.0	2.1	RI, MS, ^13^C-NMR
59	β-Elemene	1387	1589	0.751	-	0.5	0.3	RI, MS, ^13^C-NMR
60	(*E*)-β-Caryophyllene	1417	1596	0.751	-	0.1	0.2	RI, MS, *¹³C-NMR*
61	Thujopsene	1428	1617	0.751	-	0.2	0.3	RI, MS, *¹³C-NMR*
62	Prezizaene	1444	1630	0.751	-	0.1	0.1	RI, MS
63	α-Humulene	1447	1667	0.751	-	tr	tr	RI, MS
64	Selina-4,11-diene	1470	1670	0.751	-	tr	tr	RI, MS
65	β-Selinene	1481	1712	0.751	-	0.4	0.3	RI, MS, *¹³C-NMR*
66	α-Selinene	1490	1718	0.751	-	0.3	0.2	RI, MS, *¹³C-NMR*
67	β-Bisabolene	1500	1720	0.751	-	0.1	0.2	RI, MS, *¹³C-NMR*
68	γ-Cadinene	1506	1750	0.751	-	tr	0.1	RI, MS
69	γ-Cuprenene	1523	-	0.751	-	-	tr	RI, MS
70	β-Elemol	1534	2073	0.819	-	0.1	tr	RI, MS
71	Caryophyllene oxide	1569	1981	0.830	-	tr	0.1	RI, MS
72	Cedrol	1588	2105	0.819	0.2	0.6	1.1	RI, MS, ^13^C-NMR
73	γ-Eudesmol	1617	2158	0.819	-	tr	-	RI, MS
74	T-Cadinol	1625	2163	0.819	-	-	tr	RI, MS
75	β-Eudesmol	1634	2218	0.819	-	0.1	tr	RI, MS
76	Eudesm-11-en-4α-ol	1637	-	0.819	-	-	0.2	RI, MS
77	α-Eudesmol	1639	2209	0.819	-	0.1	tr	RI, MS
78	(*Z*)-Heptadec-8-ene	1676	-	0.723	-	-	tr	RI, MS
79	Manool oxide	1983	2334	0.795	-	0.1	tr	RI, MS
80	(*E*)-Biformene	2003	2377	0.744	-	0.2	0.1	RI, MS
81	Abietatriene	2035	2486	0.751	-	0.3	0.1	RI, MS
82	Sandaracopimarinal	2157	2789	0.810	-	0.2	0.1	RI, MS
83	Dehydroabietal	2222	-	0.774	-	0.3	0.1	RI, MS
	**Total**				**93.8**	**99.8**	**89.9**	

Order of elution and percentages are given on apolar column; except for those with an asterisk (*), percentages on polar column. RIa, RIp: retention indices measured on apolar (DB-1) and polar (DB-WAX) columns, respectively. L EO = Leaf EO; OB EO = Old Branches EO; YB EO = Young Branches EO. The relative proportions of each constituent were expressed in g/100 g. tr: traces (<0.05%); ^13^C-NMR (italic) = compounds identified in fractions of chromatography. 2D-NMR = 2D-NMR spectrum are provided in Figure S1.

**Table 2:** NMR data of pin-2-en-8-ol (**A**).

C	^13^C δ (ppm)	^1^H	^1^H δ (ppm) by HSQC	COSY ^1^H-^1^H	HMBC H → C	NOESY ^a^
1	43.02	1	1.96 (td, ^3^*J*, ^4^*J* = 5.8 Hz; ^4^*J* = 1.2 Hz)	3, 4b, 5, 7b	2, 3, 5, 6, 7, 8, 10	5, 8a, 8b, 10
2	144.08	-	-	-	-	-
3	117.22	3	5.21 (m, *J* = 1.5 Hz)	1, 4a, 4b, 10	1, 5, 10	4a, 4b, 9, 10
4	31.18	4a (*anti*)	2.21 (dm, ^2^*J* = 17.1 Hz; *J* = 2.4 Hz)	3, 4b, 5, 10	-	3, 4b, 5, 7a
		4b (*syn*)	2.08 (dm, ^2^*J* = 17.1 Hz; *J* = 2.4 Hz)	1, 3, 4a, 5, 10	2, 3	3, 4a, 5, 9
5	36.72	5	2.06 (m)	1, 4a, 4b, 7b	1	1, 4a, 4b, 7b, 8a, 8b
6	43.75	-	-	-	-	-
7	31.88	7a (*endo*)	1.23 (d, ^2^*J* = 8.7 Hz)	7b	1, 2, 4, 5, 6, 9	4a, 7b
		7b (*exo*)	2.16 (dt, ^2^*J* = 8.7 Hz; ^3^*J* = 5.8 Hz)	1, 5, 7a	1, 2, 4, 5	5, 7a, 8a, 8b
8	69.00	8a	3.54 (d, ^2^*J* = 10.6 Hz)	8b, 9	1, 5, 9	1, 5, 7b, 9
		8b	3.50 (d, ^2^*J* = 10.6 Hz)	8a, 9	1, 5, 9	1, 5, 7b, 9
9	16.03	9	0.95 (s)	8a, 8b	1, 5, 6, 8	3, 4b, 8a, 8b, 10
10	23.06	10	1.61 (m, *J* = 1.9 Hz)	3, 4a, 4b	1, 2, 3	1, 3, 9

^a^ Pure NOE correlations appear underlined.

**Table 3:** NMR data of pin-2-en-8-yl Acetate (**B**).

C	^13^C δ (ppm)	^1^H	^1^H δ (ppm) by HSQC	COSY ^1^H-^1^H	HMBC H → C	NOESY ^a^
1	43.28	1	1.98 (td, ^3^*J*, ^4^*J* = 5.7 Hz; ^4^*J* = 1.2 Hz)	3, 5, 7b	2, 3, 5, 8, 10	5, 7a, 7b, 8a, 8b
2	143.57	-	-	-	-	-
3	117.29	3	5.17 (m, *J* = 1.5 Hz)	1, 4a, 4b, 10	-	4b, 9, 10
4	30.94	4a (*anti*)	2.16 (dm, ^2^*J* = 17.4 Hz; *J* = 2.5 Hz)	3, 4b, 5, 10	-	4b, 10
		4b (*syn*)	2.03 (dm, ^2^*J* = 17.4 Hz; *J* = 2.5 Hz)	3, 4a, 5, 10	-	3, 4a, 5, 9, 10
5	37.08	5	2.10 (m)	1, 4a, 4b, 7b	-	1, 4b, 7b, 8a, 8b, 12
6	41.99	-	-	-	-	-
7	31.73	7a (*endo*)	1.21 (d, ^2^*J* = 8.9 Hz)	7b	1, 2, 4, 5, 6, 9	1, 7b
		7b (*exo*)	2.25 (dt, ^2^*J* = 8.9 Hz; ^3^*J* = 5.7 Hz)	1, 5, 7a	1, 2, 4, 5	1, 5, 7a, 8a, 8b, 12
8	70.58	8a	4.32 (d, ^2^*J* = 11.1 Hz)	8b, 9	1, 5, 6, 9, 11	1, 5, 7b, 9
		8b	4.26 (d, ^2^*J* = 11.1 Hz)	8a, 9	1, 5, 6, 9, 11	1, 5, 7b, 9
9	16.40	9	0.96 (s)	8a, 8b	1, 5, 6, 8	3, 4b, 8a, 8b, 12
10	22.95	10	1.57 (m, *J* = 1.9 Hz)	3, 4a, 4b	1, 2, 3, 4	3, 4a, 4b
11	170.48	-	-	-	-	-
12	20.53	12	1.72 (s)	-	8, 11	5, 7b, 9

^a^ Pure NOE correlations appear underlined.

**Table 4:** NMR data of pin-2-en-8-al (**C**) and pin-2-en-8-oic acid (**E**).

C	^13^C δ (ppm) of C	^1^H of C	^1^H δ (ppm) by HSQC of C	^13^C δ (ppm) of E
1	42.86	1	2.29 (td, ^3^*J*, ^4^*J* = 5.7 Hz; ^4^*J* = 1.4 Hz)	45.31
2	142.26	-	-	142.08
3	117.99	3	5.12 (m, *J* = 1.6 Hz)	117.21
4	30.27	4a (*anti*)	2.08 (dm, ^2^*J* = 17.8 Hz; *J* = 2.5 Hz)	30.25
		4b (*syn*)	1.85 (dm, ^2^*J* = 17.8 Hz; *J* = 2.5 Hz)	
5	36.77	5	2.40 (md, ^4^*J* = 1.2 Hz)	39.35
6	53.25	-	-	49.14
7	30.76	7a (*endo*)	1.10 (d, ^2^*J* = 8.7 Hz)	32.06
		7b (*exo*)	2.06 (dt, ^2^*J* = 8.7 Hz; ^3^*J* = 5.7 Hz)	
8	204.19	8	9.62 (s)	184.91
9	12.24	9	0.73 (s)	16.20
10	22.70	10	1.49 (m, *J* = 2.0 Hz)	22.77

**Table 5:** NMR data of methyl pin-2-en-8-oate (**D**).

C	^13^C δ (ppm)	^1^H	^1^H δ (ppm) by HSQC	COSY ^1^H-^1^H	HMBC H → C	NOESY ^a^
1	45.44	1	2.77 (td, ^3^*J*, ^4^*J* = 5.6 Hz; ^4^*J* = 1.4 Hz)	3, 5, 7b	2, 3, 5, 6, 7, 8, 10	5, 7b, 10
2	142.22	-	-	-	-	-
3	117.19	3	5.14 (m, *J* = 1.5 Hz)	1, 4a, 4b, 10	-	4a, 4b, 9, 10
4	30.32	4a (*anti*)	2.15 (dm, ^2^*J* = 17.6 Hz; *J* = 2.4 Hz)	3, 4b, 5, 10	1, 2, 5, 6	3, 4b, 7a, 10
		4b (*syn*)	2.03 (dm, ^2^*J* = 17.6 Hz; *J* = 2.4 Hz)	3, 4a, 5, 10	2, 3, 5, 6, 7	3, 4a, 9, 10
5	39.46	5	2.87 (md, ^4^*J* = 1.2 Hz)	1, 4a, 4b, 7b	-	1, 7b
6	49.05	-	-	-	-	-
7	32.05	7a (*endo*)	1.23 (d, ^2^*J* = 8.6 Hz)	7b	1, 2, 4, 5, 6, 9	4a, 7b
		7b (*exo*)	2.16 (dt, ^2^*J* = 8.6 Hz; ^3^*J* = 5.6 Hz)	1, 5, 7a	1, 2, 4, 5, 6	1, 5, 7a, 11
8	178.28	-	-	-	-	-
9	16.24	9	1.11 (s)	-	1, 5, 6, 8	3, 4b, 10
10	22.85	10	1.57 (m, *J* = 1.9 Hz)	3, 4a, 4b	1, 2, 3	1, 3, 4a, 4b, 9
11	51.47	11	3.43 (s)	-	8	7b

^a^ Pure NOE correlations appear underlined.

## Supplementary Materials

The following are available online, Figure S1: 1D- and 2D-NMR data for pin-2-en-8-ol (**A**), pin2-en-8-yl Acetate (**B**), pin-2-en-8-al (**C**) and methyl pin-2-en-8-oate (**D**).
