# Supplementary material for: New Pinane Derivatives Found in Essential Oils of Calocedrus decurrens
Source: Molecules. 2017 Jun 2;22(6):921. doi: 10.3390/molecules22060921 (PMC6152735; doi:10.3390/molecules22060921)
Supplement: Supplementary file 1 [file molecules-22-00921-s001.pdf]

Figure S1. 1D- and 2D-NMR data for pin-2-en-8-ol **(A)**, pin-2-en-8-yl acetate **(B)**, pin-2-en-8-al **(C)** and methyl pin-2-en-8-oate **(D)**.

1D- and 2D-NMR data for Pin-2-en-8-ol (A)

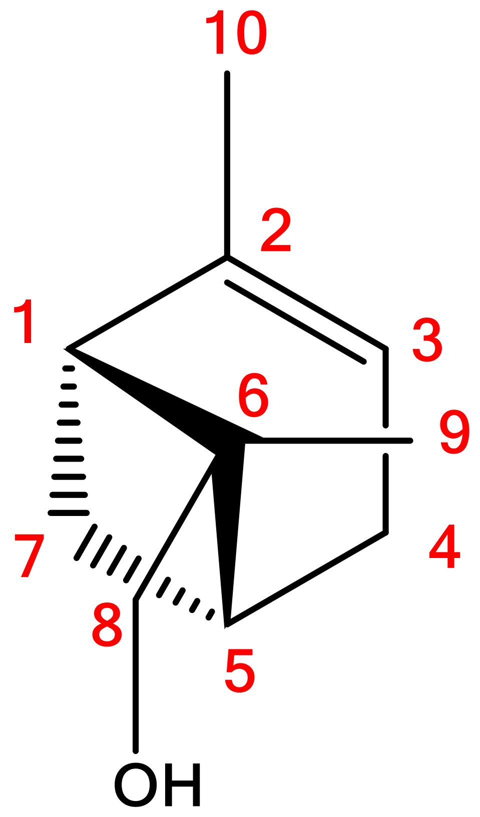

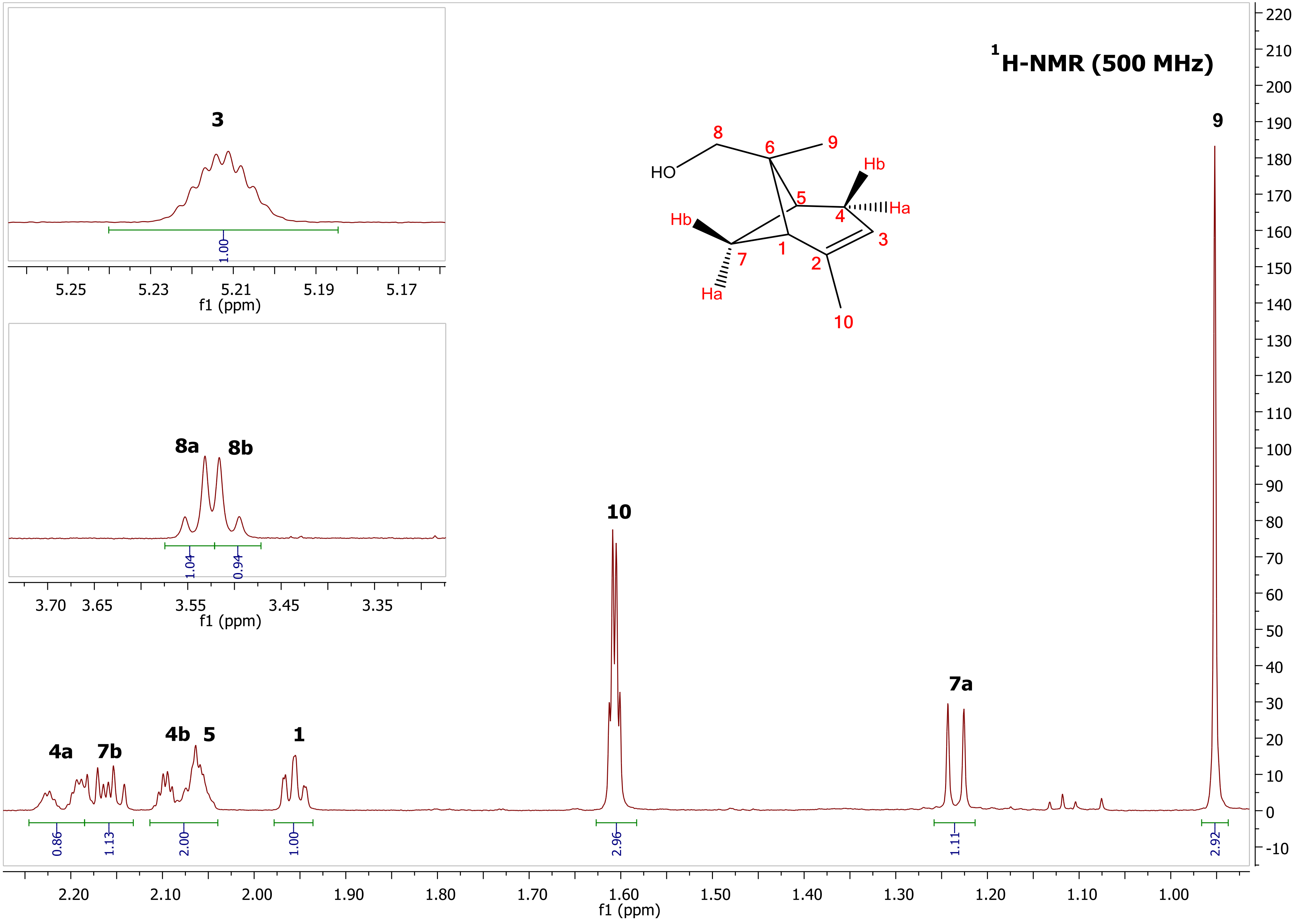

1H-NMR (500 MHz)
3
8a
8b
10
9
7a
4a
7b
4b
5
1
HO
8
6
9
Hb
5
4
Ha
Hb
7
1
2
3
Ha
10
f1 (ppm)

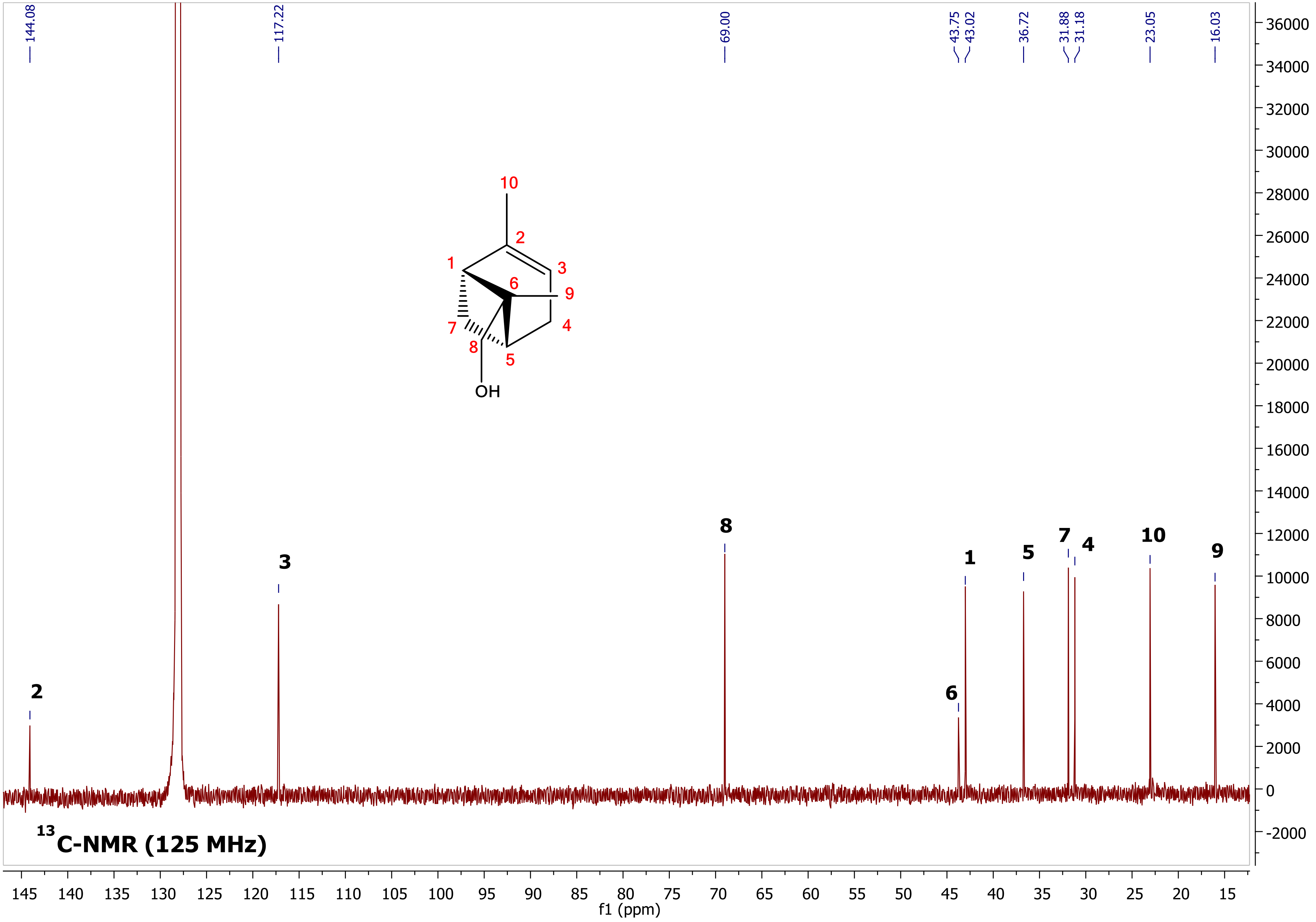

144.08
117.22
69.00
43.75
43.02
36.72
31.88
31.18
23.05
16.03
10
2
1
3
6
9
7
4
8
5
OH
2
3
8
6
1
5
7
4
10
9
f1 (ppm)
13C-NMR (125 MHz)

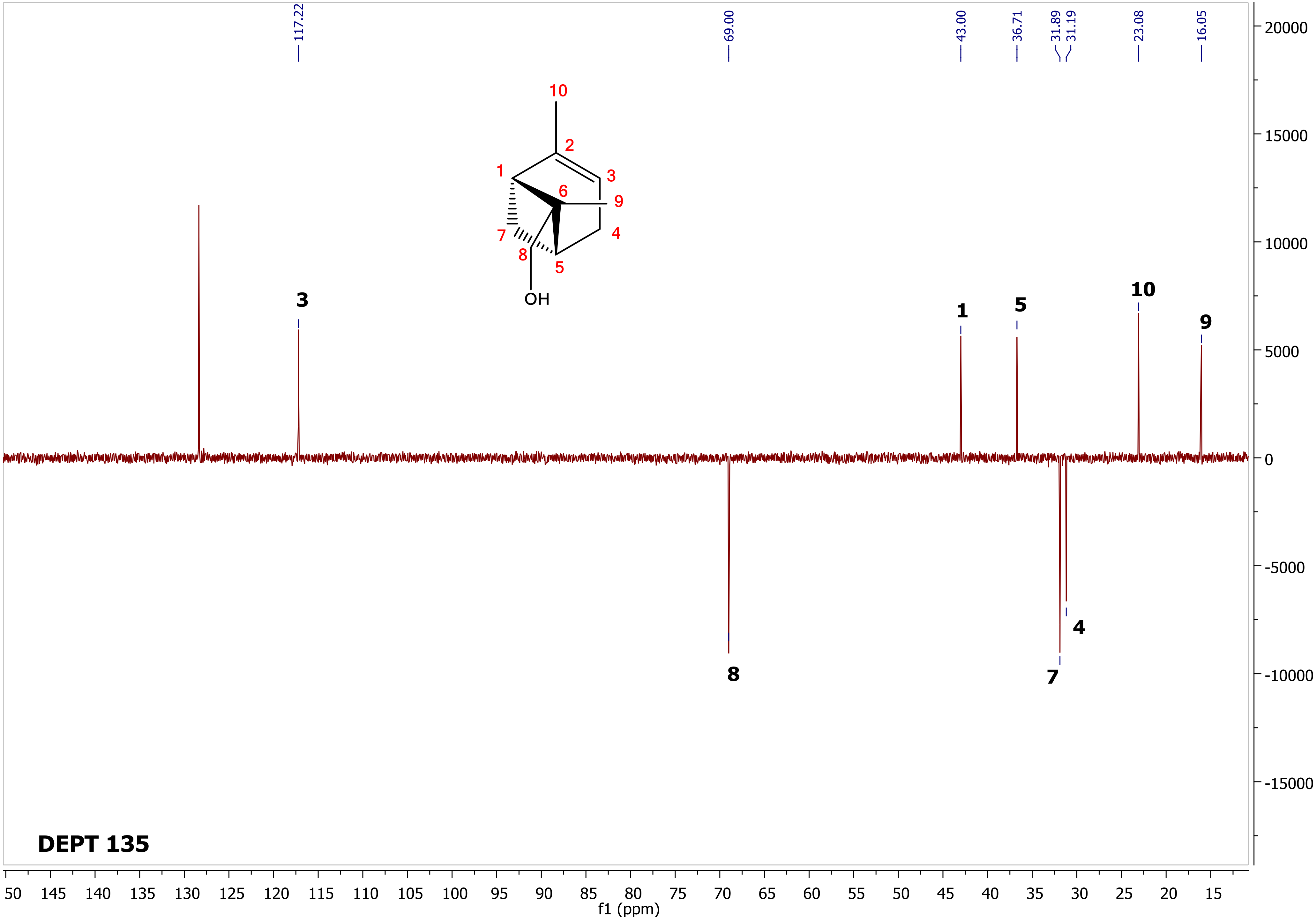
117.22
69.00
43.00
36.71
31.89
31.19
23.08
16.05
10
2
1
3
6
9
7
4
8
5
OH
3
1
5
10
9
8
7
4
DEPT 135
20000
15000
10000
5000
0
-5000
-10000
-15000
50
145
140
135
130
125
120
115
110
105
100
95
90
85
80
75
70
65
60
55
50
45
40
35
30
25
20
15
f1 (ppm)

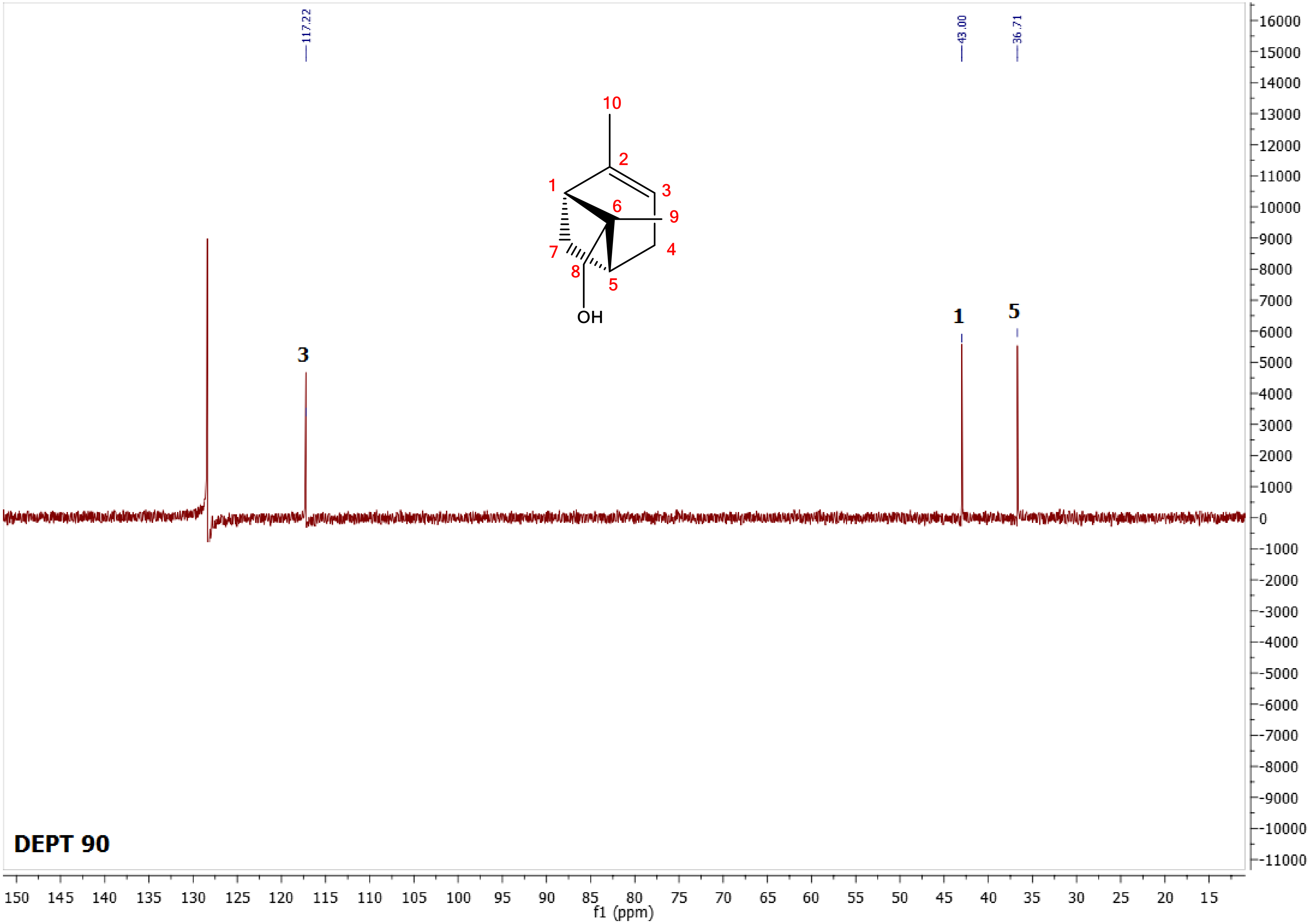
117.22
43.00
36.71
10
2
1
3
6
9
7
4
8
5
OH
3
1
5
DEPT 90
f1 (ppm)

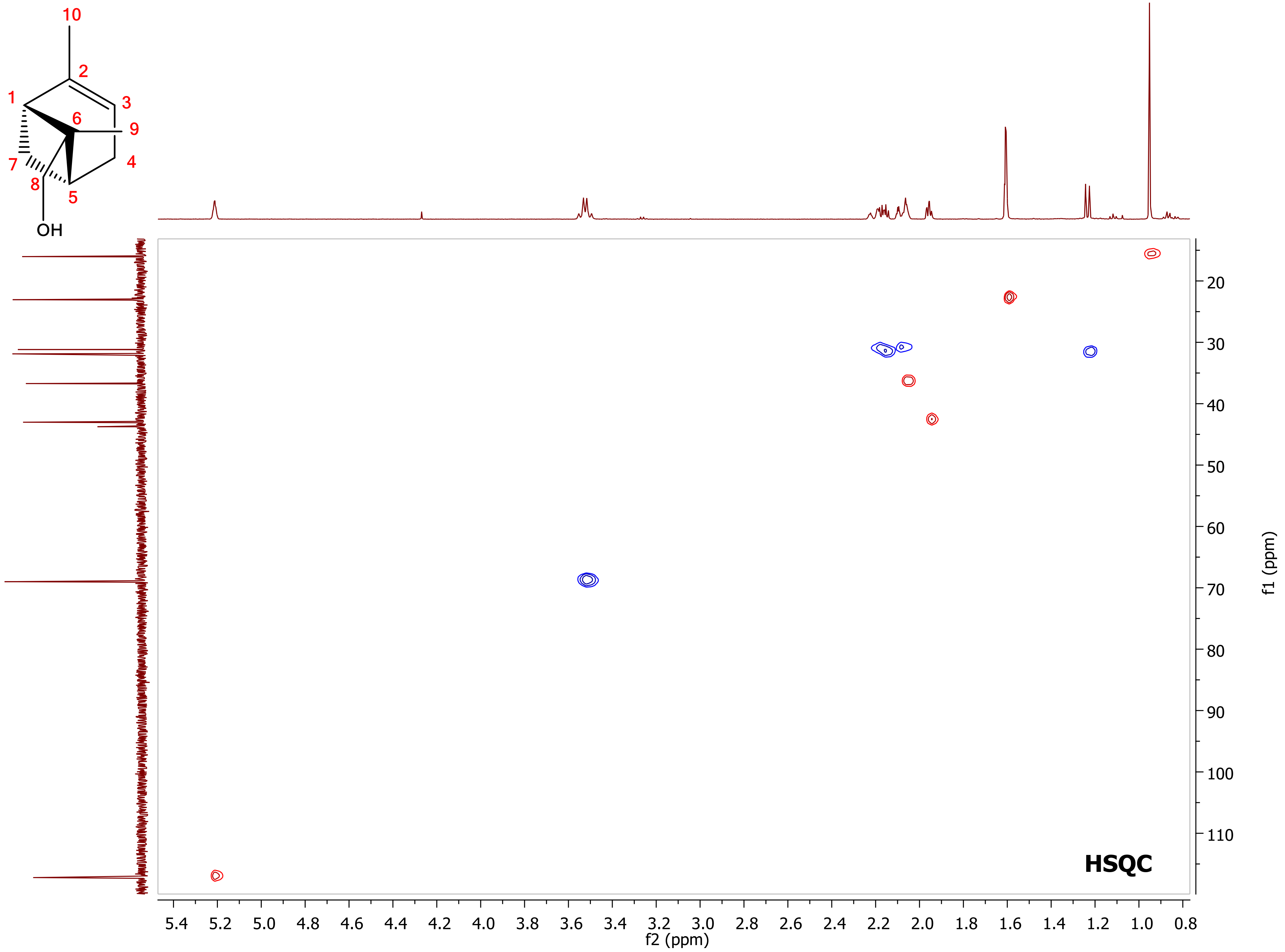
10
2
1
3
6
9
7
4
8
5
OH
HSQC
f2 (ppm)
f1 (ppm)

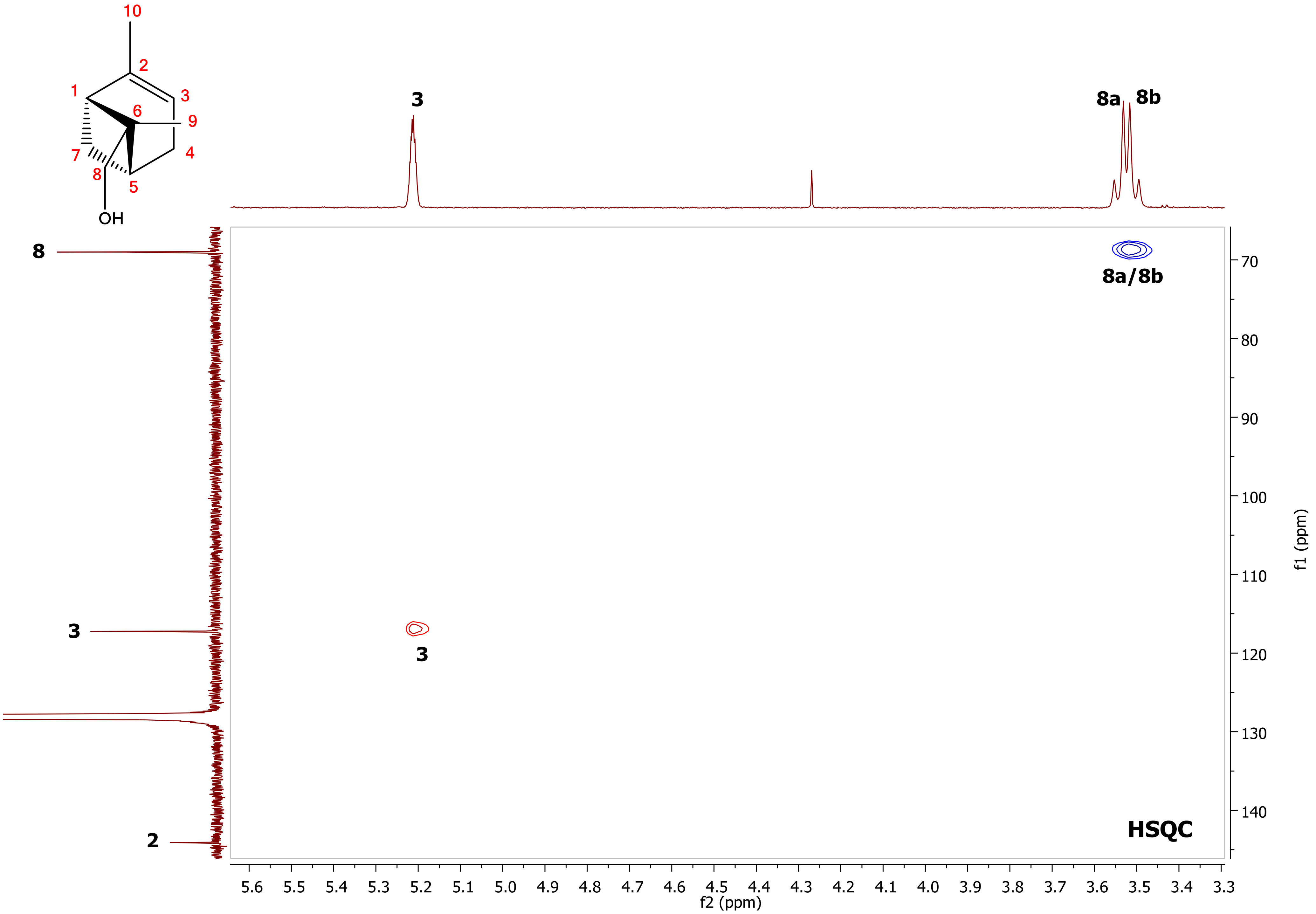
10
2
1
3
6
9
7
4
8
5
OH
3
8a
8b
8
8a/8b
3
2
HSQC
f1 (ppm)
f2 (ppm)
5.6
5.5
5.4
5.3
5.2
5.1
5.0
4.9
4.8
4.7
4.6
4.5
4.4
4.3
4.2
4.1
4.0
3.9
3.8
3.7
3.6
3.5
3.4
3.3
70
80
90
100
110
120
130
140

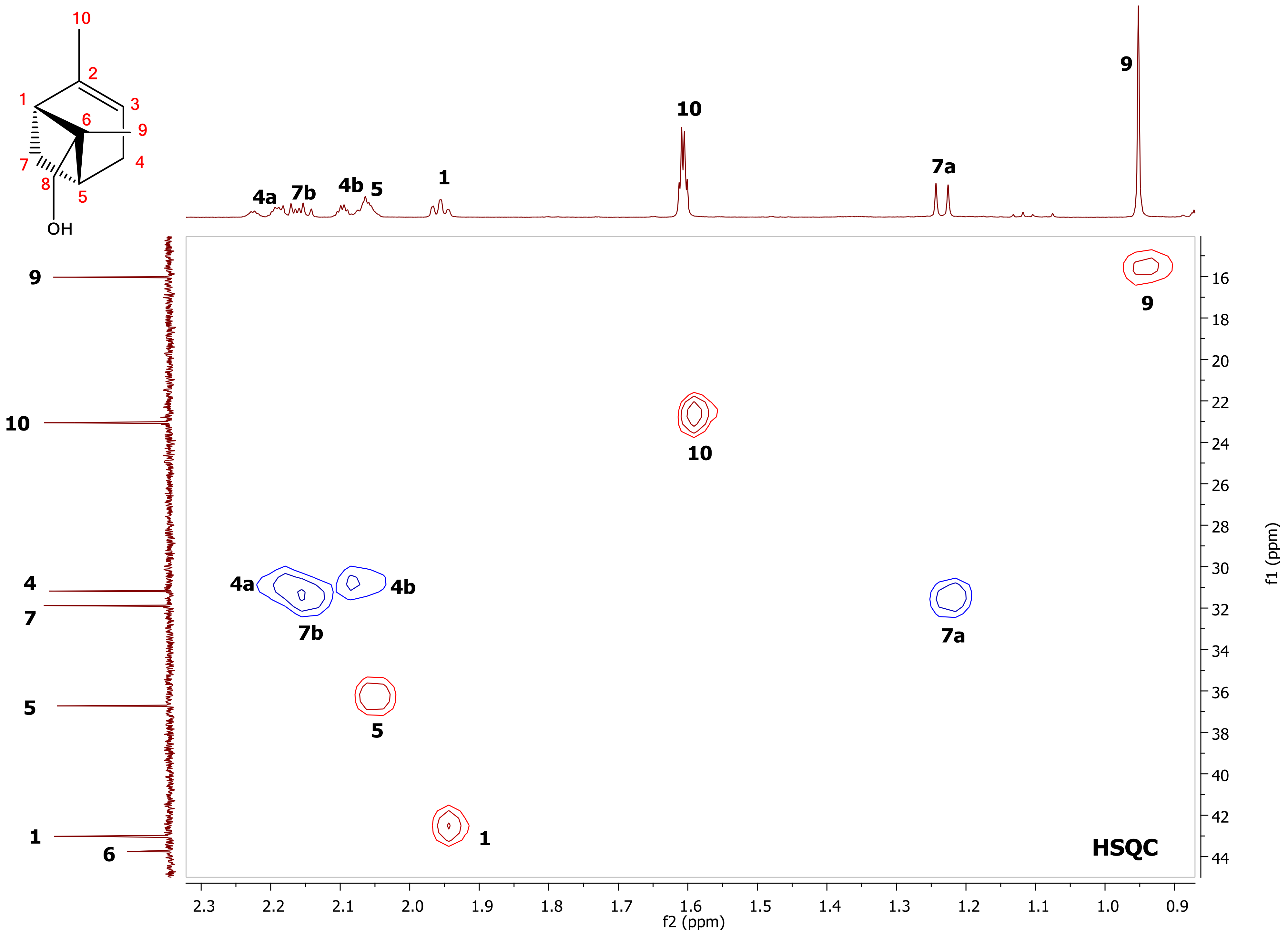

10
2
1
3
6
9
7
4
8
5
OH
9
10
7a
4a
7b
4b
5
1
9
10
4
7
5
1
6
4a
4b
7b
7a
5
1
HSQC
f1 (ppm)
f2 (ppm)

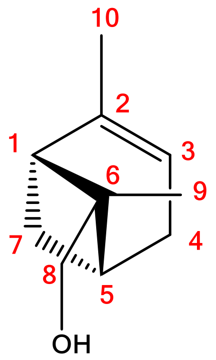
10
2
1
3
6
9
7
4
8
5
OH

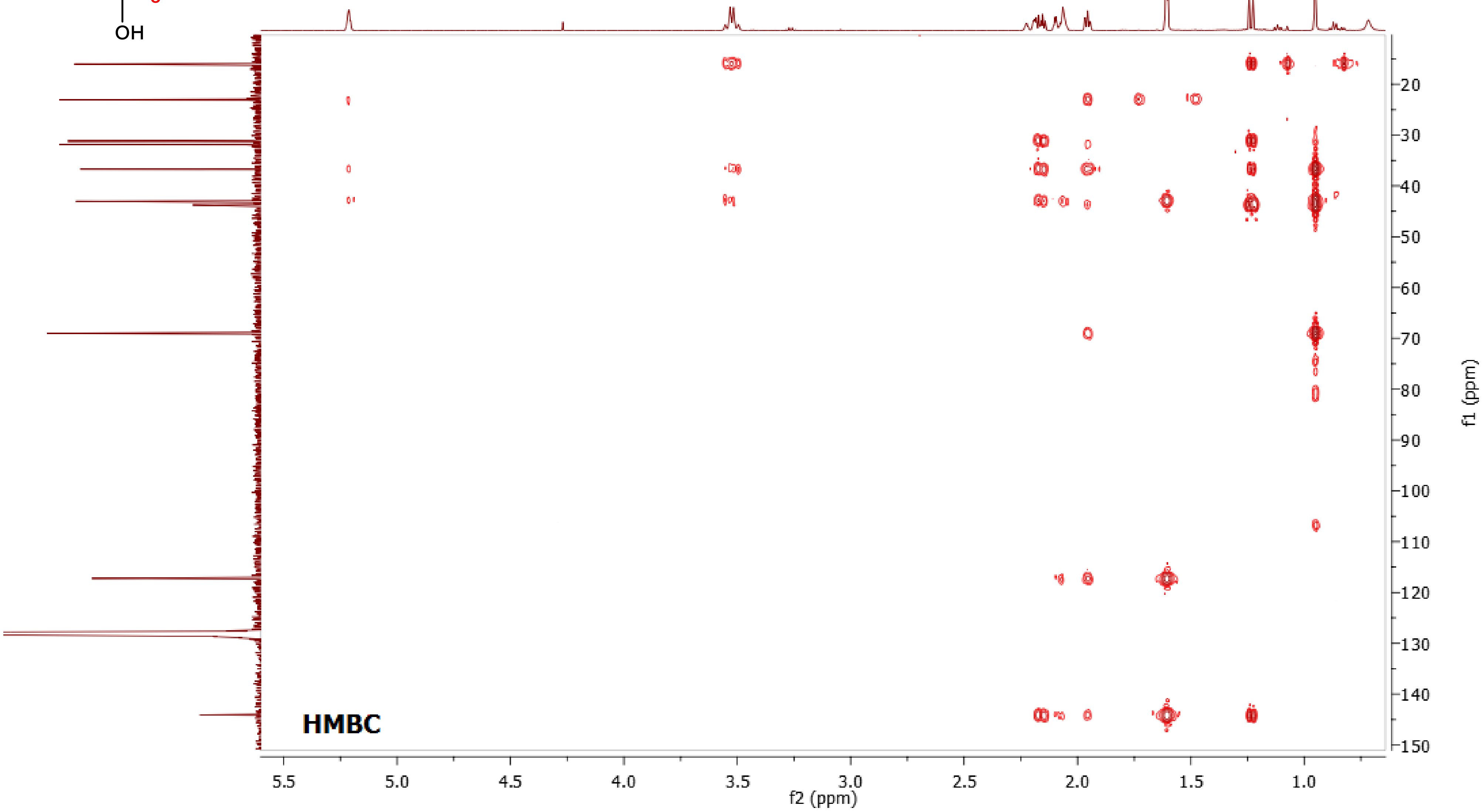
HMBC
f2 (ppm)
f1 (ppm)
5.5
5.0
4.5
4.0
3.5
3.0
2.5
2.0
1.5
1.0
20
30
40
50
60
70
80
90
100
110
120
130
140
150

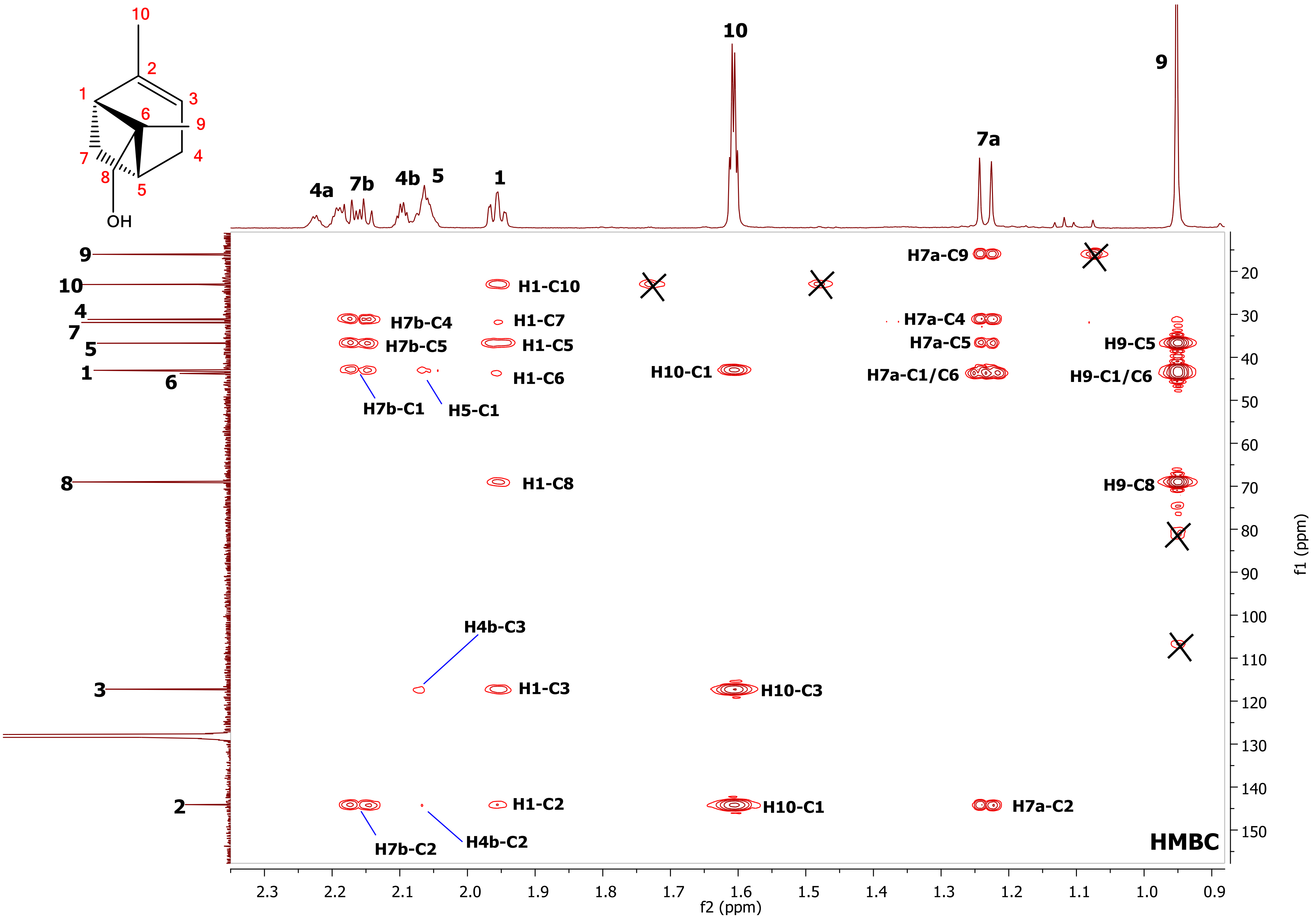

10
2
1
3
6
9
7
4
8
5
OH
4a
7b
4b
5
1
10
7a
9
H7a-C9
H1-C10
H7b-C4
H1-C7
H7a-C4
H7b-C5
H1-C5
H7a-C5
H9-C5
H10-C1
H1-C6
H7a-C1/C6
H9-C1/C6
H7b-C1
H5-C1
H1-C8
H9-C8
H4b-C3
H1-C3
H10-C3
H1-C2
H10-C1
H7a-C2
H7b-C2
H4b-C2
HMBC
f2 (ppm)
f1 (ppm)

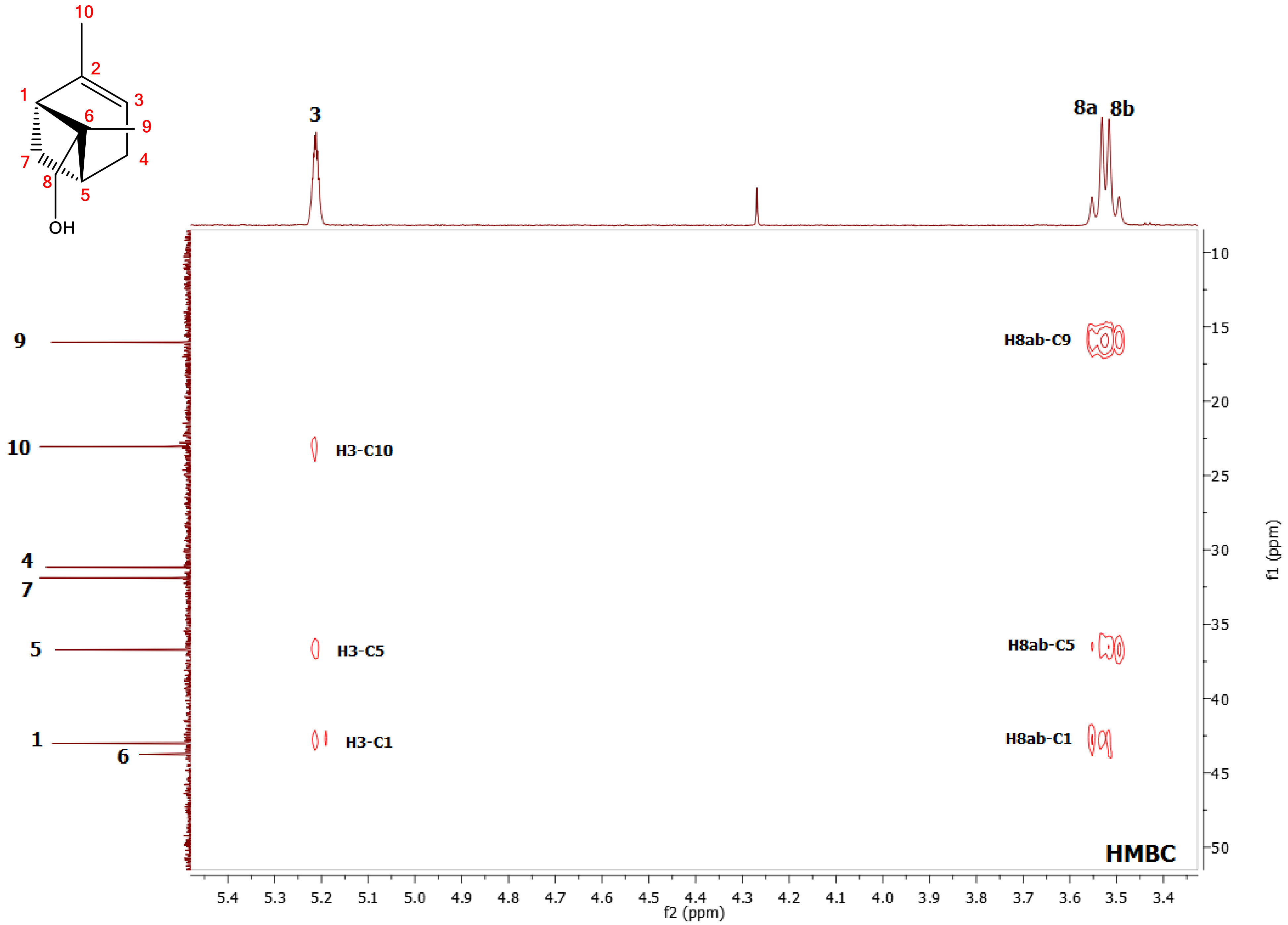
10
2
1
3
6
9
7
4
8
5
OH
3
8a
8b
9
10
4
7
5
1
6
H8ab-C9
H3-C10
H3-C5
H8ab-C5
H3-C1
H8ab-C1
HMBC
f2 (ppm)
f1 (ppm)

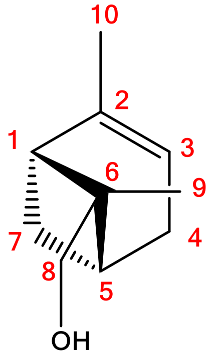

10
2
1
3
6
9
7
4
8
5
OH

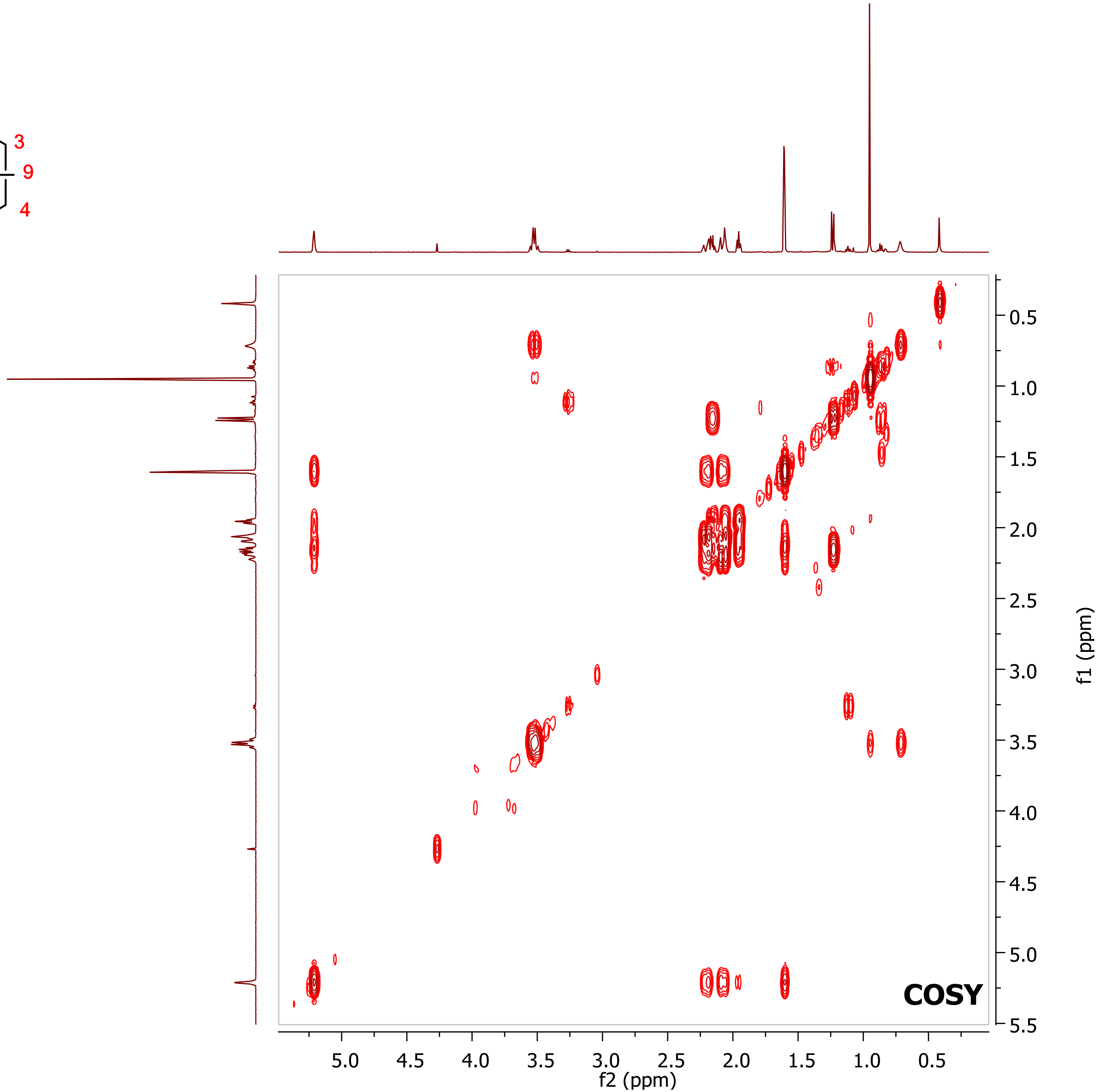

COSY
f2 (ppm)
f1 (ppm)
5.0
4.5
4.0
3.5
3.0
2.5
2.0
1.5
1.0
0.5
5.5

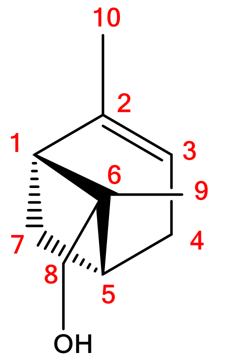
10
2
1
3
6
9
7
4
8
5
OH

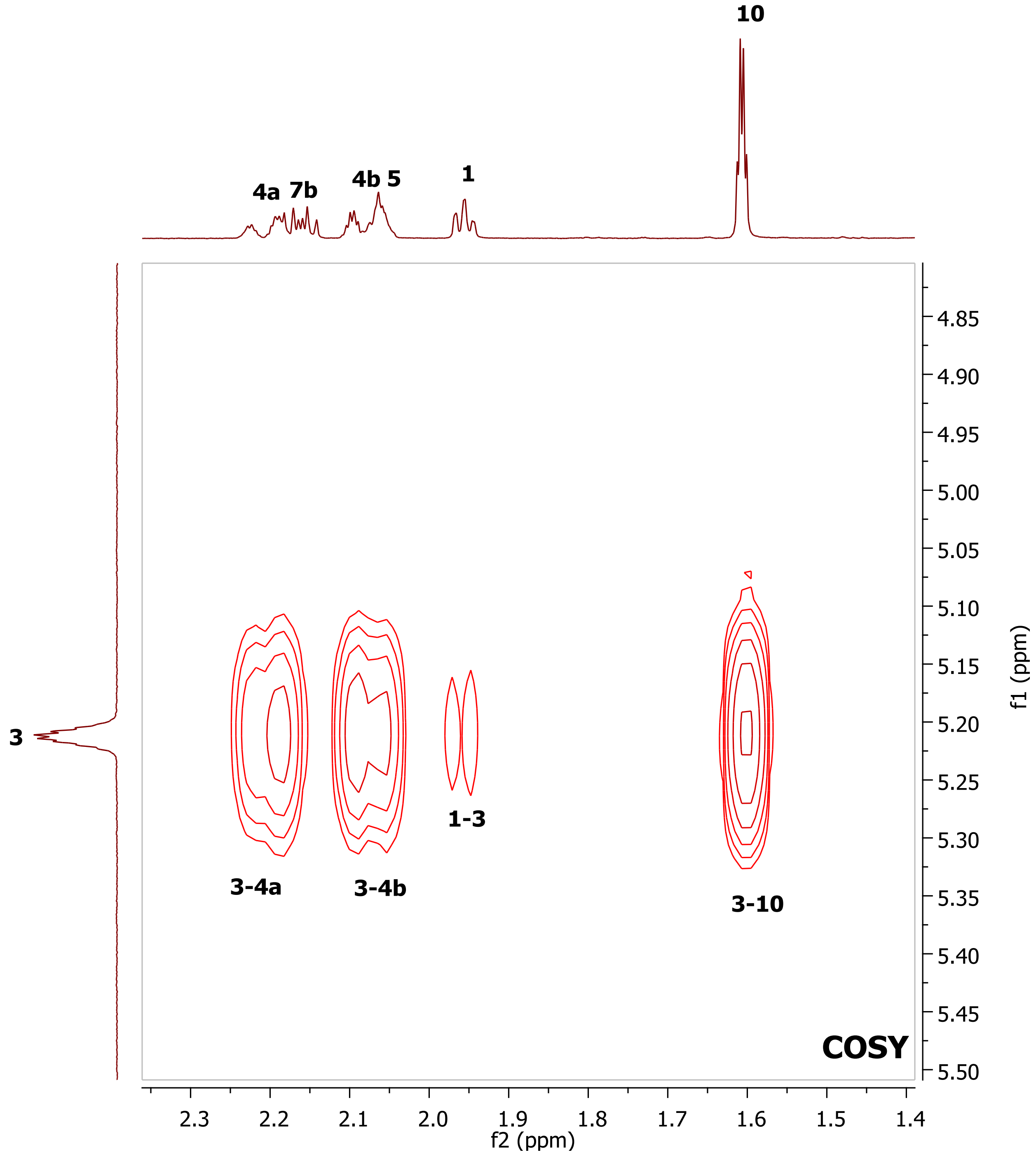
10
4a 7b
4b 5
1
3
3-4a
3-4b
1-3
3-10
COSY
4.85
4.90
4.95
5.00
5.05
5.10
5.15
5.20
5.25
5.30
5.35
5.40
5.45
5.50
f1 (ppm)
2.3
2.2
2.1
2.0
1.9
1.8
1.7
1.6
1.5
1.4
f2 (ppm)

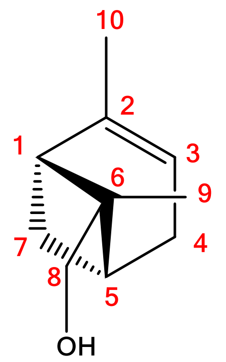

10
2
1
3
6
9
7
4
8
5
OH

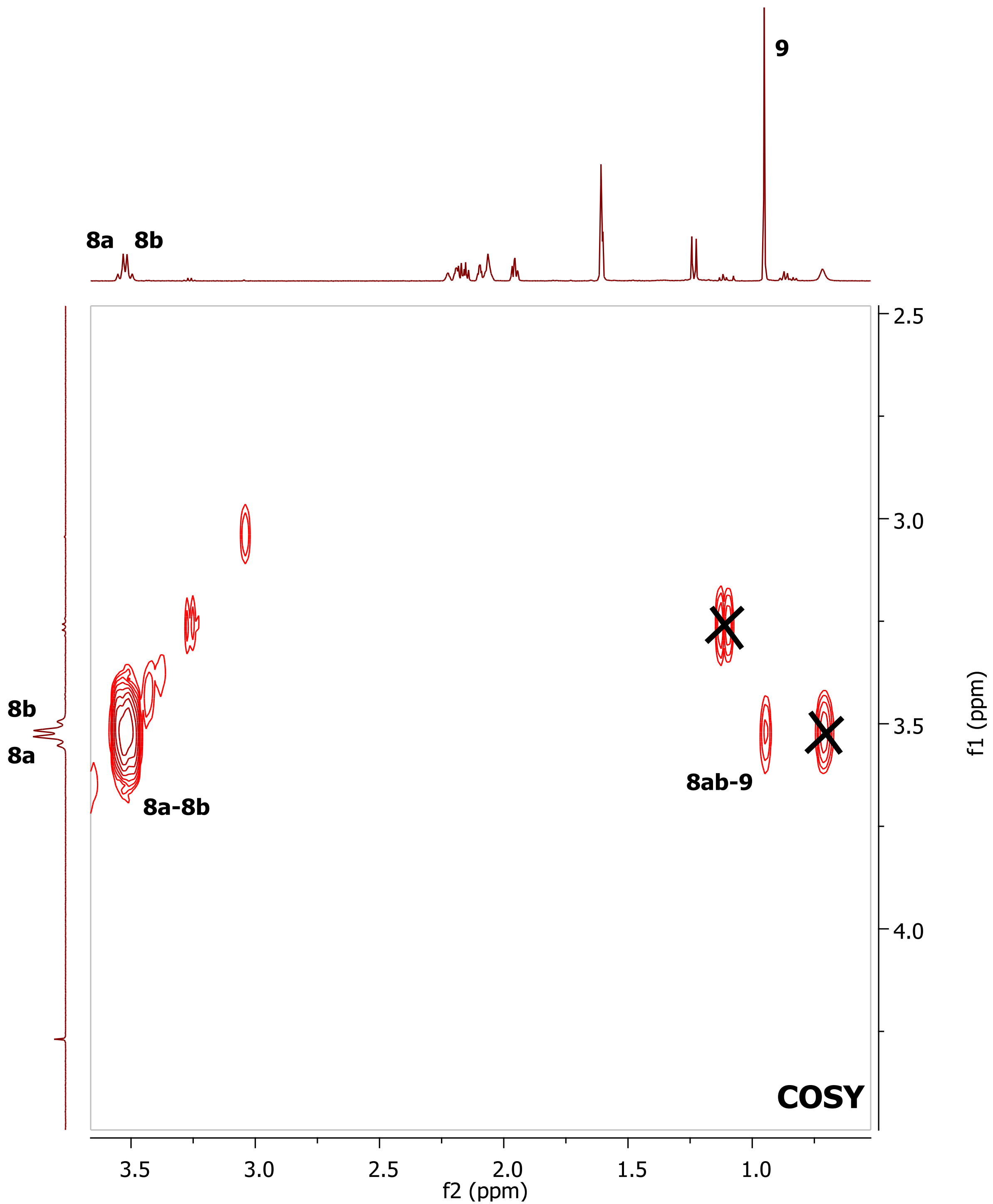

8a
8b
9
8b
8a
8a-8b
8ab-9
COSY
3.5
3.0
2.5
2.0
1.5
1.0
f2 (ppm)
2.5
3.0
3.5
4.0
f1 (ppm)

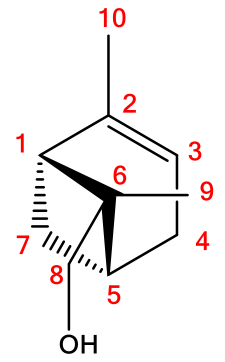

10
2
1
3
6
9
7
4
8
5
OH

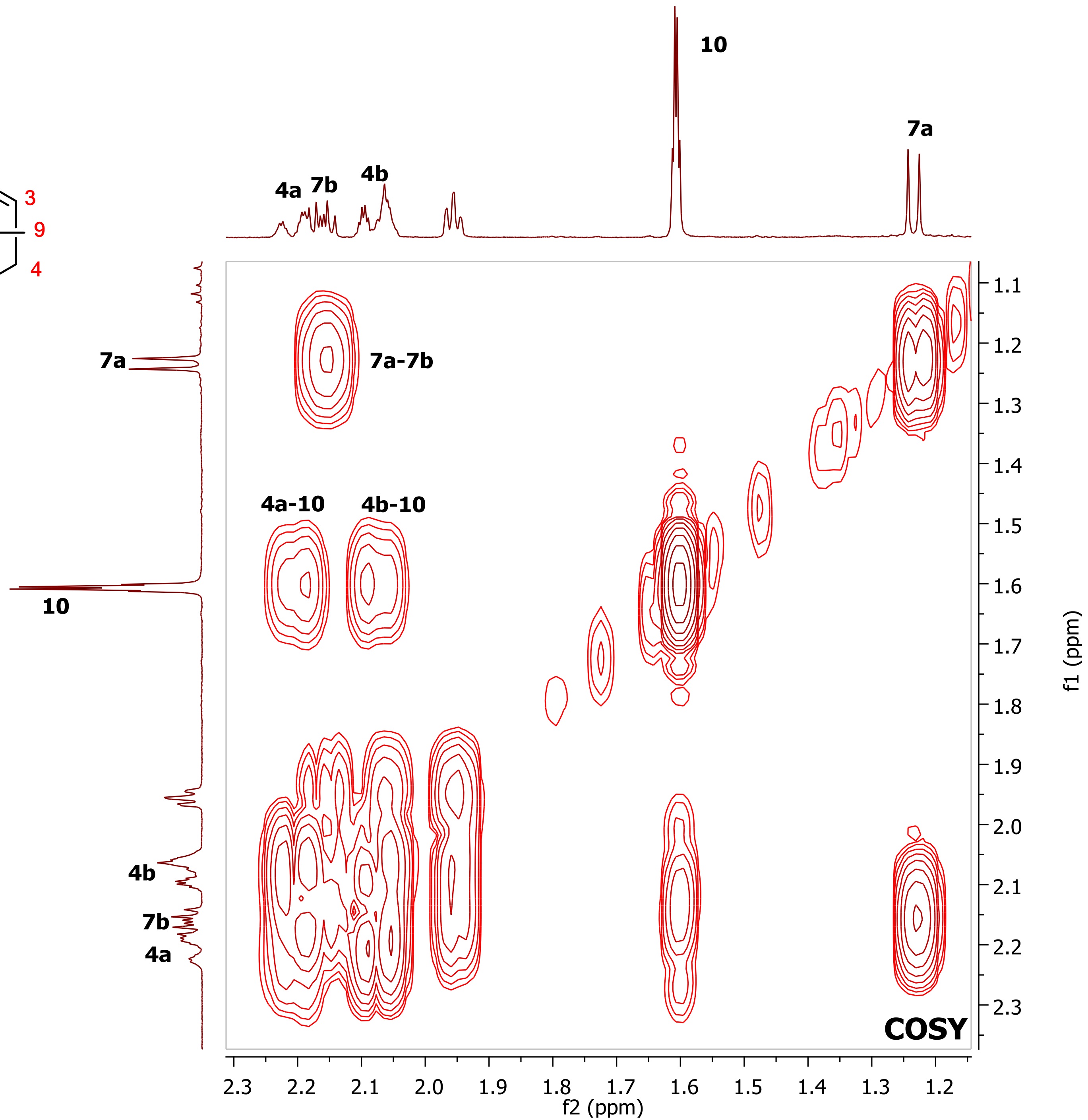

4a
7b
4b
10
7a
7a-7b
4a-10
4b-10
COSY
f2 (ppm)
f1 (ppm)

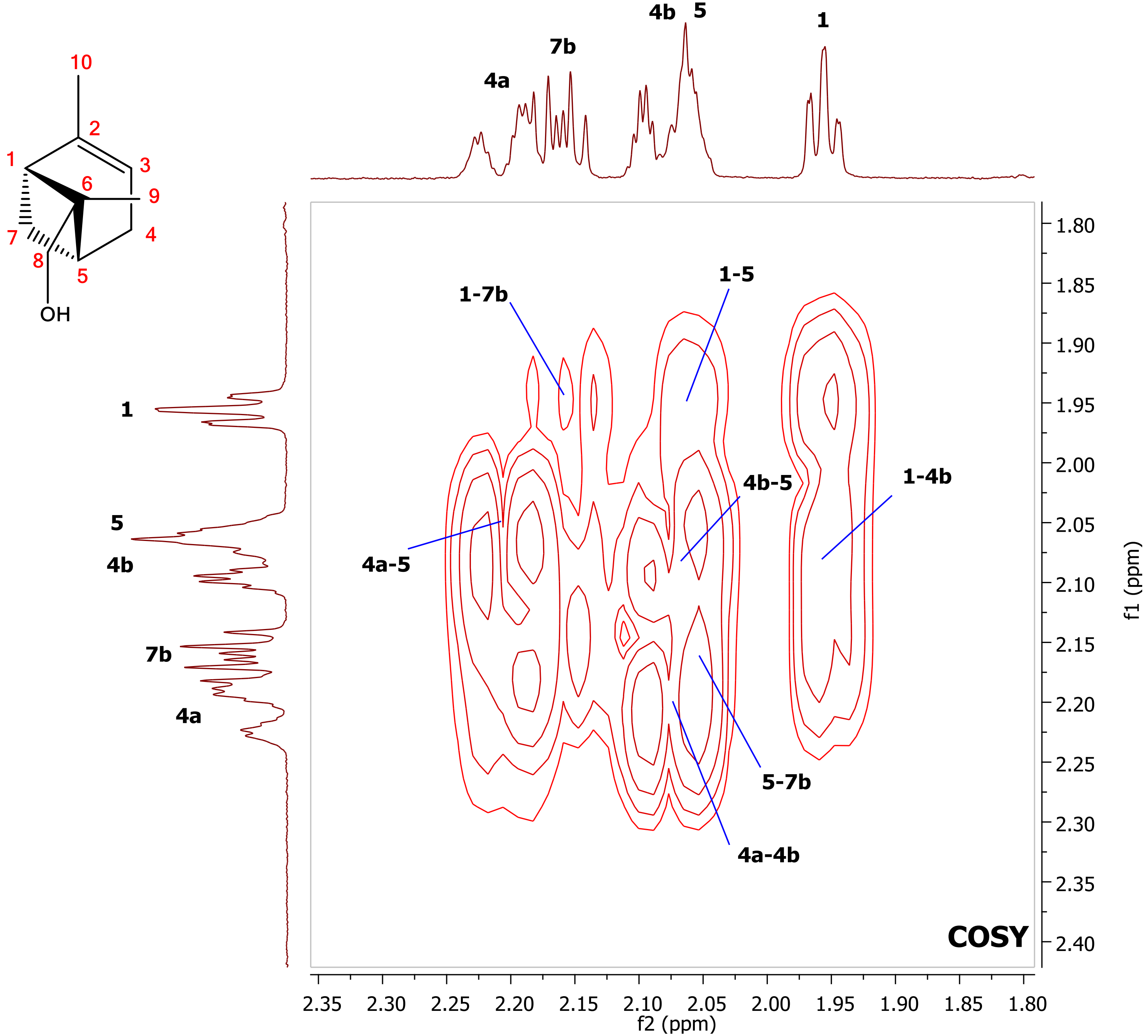
10
2
1
3
6
9
7
4
8
5
OH
4b
5
1
7b
4a
1
5
4b
7b
4a
1-7b
1-5
4b-5
1-4b
4a-5
5-7b
4a-4b
COSY
f1 (ppm)
f2 (ppm)
2.35
2.30
2.25
2.20
2.15
2.10
2.05
2.00
1.95
1.90
1.85
1.80
2.40

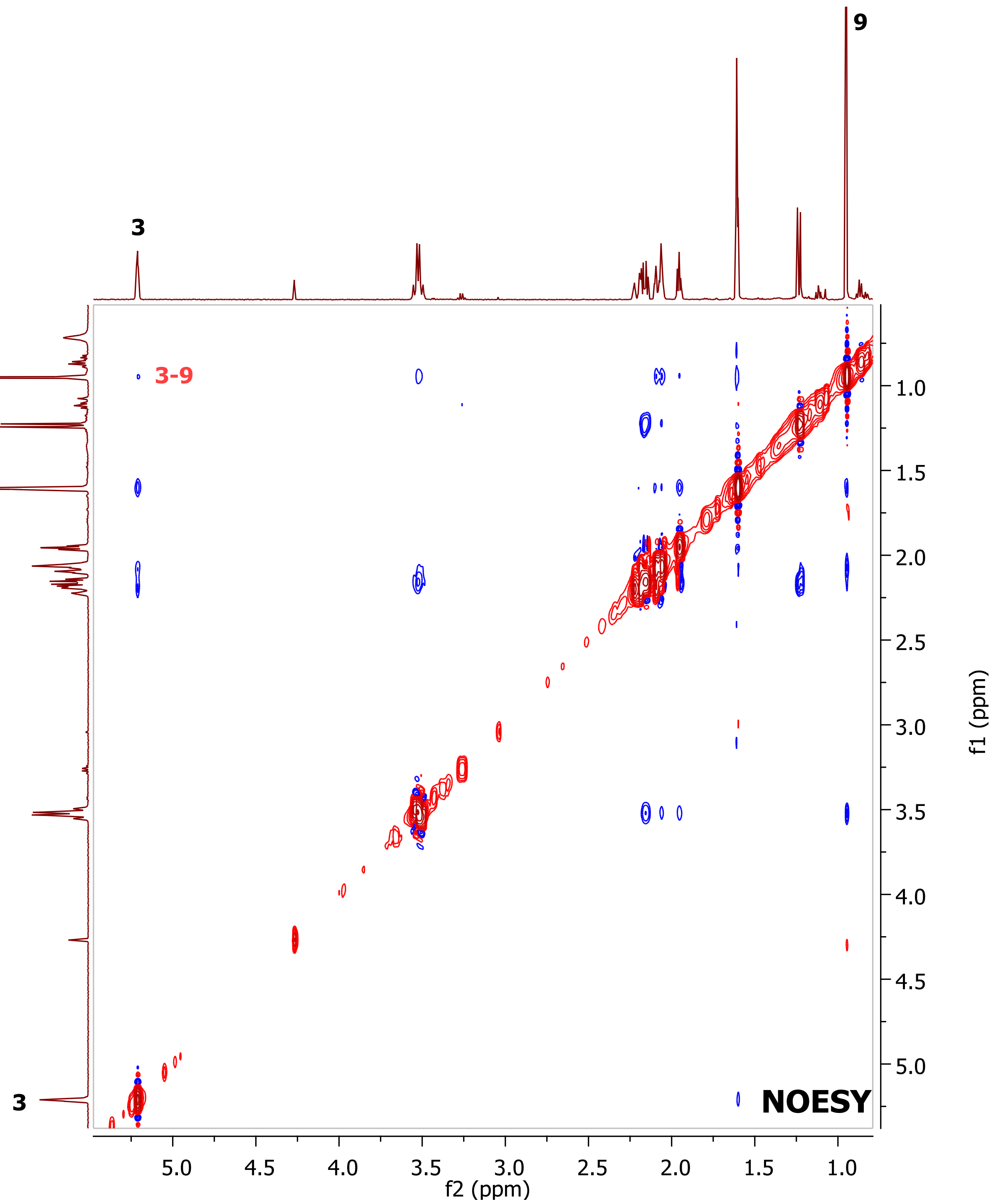

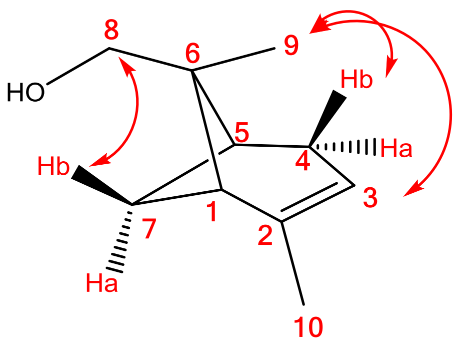

Key NOEs for compound A

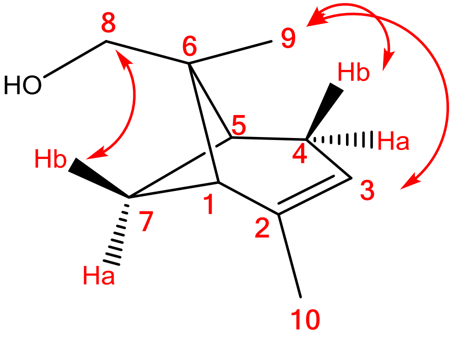

Key NOEs for compound A

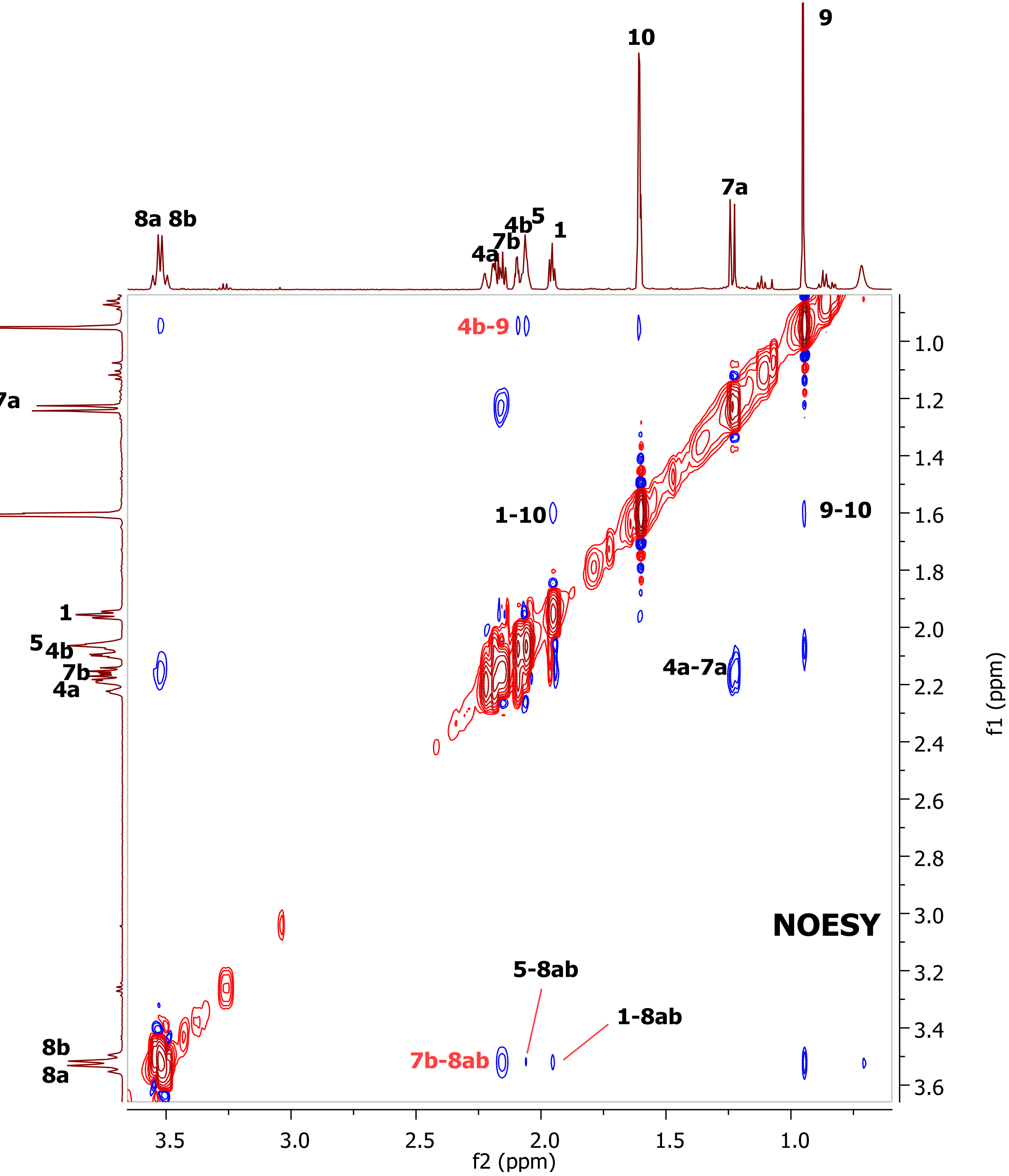

1D- and 2D-NMR data for Pin-2-en-8-yl acetate (B)

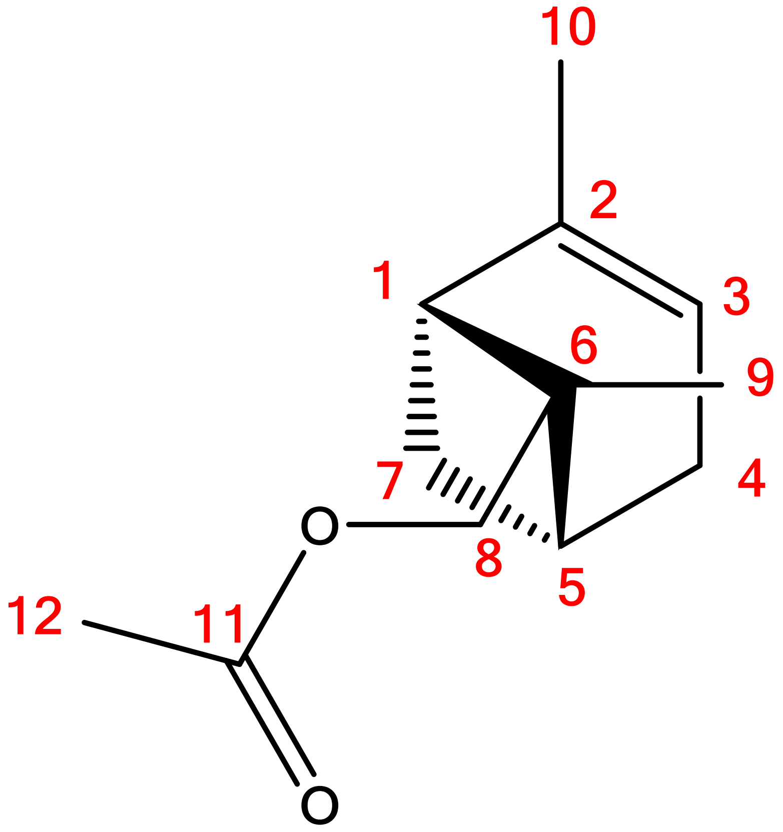

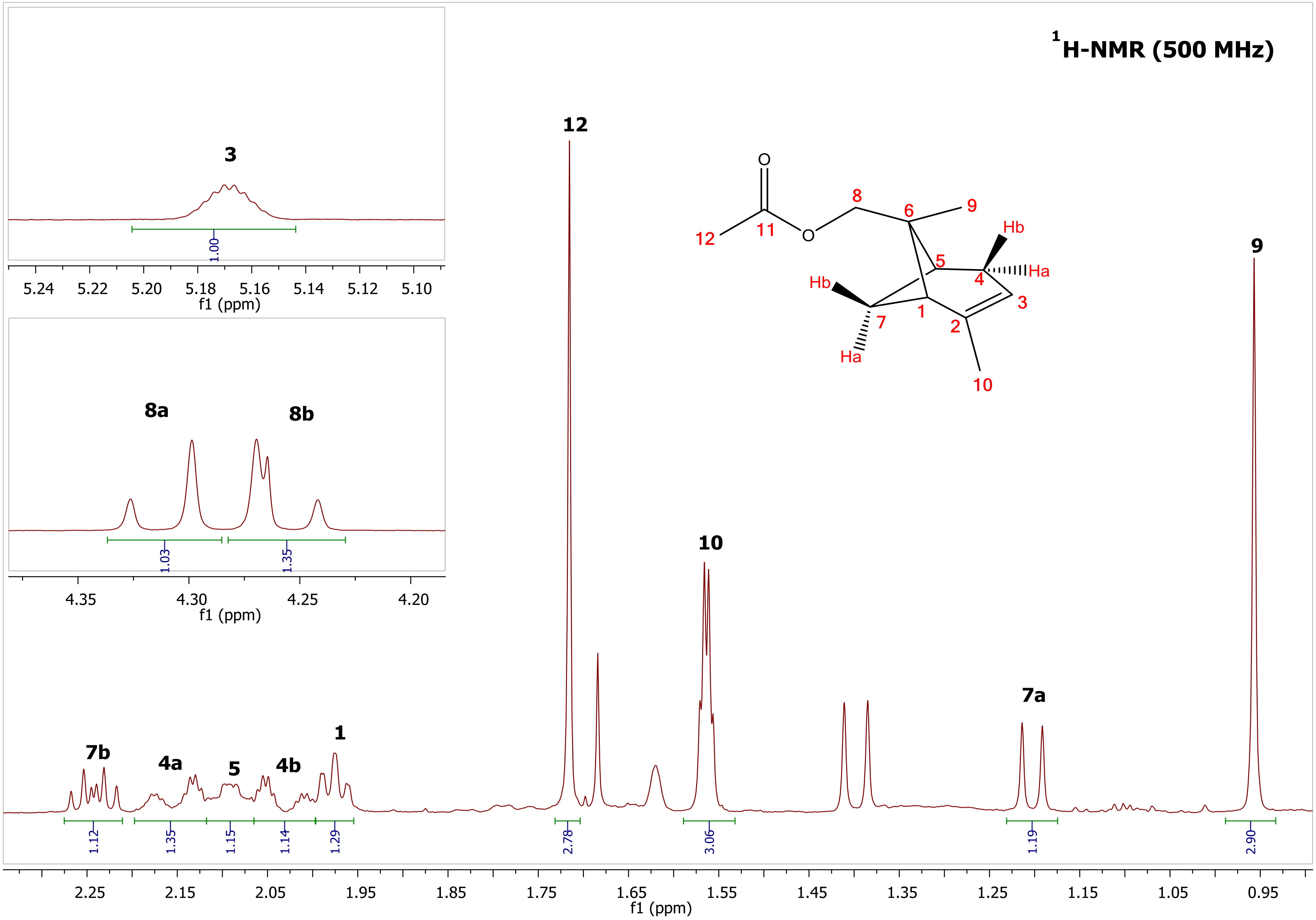

1H-NMR (500 MHz)
12
10
9
7a
7b
4a
5
4b
1
3
8a
8b
O
8
6
9
Hb
12
11
O
5
4
Ha
Hb
7
1
3
2
Ha
10
1.00
1.03
1.35
1.12
1.35
1.15
1.14
1.29
2.78
3.06
1.19
2.90
5.24
5.22
5.20
5.18
5.16
5.14
5.12
5.10
f1 (ppm)
4.35
4.30
4.25
4.20
f1 (ppm)
2.25
2.15
2.05
1.95
1.85
1.75
1.65
1.55
1.45
1.35
1.25
1.15
1.05
0.95
f1 (ppm)

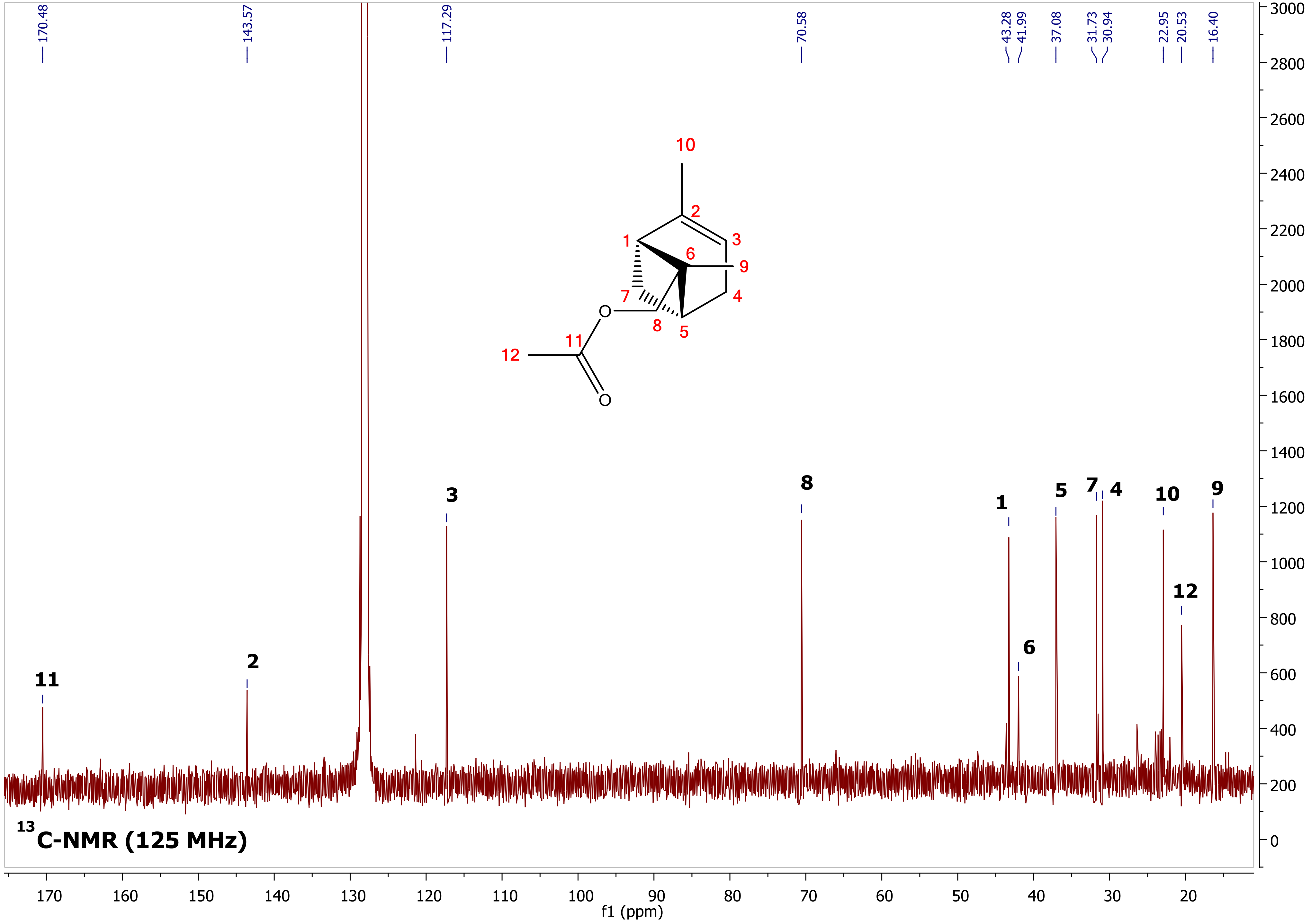

170.48
143.57
117.29
70.58
43.28
41.99
37.08
31.73
30.94
22.95
20.53
16.40
11
2
3
8
1
6
5
7
4
10
12
9
^{13}C-NMR (125 MHz)
f1 (ppm)

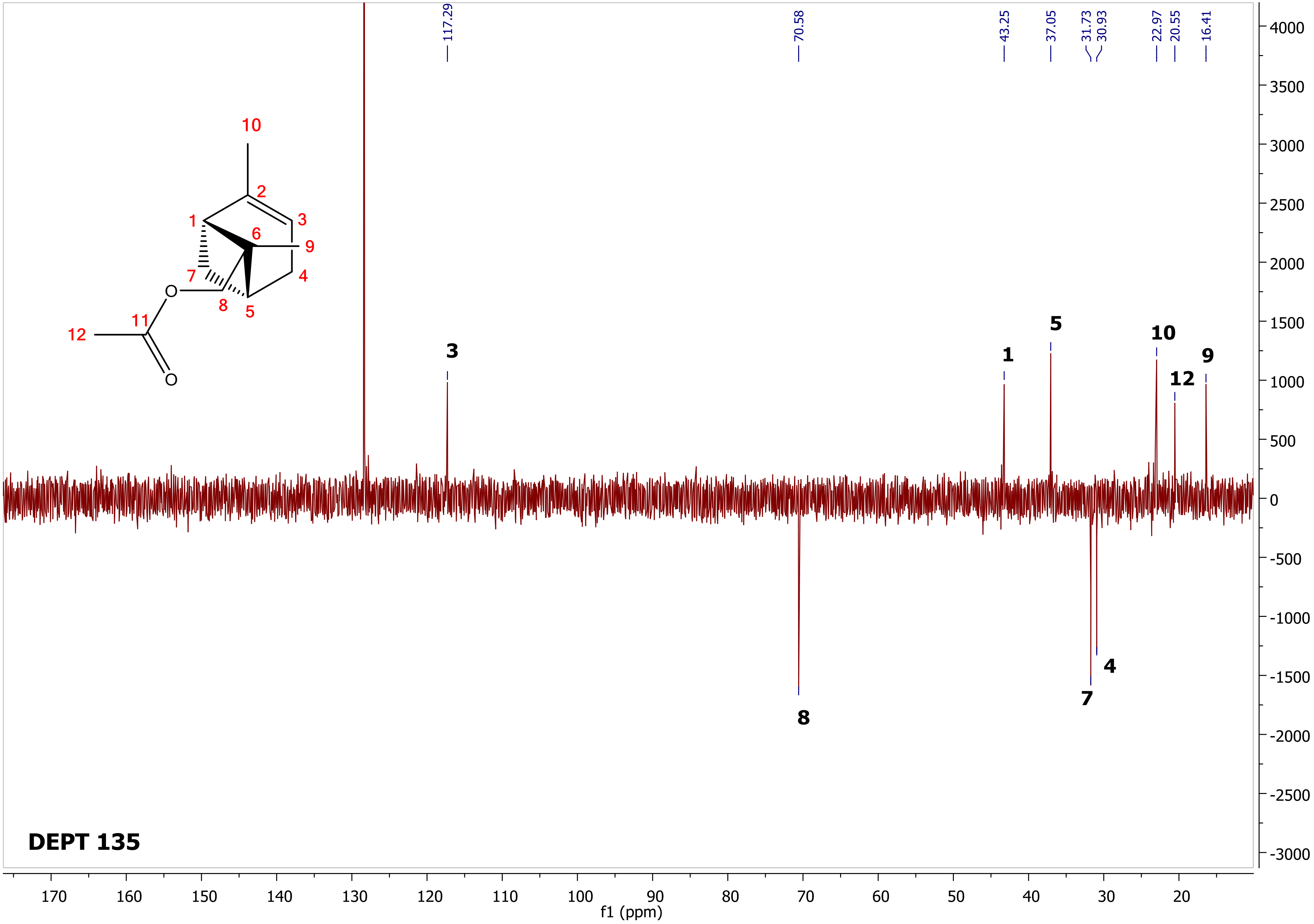
117.29
70.58
43.25
37.05
31.73
30.93
22.97
20.55
16.41
3
8
1
5
7
4
10
12
9
DEPT 135
f1 (ppm)

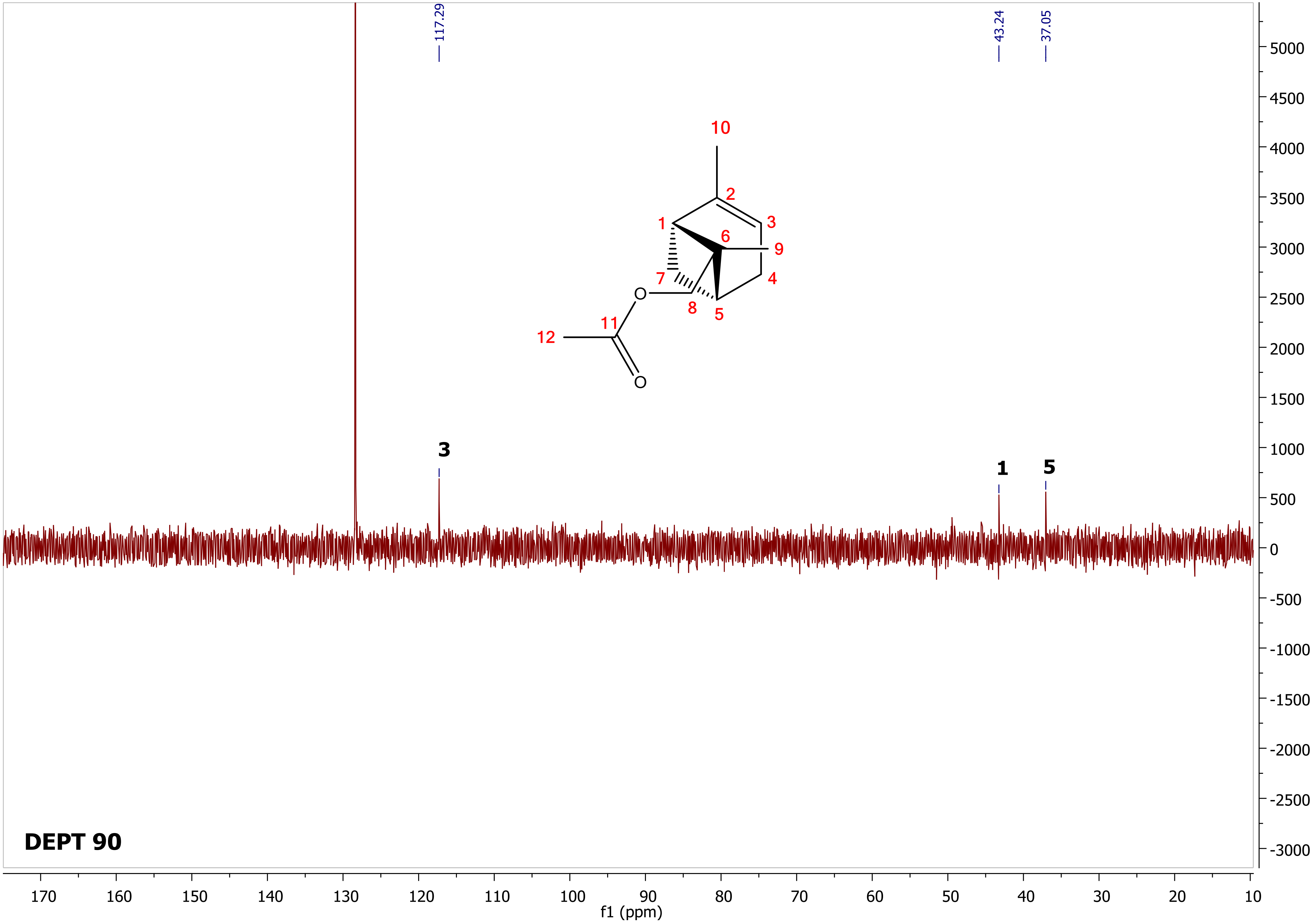

117.29
43.24
37.05
10
2
1
3
6
9
7
4
O
8
5
11
12
O
3
1
5
DEPT 90
5000
4500
4000
3500
3000
2500
2000
1500
1000
500
0
-500
-1000
-1500
-2000
-2500
-3000
170
160
150
140
130
120
110
100
90
80
70
60
50
40
30
20
10
f1 (ppm)

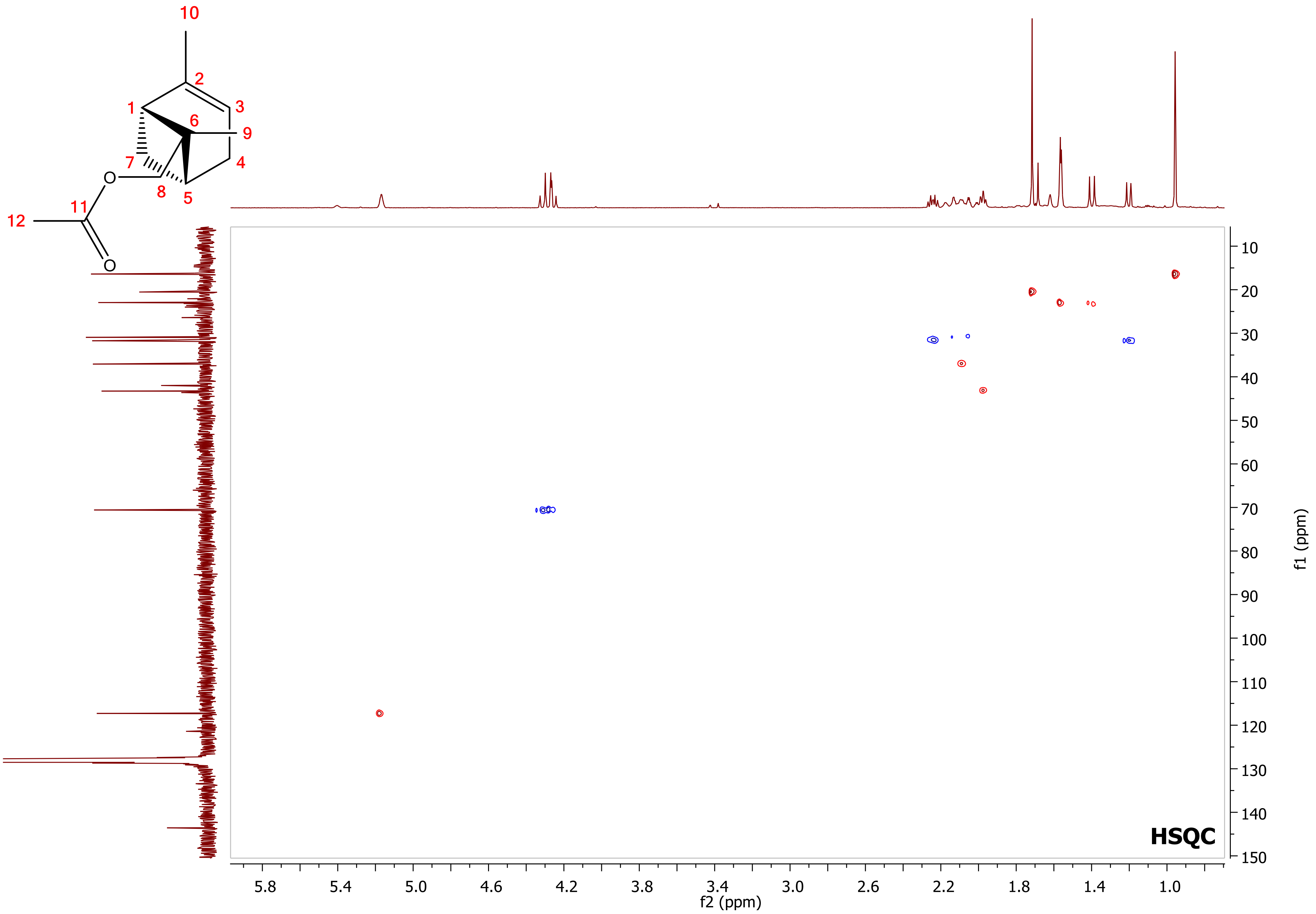

10
2
1
3
6
9
7
4
O
8
5
11
12
O
HSQC
10
20
30
40
50
60
70
80
90
100
110
120
130
140
150
f1 (ppm)
5.8
5.4
5.0
4.6
4.2
3.8
3.4
3.0
2.6
2.2
1.8
1.4
1.0
f2 (ppm)

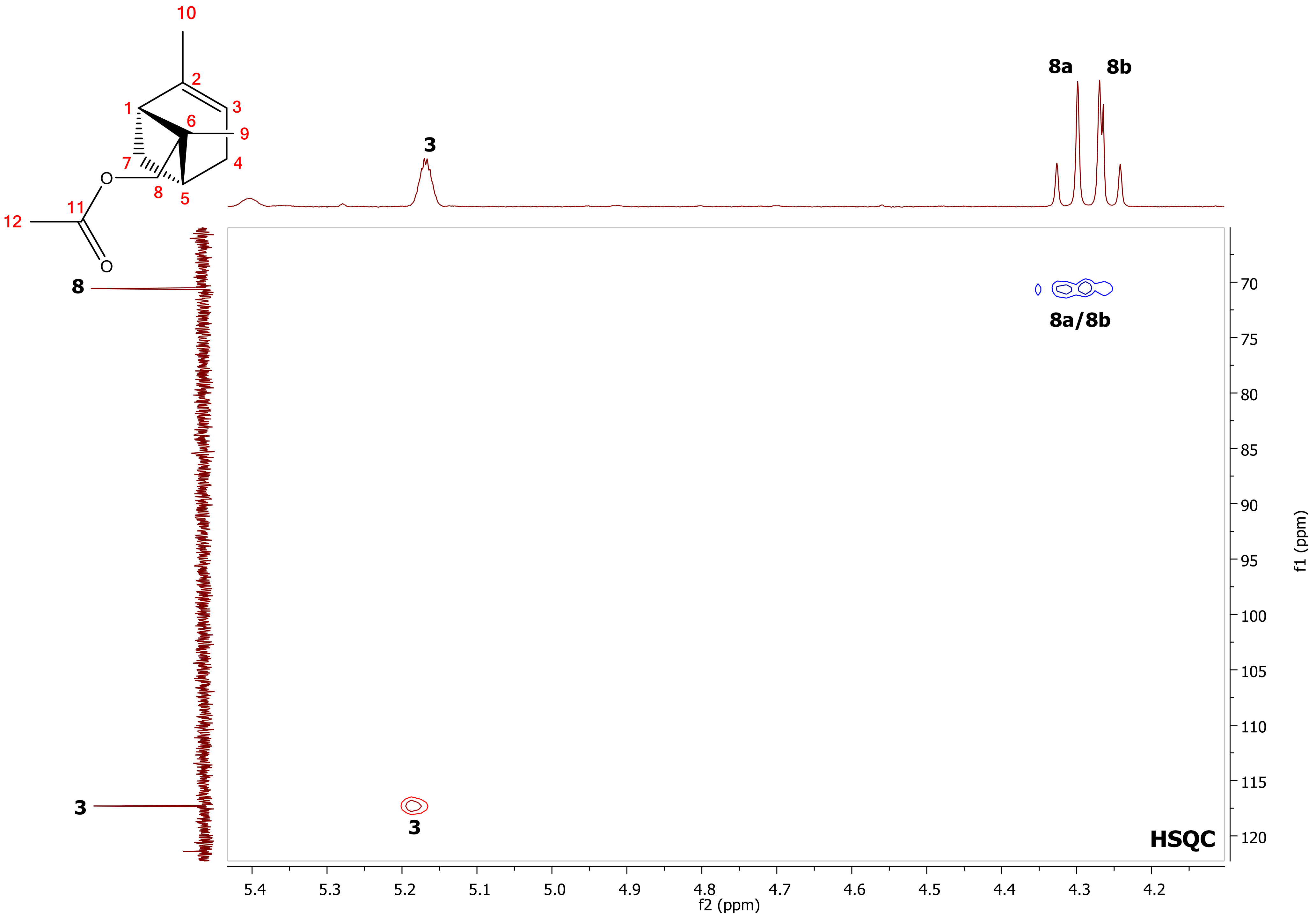

10
2
1
3
6
9
7
4
O
8
5
11
12
O
8a
8b
3
8
8a/8b
3
3
HSQC
f1 (ppm)
f2 (ppm)

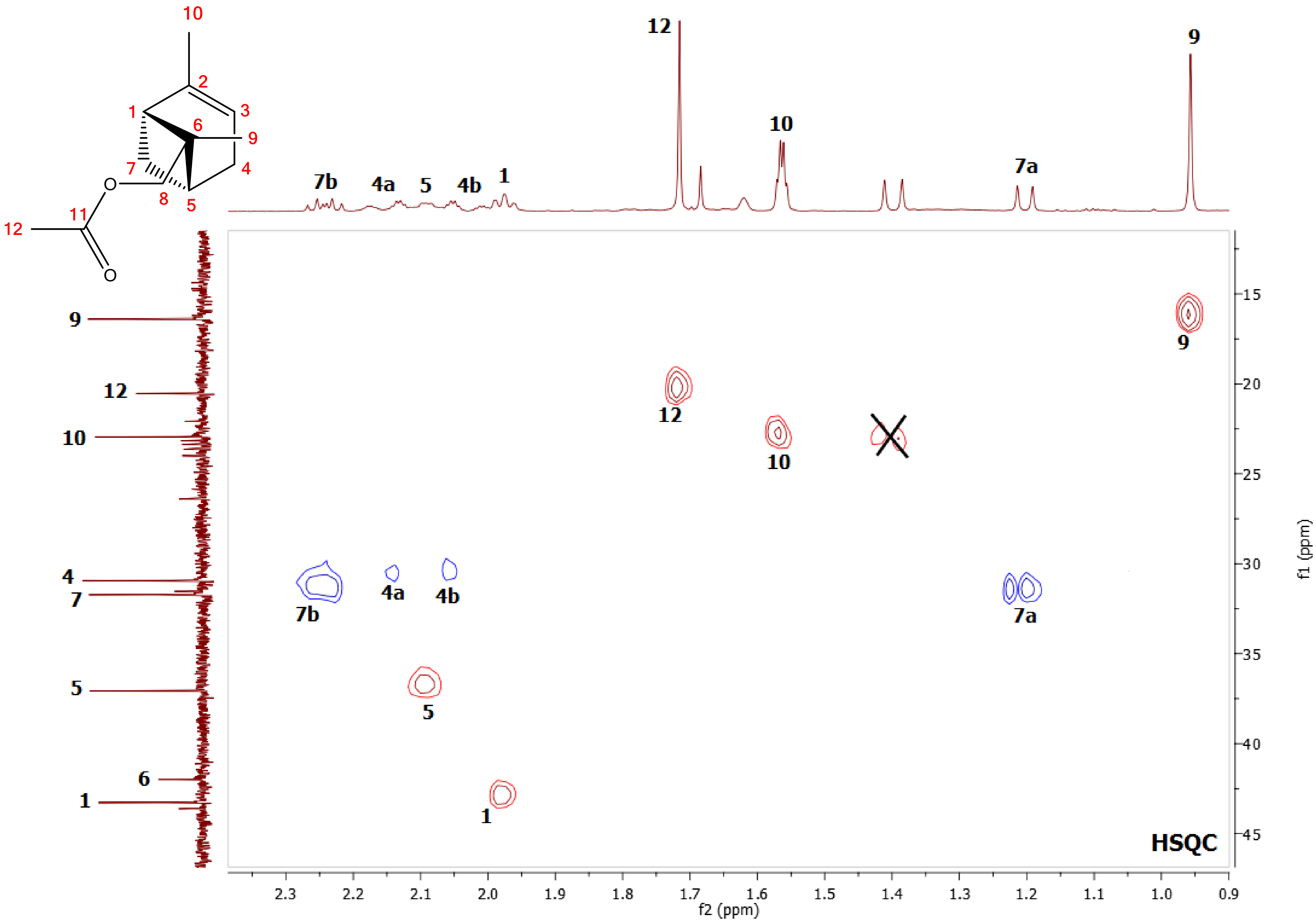

10
2
3
1
6
9
7
4
O
8
5
11
12
O
7b
4a
5
4b
1
12
10
7a
9
HSQC
f2 (ppm)
f1 (ppm)

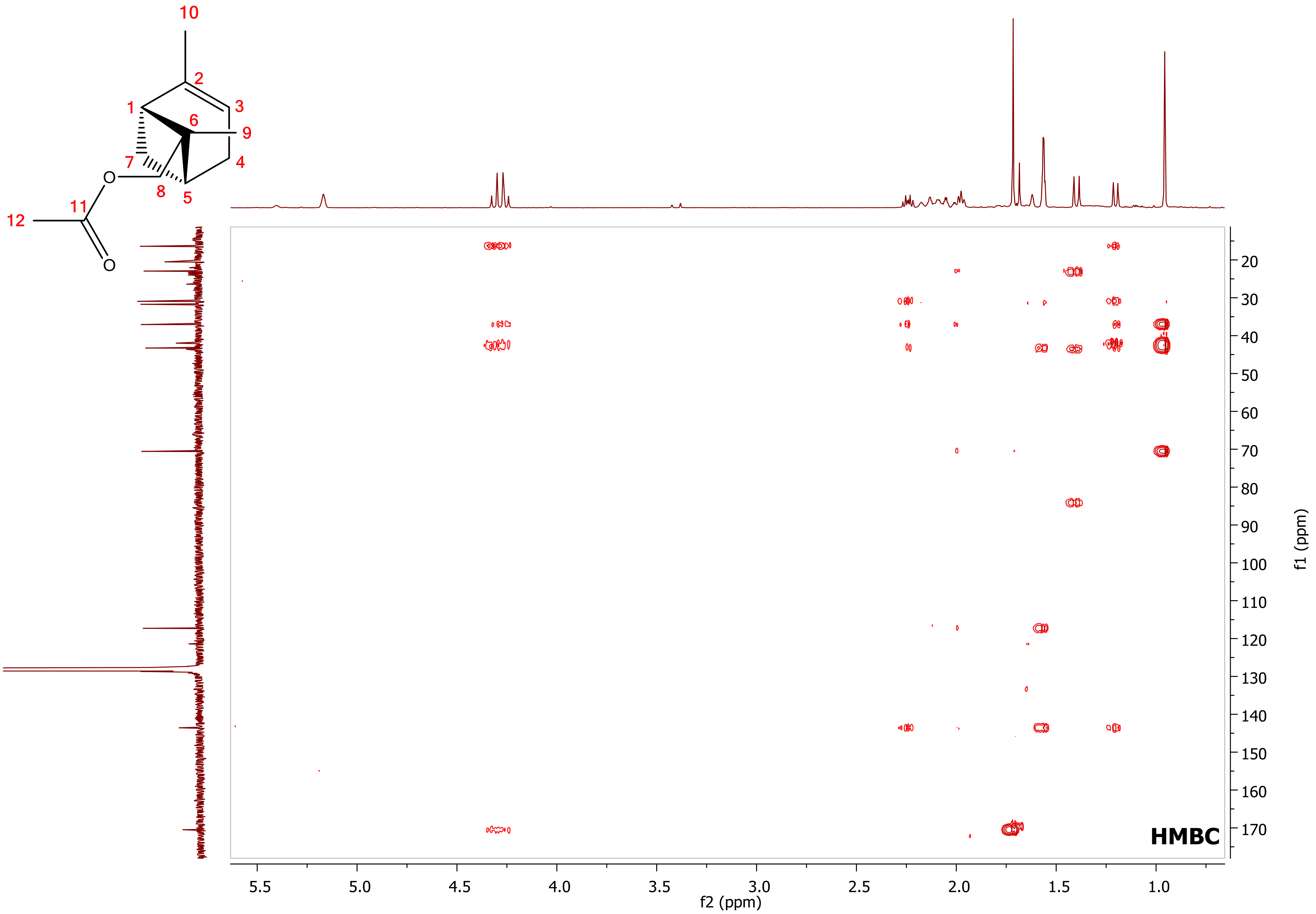

10
2
1
3
6
9
7
4
O
8
5
11
12
O
HMBC
5.5
5.0
4.5
4.0
3.5
3.0
2.5
2.0
1.5
1.0
f2 (ppm)
20
30
40
50
60
70
80
90
100
110
120
130
140
150
160
170
f1 (ppm)

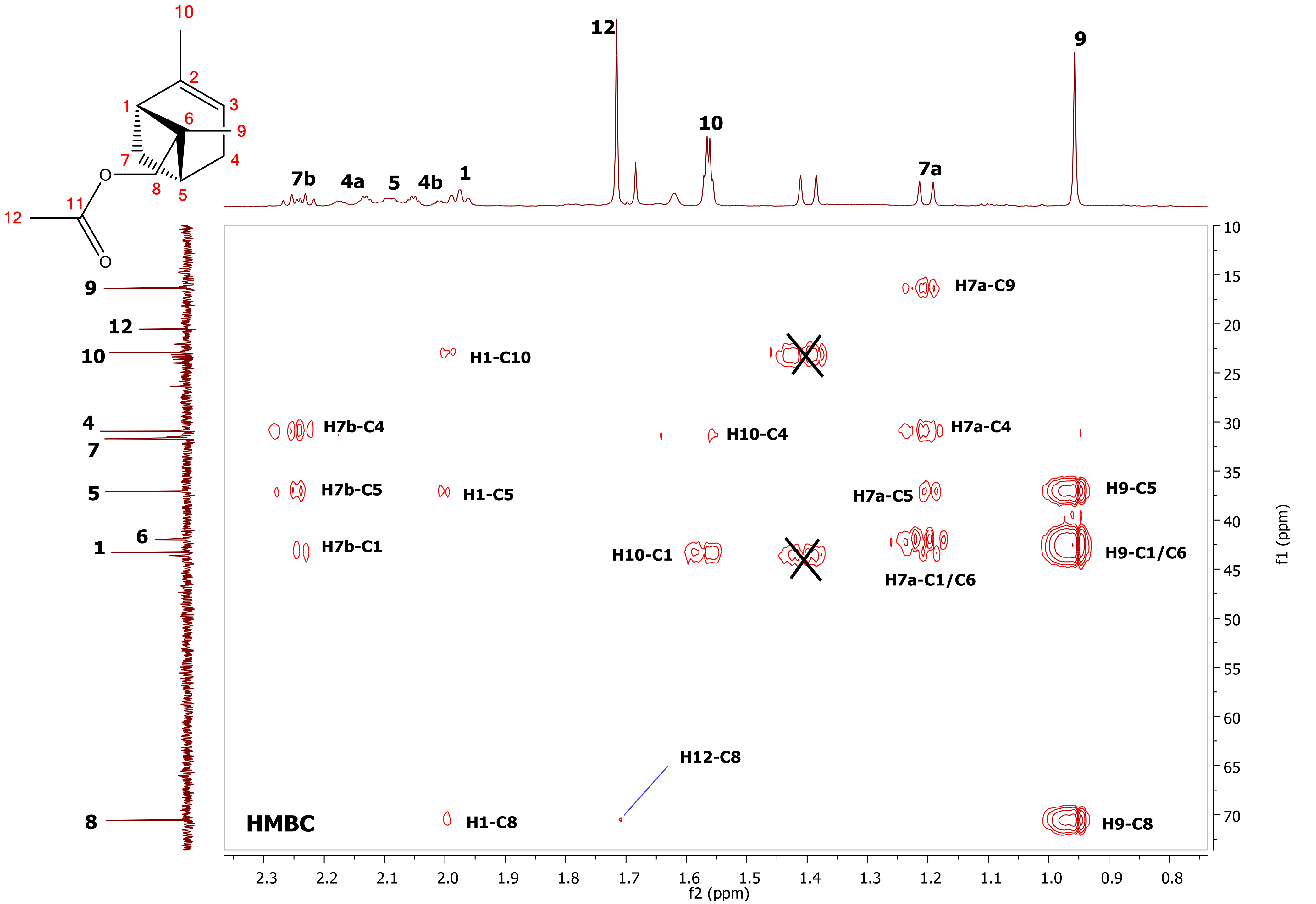

10
2
3
1
6
9
7
4
O
8
5
11
12
O
12
10
7b
4a
5
4b
1
7a
9
9
12
10
4
7
5
6
1
8
H7a-C9
H1-C10
H7b-C4
H10-C4
H7a-C4
H7b-C5
H1-C5
H7a-C5
H9-C5
H7b-C1
H10-C1
H7a-C1/C6
H9-C1/C6
H12-C8
HMBC
H1-C8
H9-C8
f2 (ppm)
f1 (ppm)

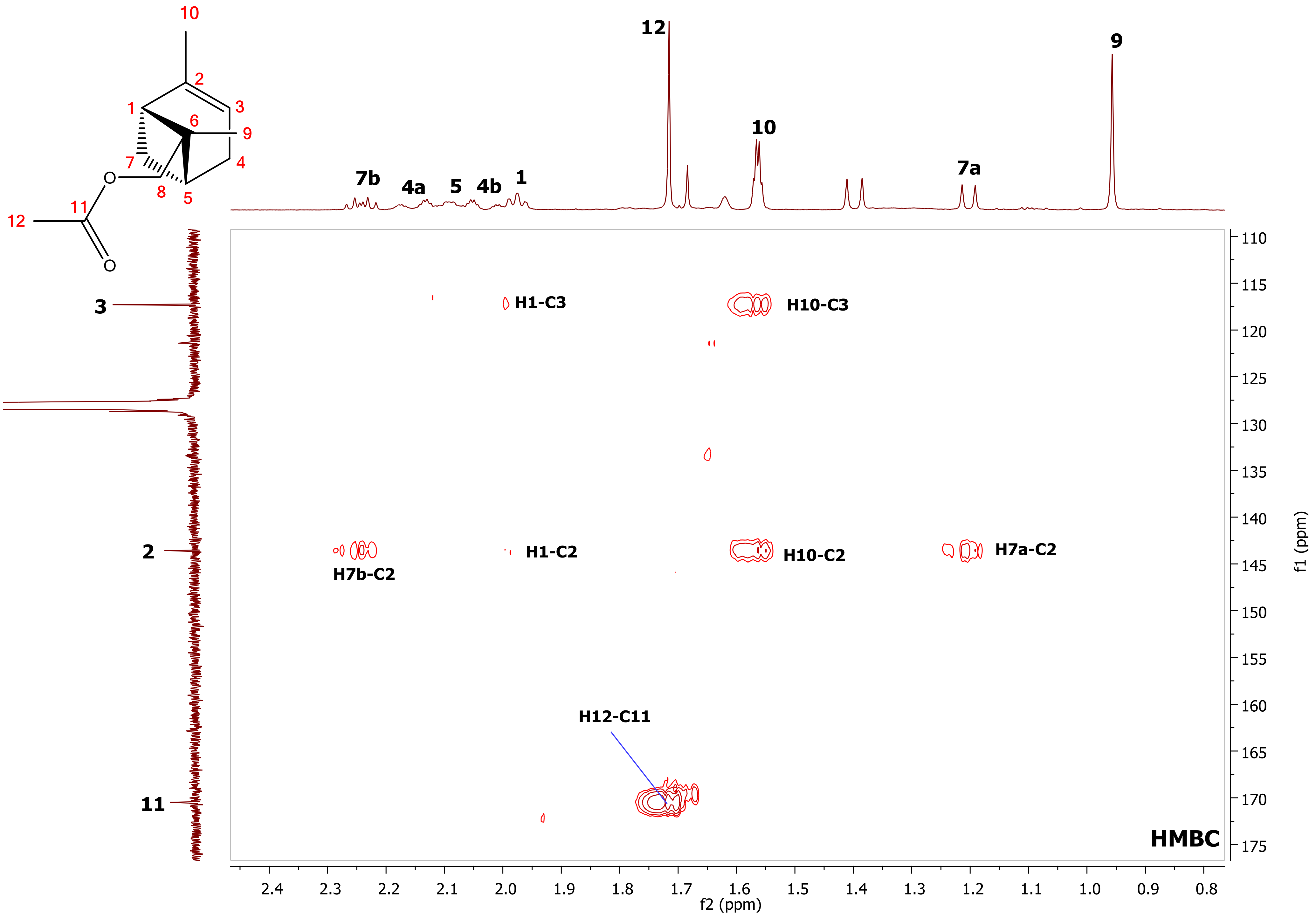
10
2
3
1
6
9
7
4
O
8
5
11
12
O
7b
4a
5
4b
1
12
10
7a
9
3
2
11
H1-C3
H10-C3
H1-C2
H10-C2
H7a-C2
H7b-C2
H12-C11
HMBC
f2 (ppm)
f1 (ppm)

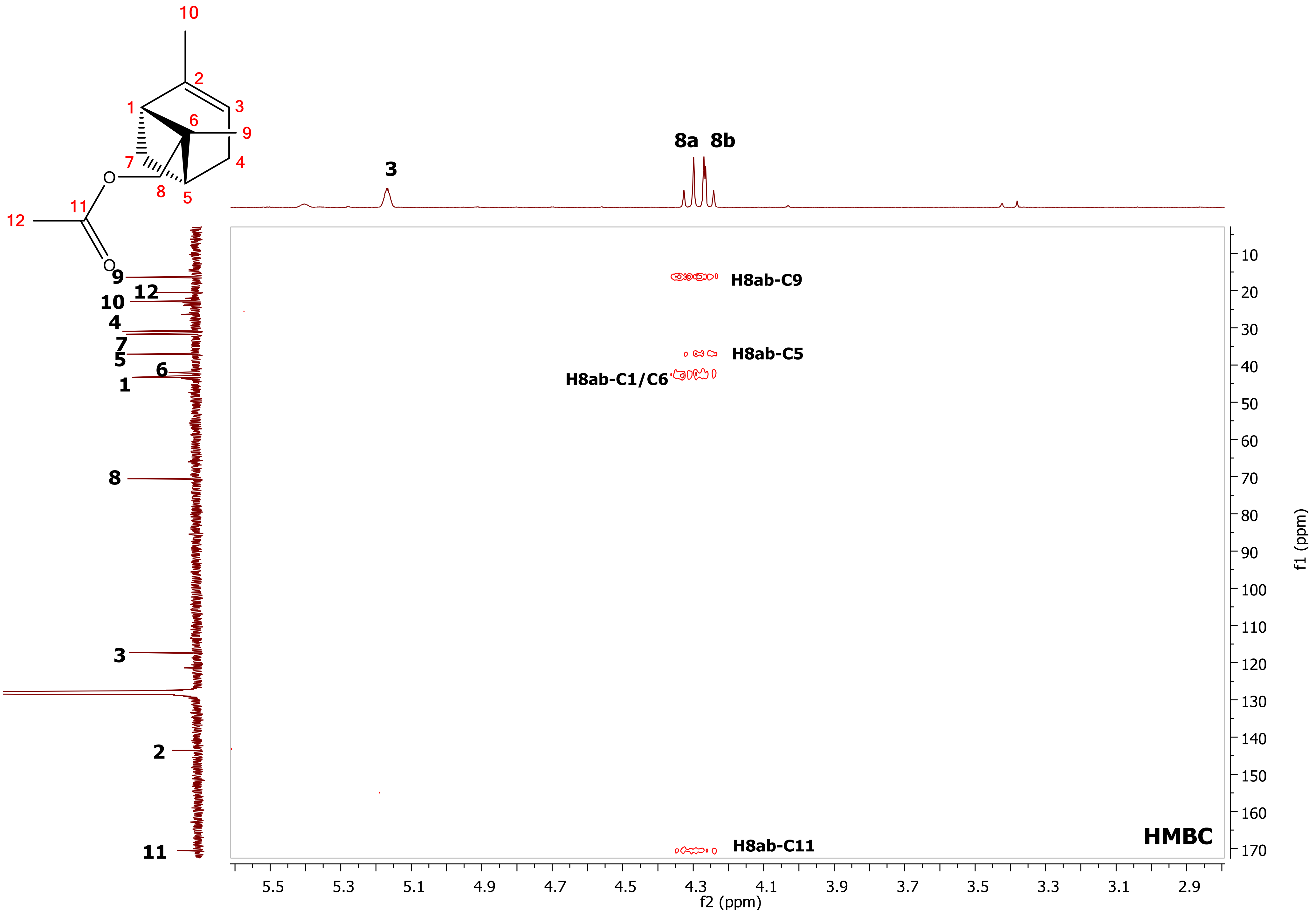

10
2
1
3
6
9
7
4
O
8
5
11
12
O
3
8a 8b
9
12
10
4
7
5
6
1
8
3
2
11
H8ab-C9
H8ab-C5
H8ab-C1/C6
H8ab-C11
HMBC
f1 (ppm)
f2 (ppm)

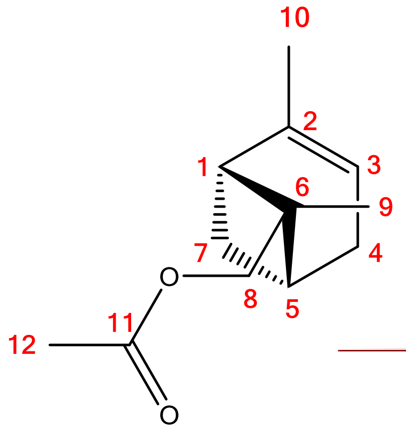
10
2
3
1
6
9
7
4
O
8
5
11
12
O

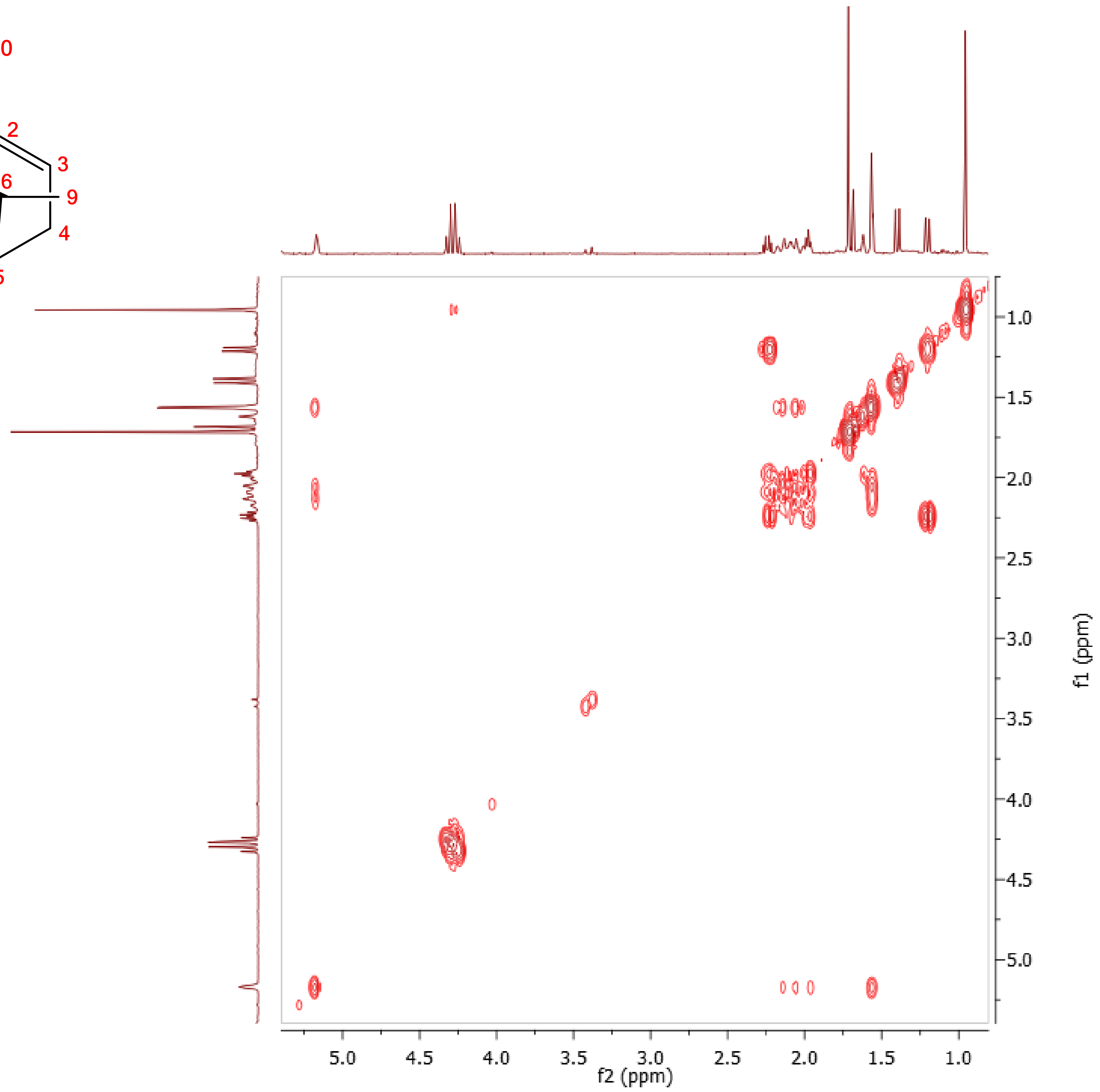
f2 (ppm)
f1 (ppm)

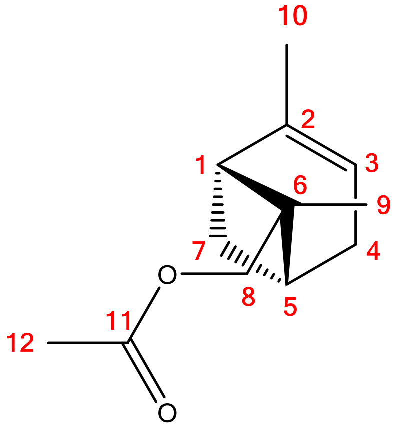
10
2
3
1
6
9
7
4
O
8
5
11
12
O

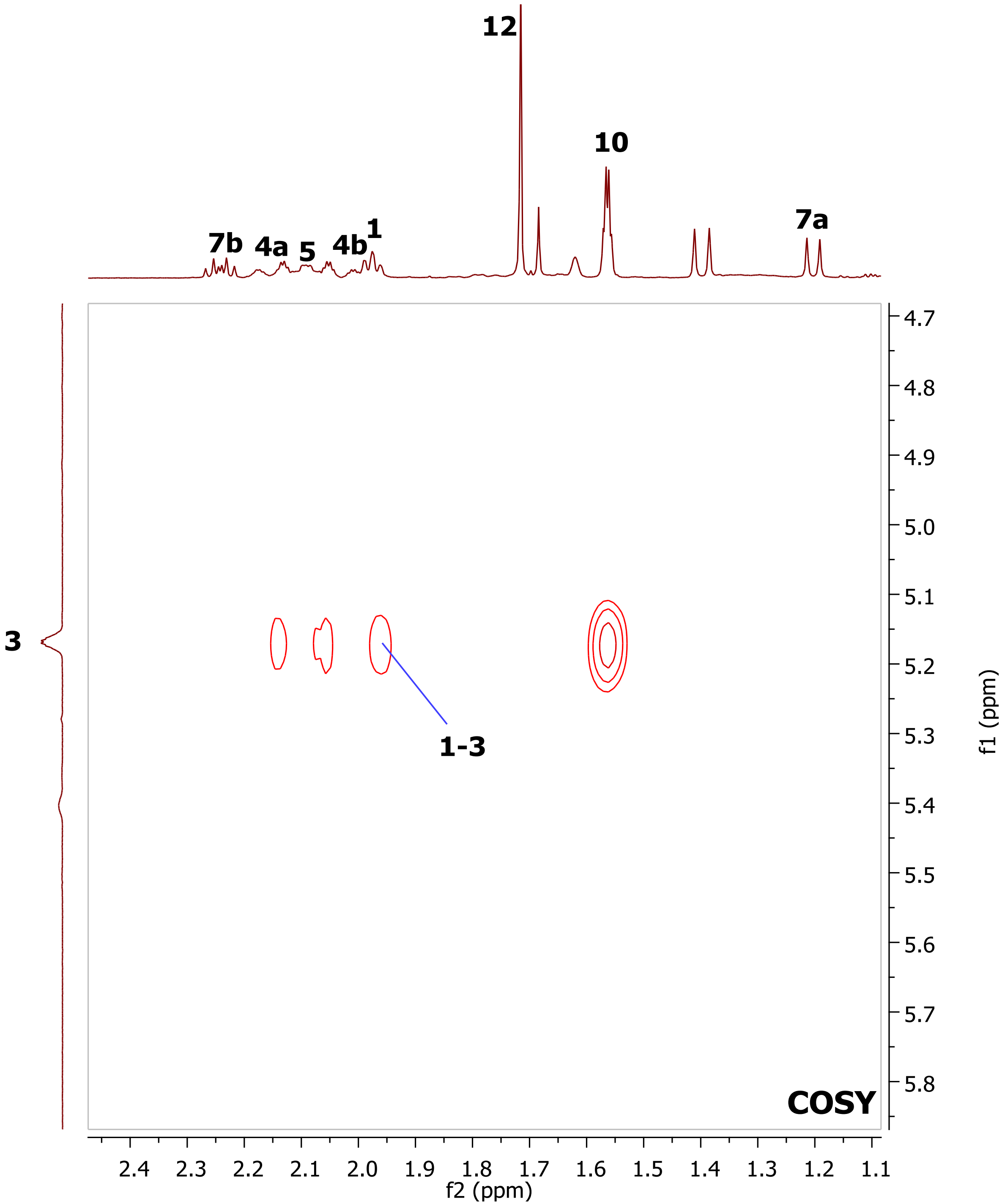
12
10
7b
4a
5
4b
1
7a
3
1-3
COSY
2.4
2.3
2.2
2.1
2.0
1.9
1.8
1.7
1.6
1.5
1.4
1.3
1.2
1.1
f2 (ppm)
4.7
4.8
4.9
5.0
5.1
5.2
5.3
5.4
5.5
5.6
5.7
5.8
f1 (ppm)

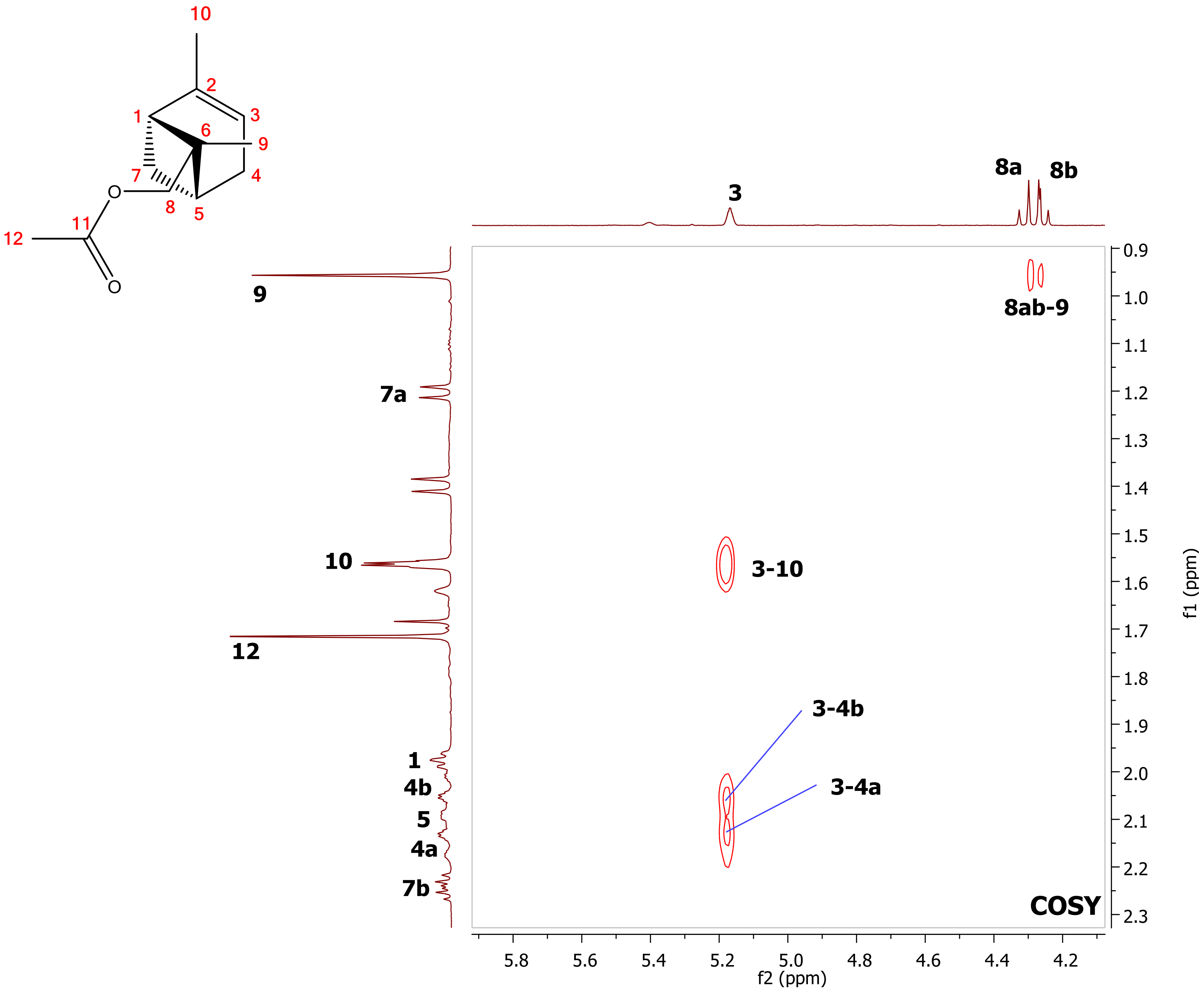

10
2
3
1
6
9
7
4
O
8
5
11
12
O
8a
8b
3
9
7a
10
12
1
4b
5
4a
7b
8ab-9
3-10
3-4b
3-4a
COSY
f1 (ppm)
f2 (ppm)

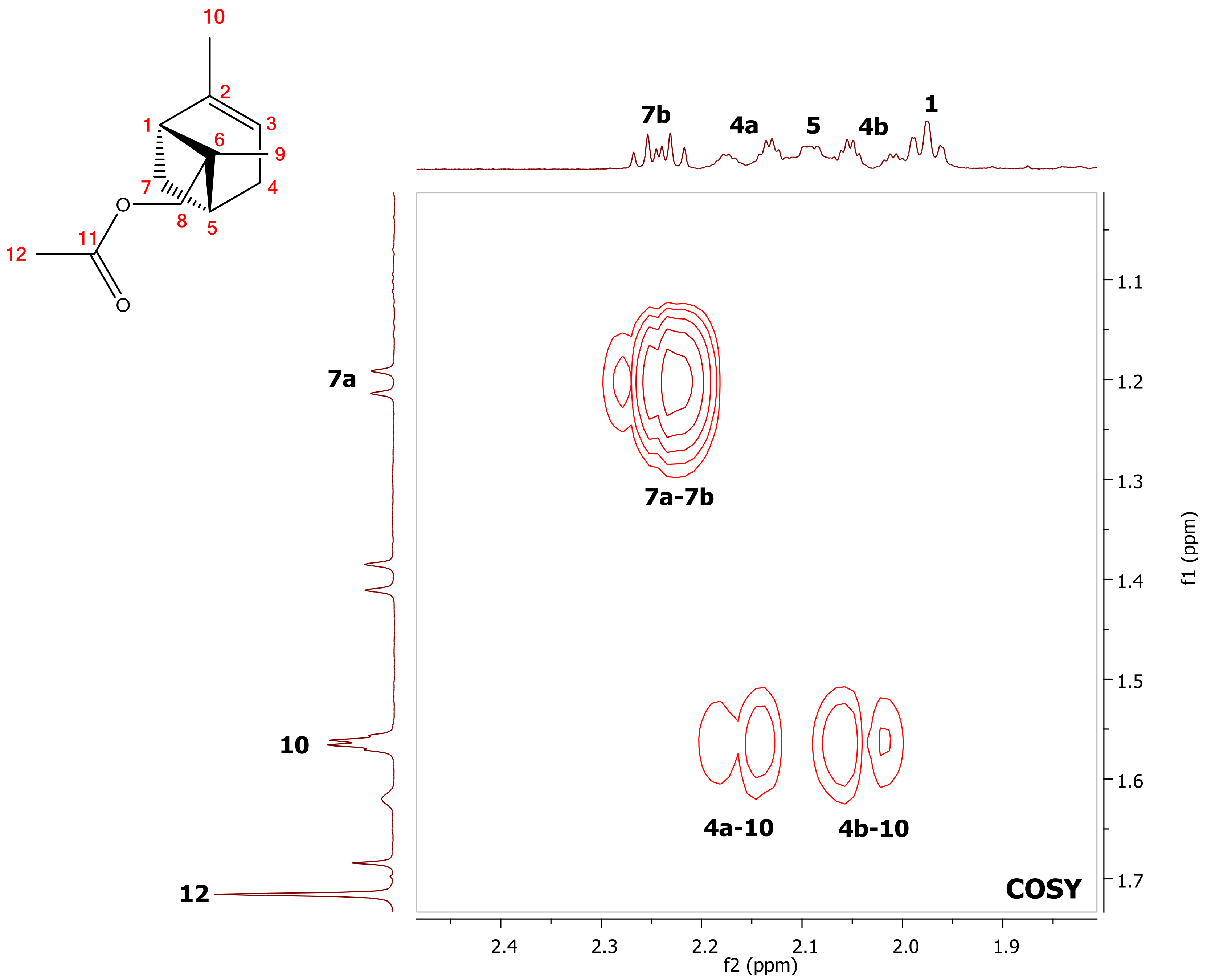
10
2
1
3
6
9
7
4
O
8
5
11
12
O
7b
4a
5
4b
1
7a
7a-7b
10
4a-10
4b-10
12
COSY
2.4
2.3
2.2
2.1
2.0
1.9
f2 (ppm)
1.1
1.2
1.3
1.4
1.5
1.6
1.7
f1 (ppm)

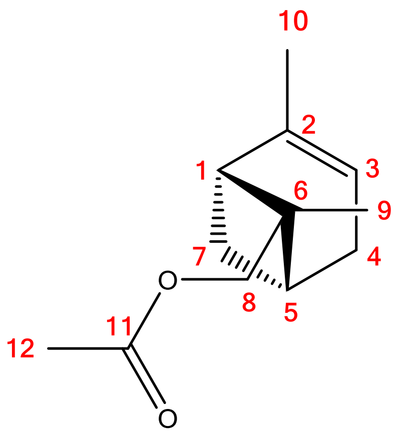

10
2
1
3
6
9
7
4
O
8
5
11
12
O

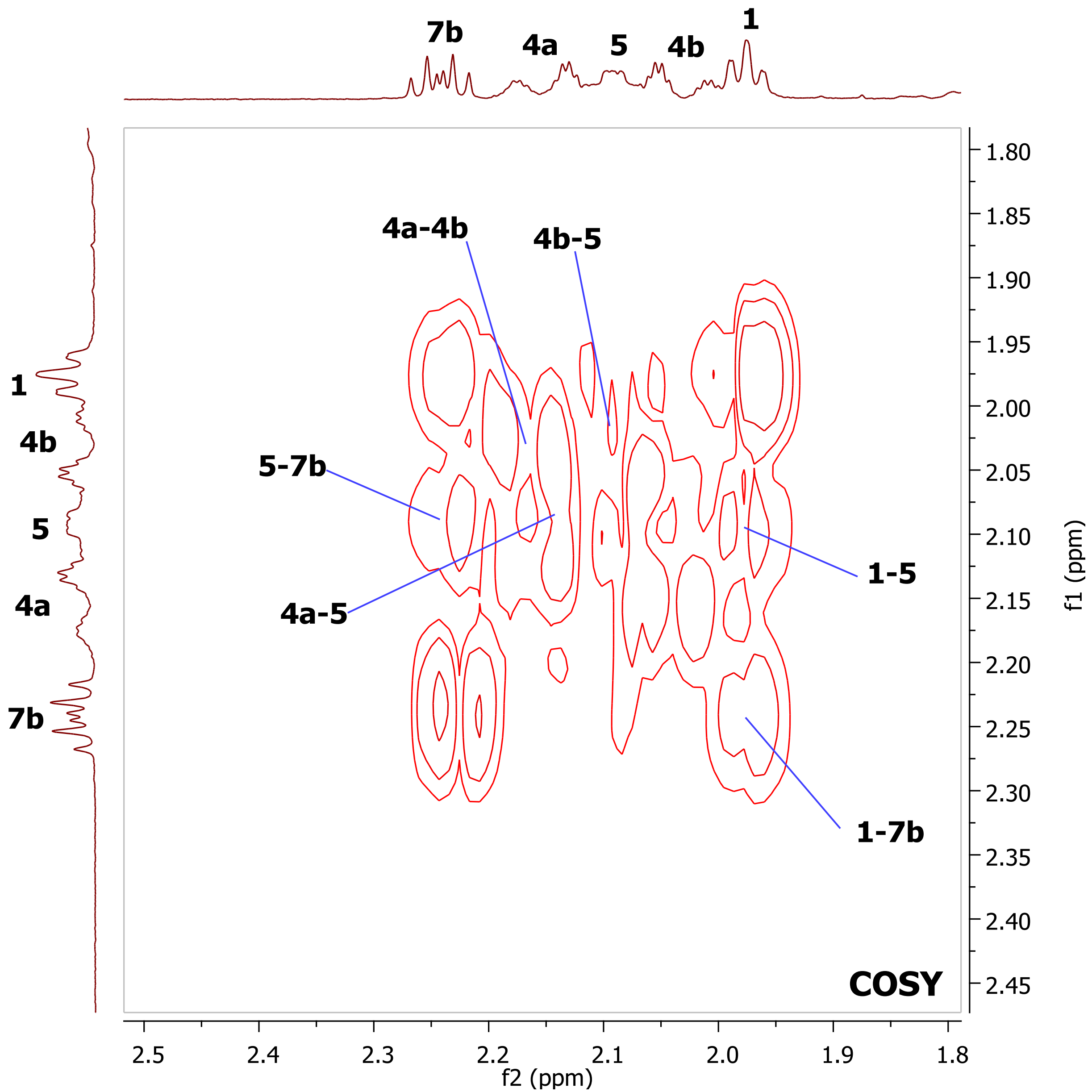

7b
4a
5
4b
1
4a-4b
4b-5
5-7b
4a-5
1-5
1-7b
COSY
f1 (ppm)
f2 (ppm)

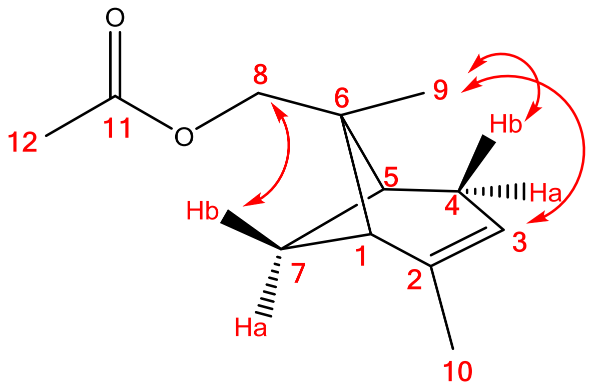

Key NOEs for compound **B**

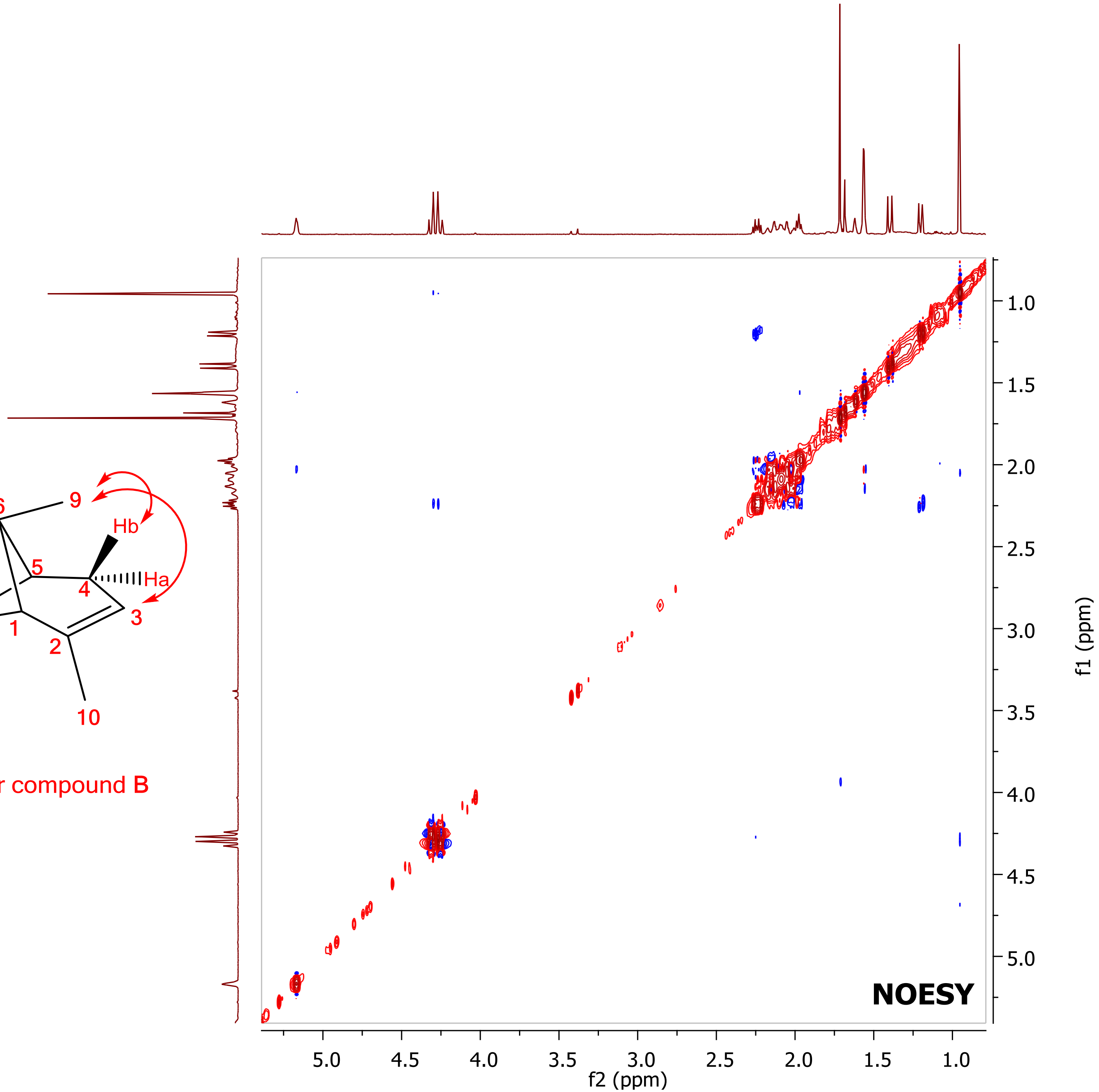

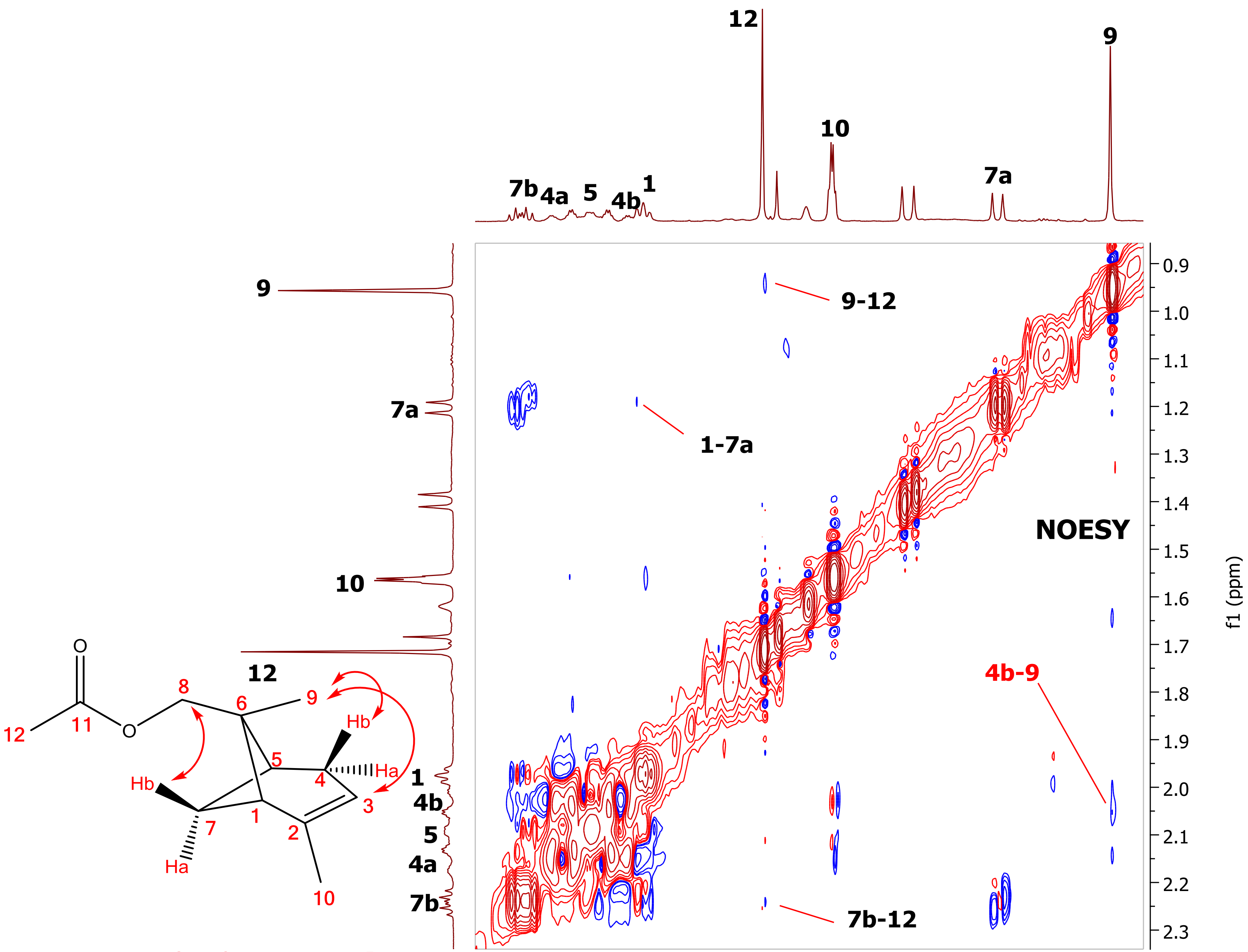

Key NOEs for compound B

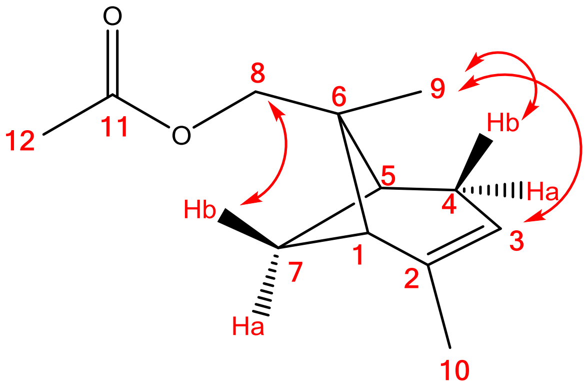

Key NOEs for compound B

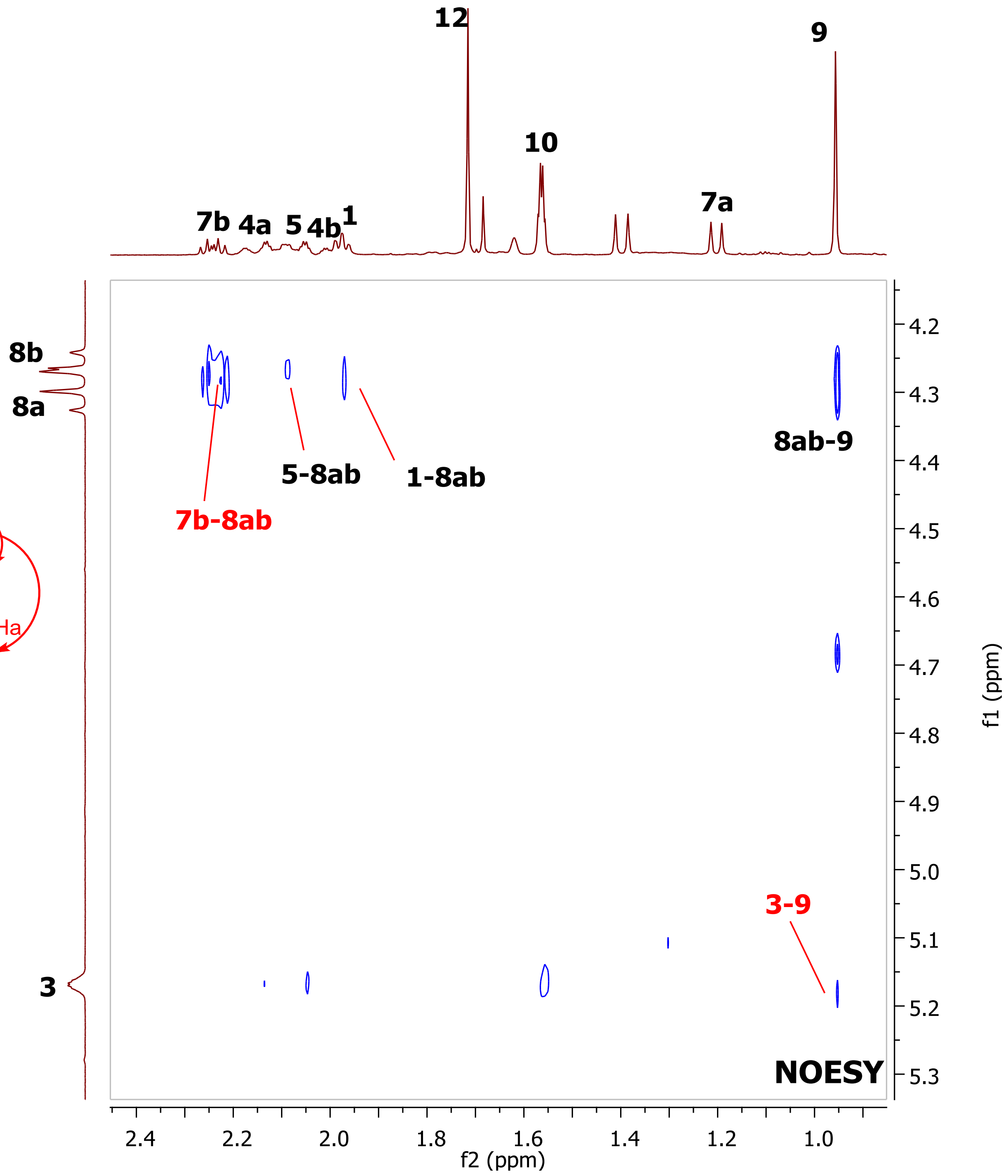

1D- and 2D-NMR data for Pin-2-en-8-al (C)

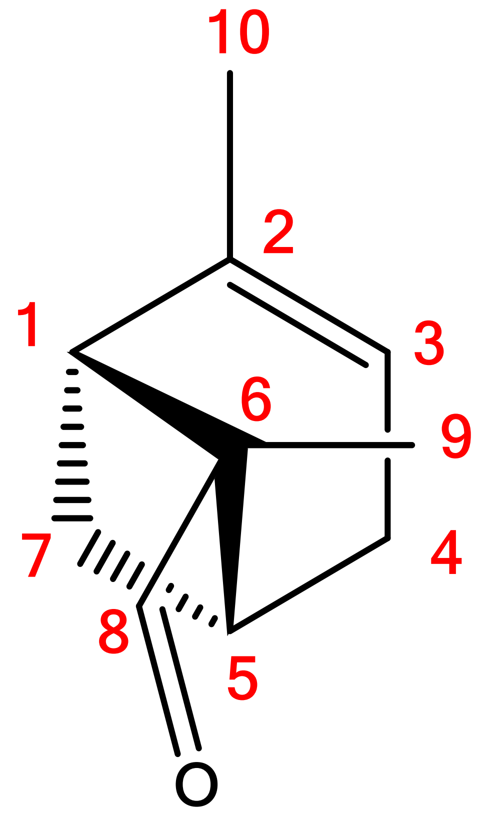

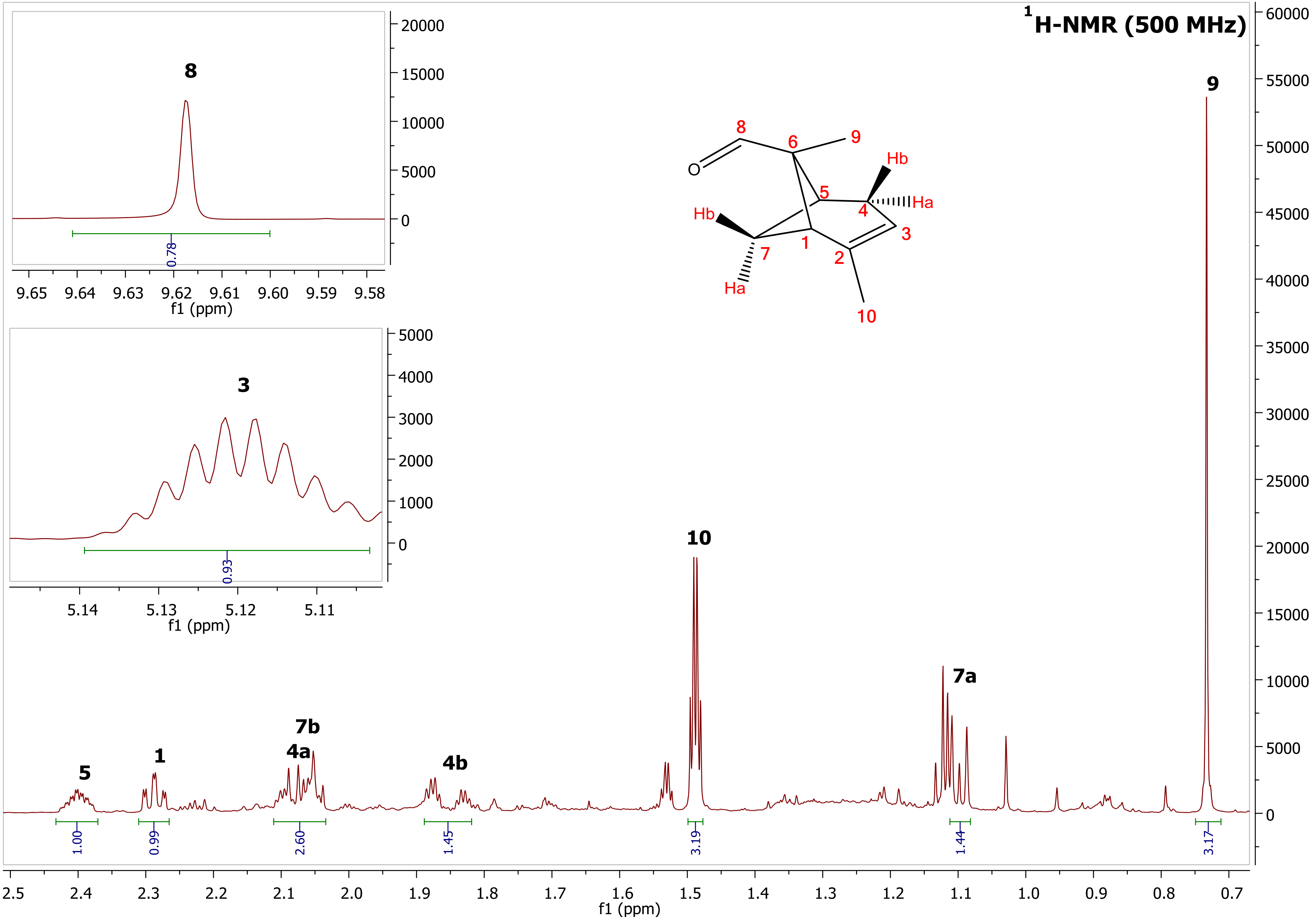

1H-NMR (500 MHz)
8
9
10
7a
7b
4a
4b
5
1
3
O
Hb
Ha
f1 (ppm)

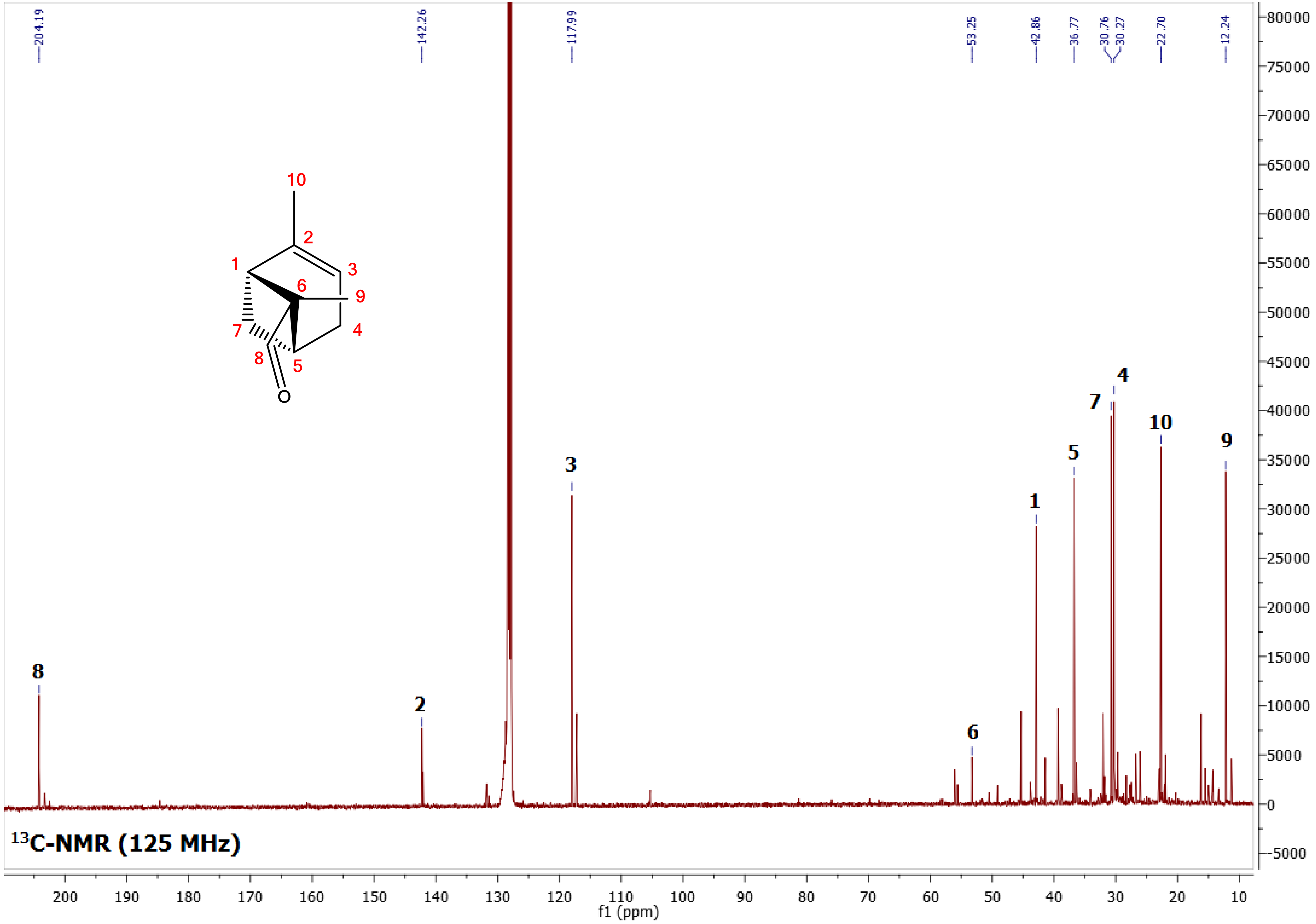

204.19
142.26
117.99
53.25
42.86
36.77
30.76
30.27
22.70
12.24
10
2
1
3
6
9
7
4
8
5
O
8
2
3
6
1
5
7
4
10
9
^{13}C-NMR (125 MHz)
f1 (ppm)

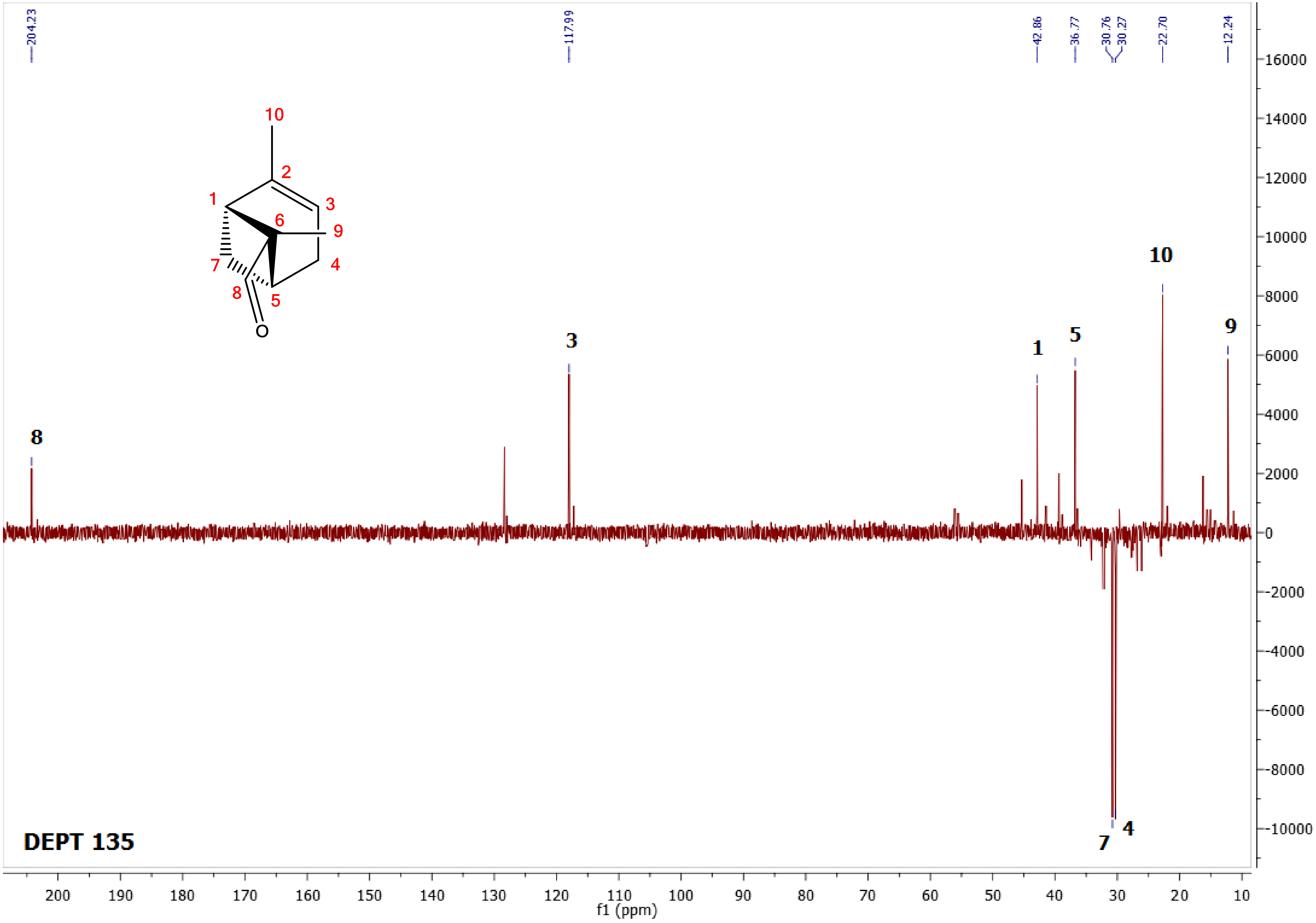
204.23
117.99
42.86
36.77
30.76
30.27
22.70
12.24
10
2
1
3
6
9
7
4
8
5
O
8
3
1
5
10
9
7
4
DEPT 135
f1 (ppm)

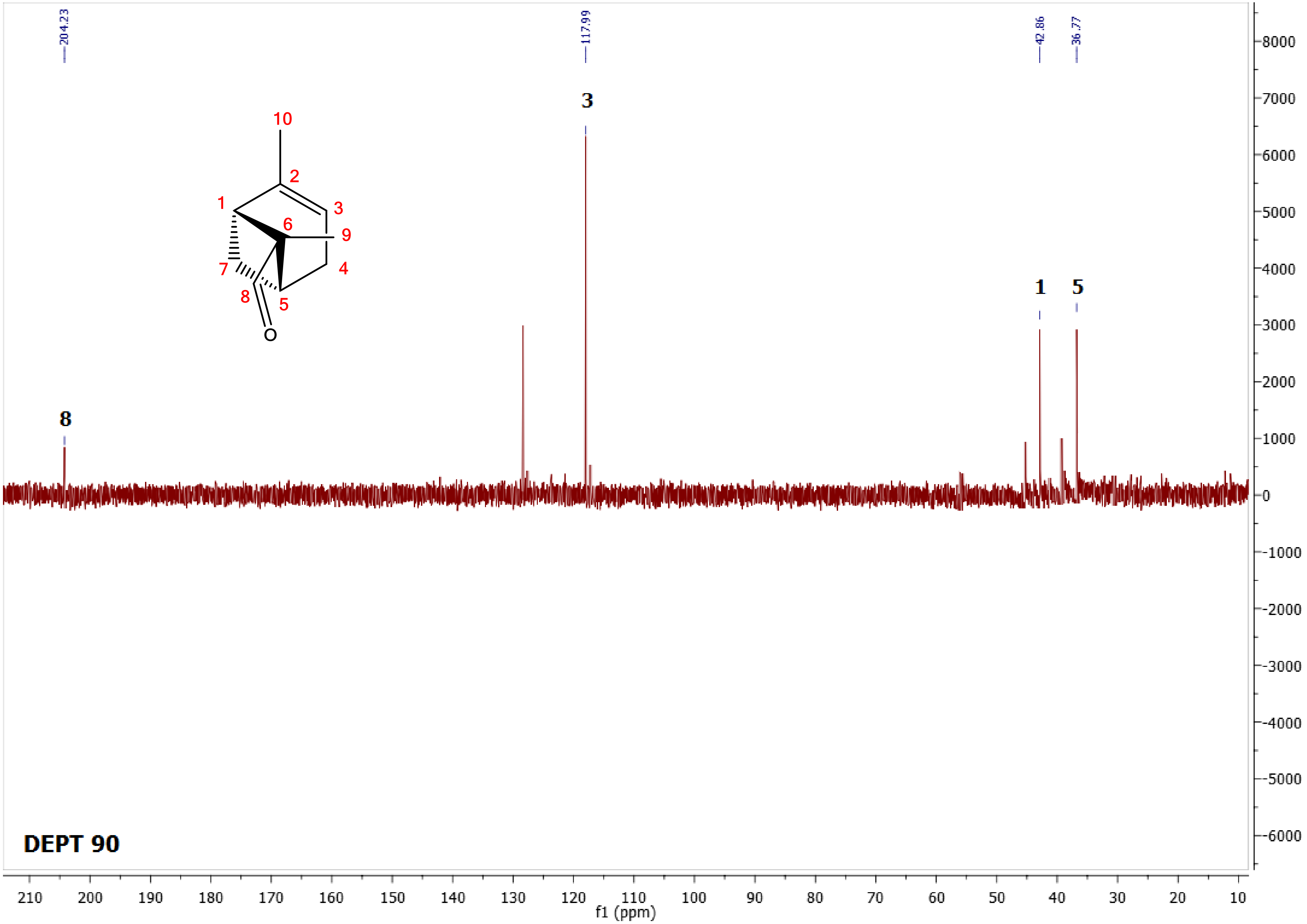

204.23
117.99
42.86
36.77
10
2
1
3
6
9
7
4
8
5
O
3
8
1
5
DEPT 90
210 200 190 180 170 160 150 140 130 120 110 100 90 80 70 60 50 40 30 20 10
f1 (ppm)
8000 7000 6000 5000 4000 3000 2000 1000 0 -1000 -2000 -3000 -4000 -5000 -6000

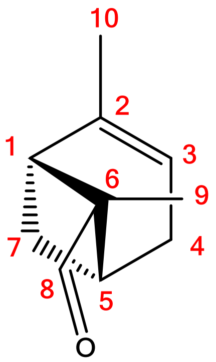

10
2
1
3
6
9
7
4
8
5
O

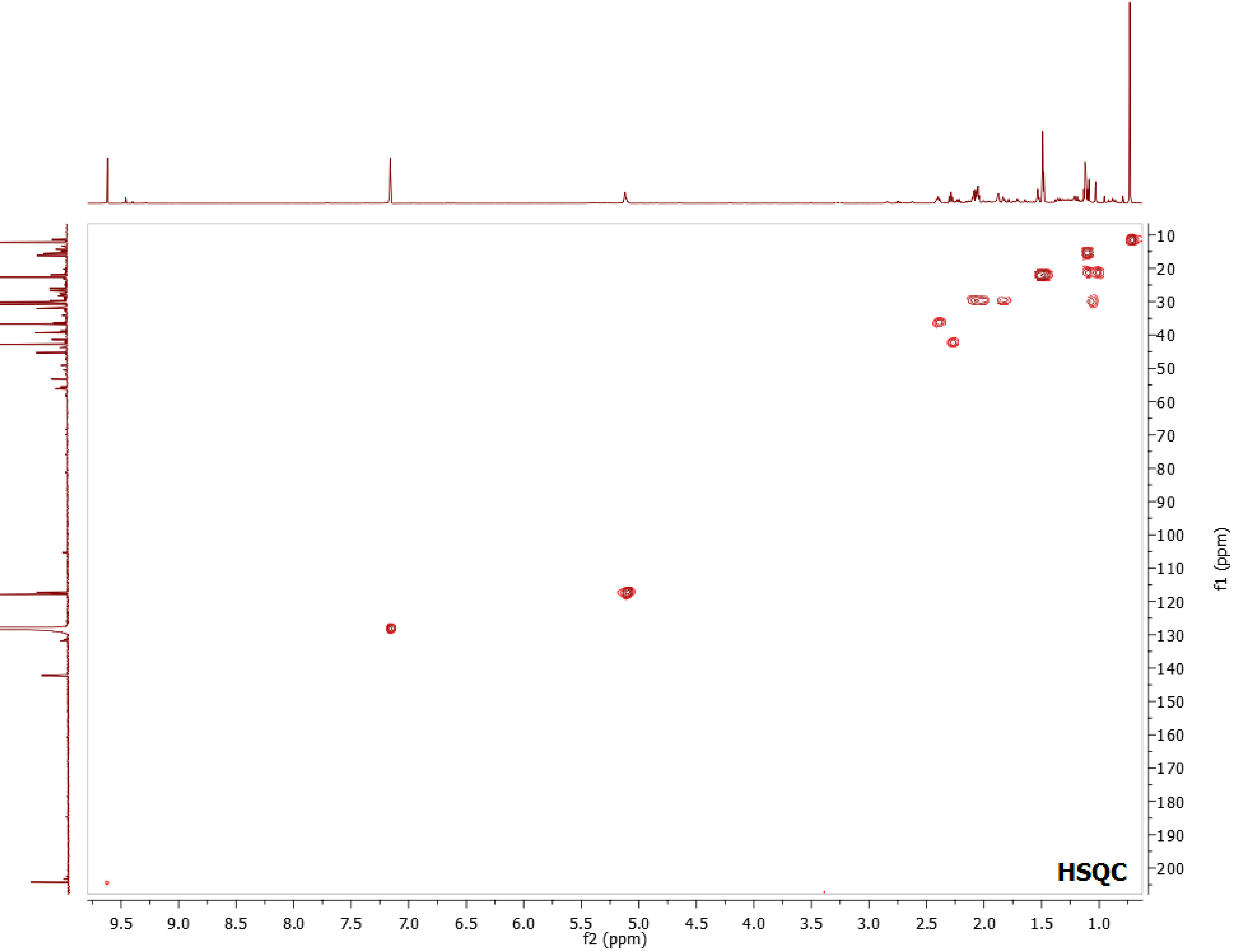

HSQC
f2 (ppm)
f1 (ppm)
9.5
9.0
8.5
8.0
7.5
7.0
6.5
6.0
5.5
5.0
4.5
4.0
3.5
3.0
2.5
2.0
1.5
1.0
10
20
30
40
50
60
70
80
90
100
110
120
130
140
150
160
170
180
190
200

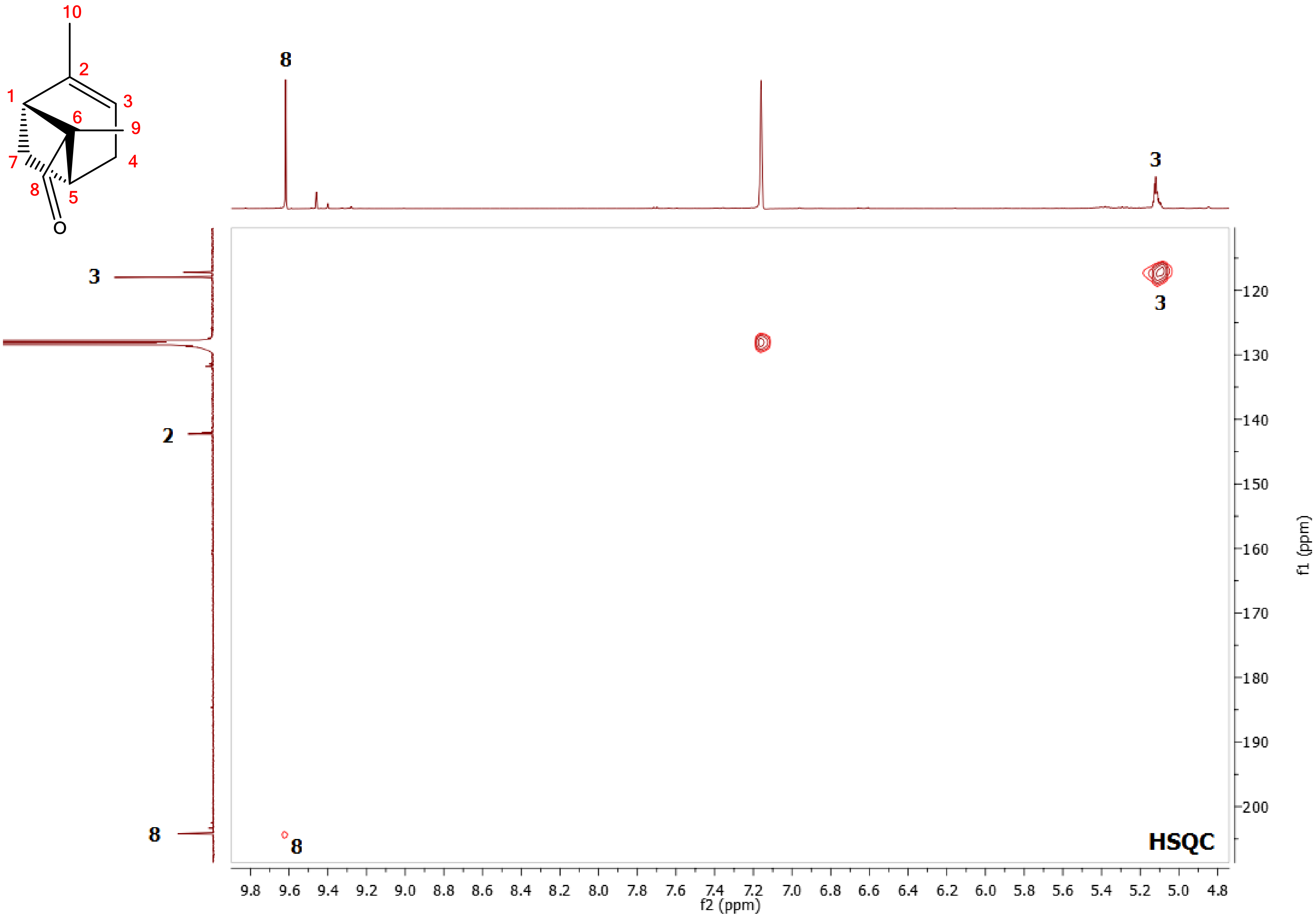
10
2
3
1
6
9
7
4
8
5
O
8
3
3
3
2
8
8
HSQC
f2 (ppm)
f1 (ppm)

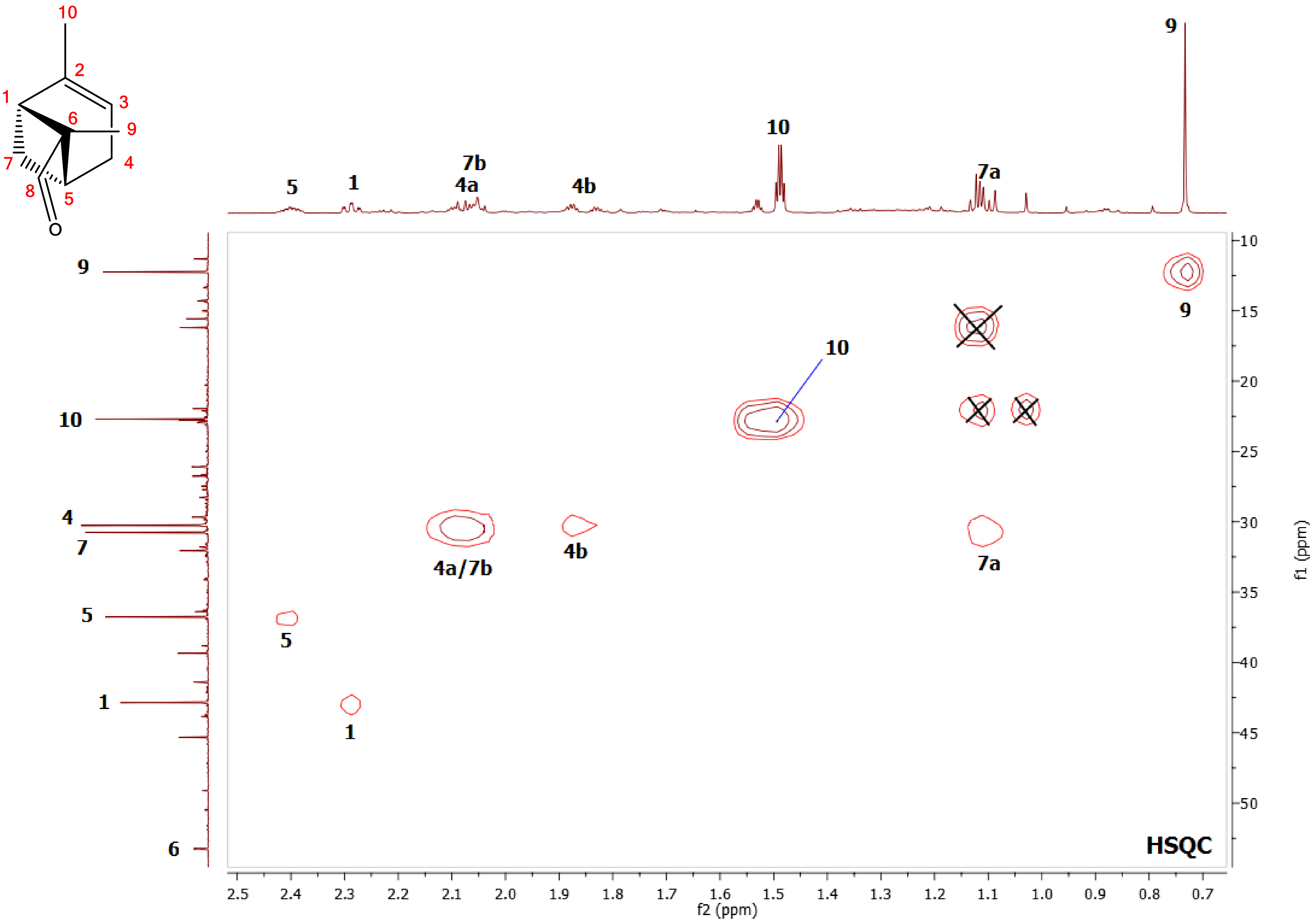
10
2
1
3
6
9
7
4
8
5
O
9
10
7a
5
1
7b
4a
4b
4
7
6
4a/7b
HSQC
f2 (ppm)
f1 (ppm)

1D- and 2D-NMR data for Methyl pin-2-en-8-oate (D)

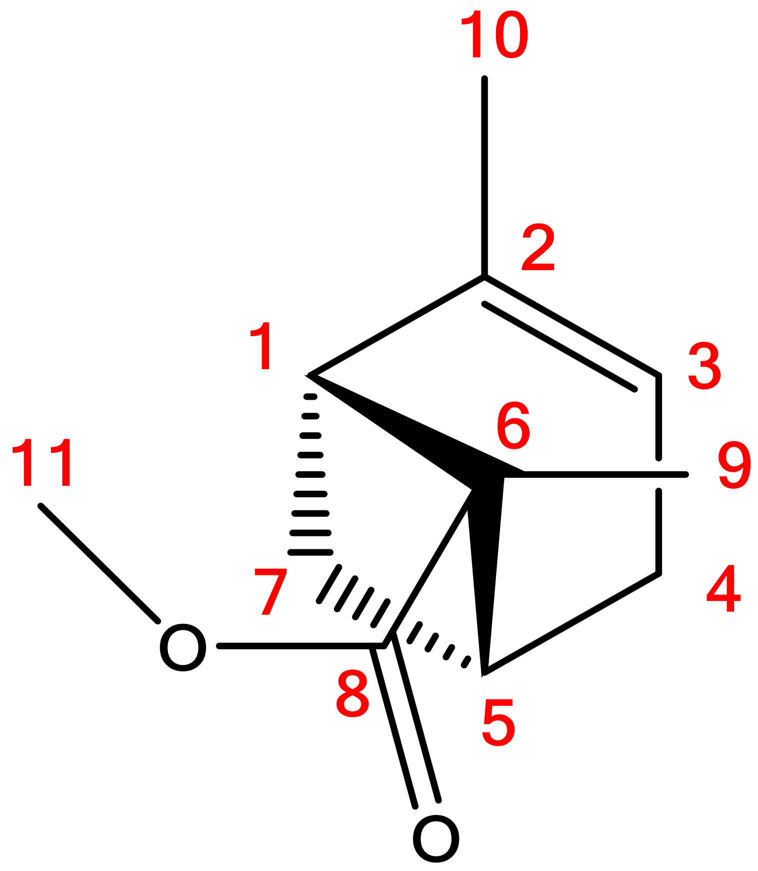

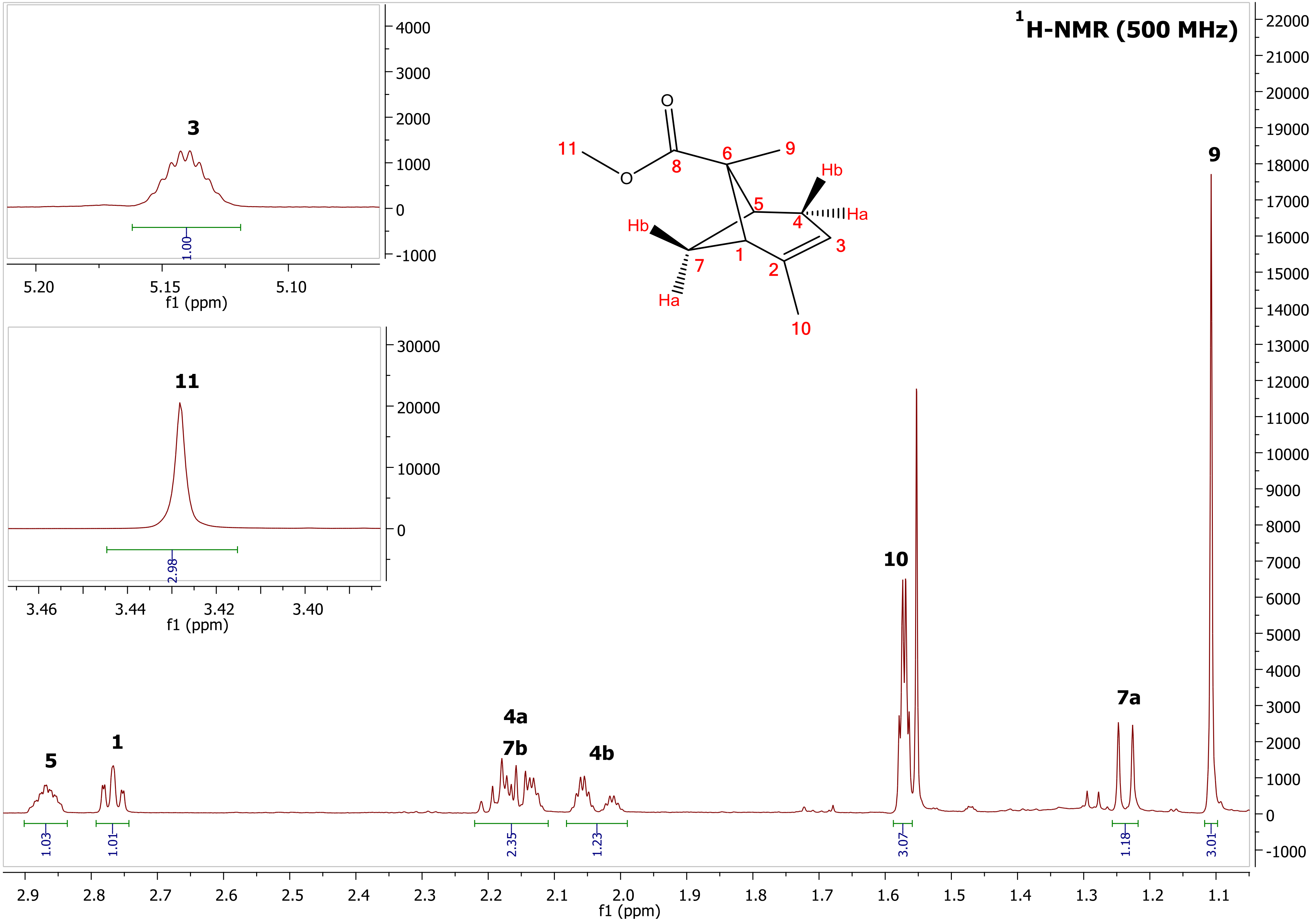

1H-NMR (500 MHz)
O
11
O
8
6
9
5
Hb
4
Ha
Hb
3
7
1
2
Ha
10
3
1.00
5.20
5.15
5.10
f1 (ppm)
11
2.98
3.46
3.44
3.42
3.40
f1 (ppm)
9
10
7a
4a
7b
4b
5
1
1.03
1.01
2.35
1.23
3.07
1.18
3.01
2.9
2.8
2.7
2.6
2.5
2.4
2.3
2.2
2.1
2.0
1.9
1.8
1.7
1.6
1.5
1.4
1.3
1.2
1.1
f1 (ppm)

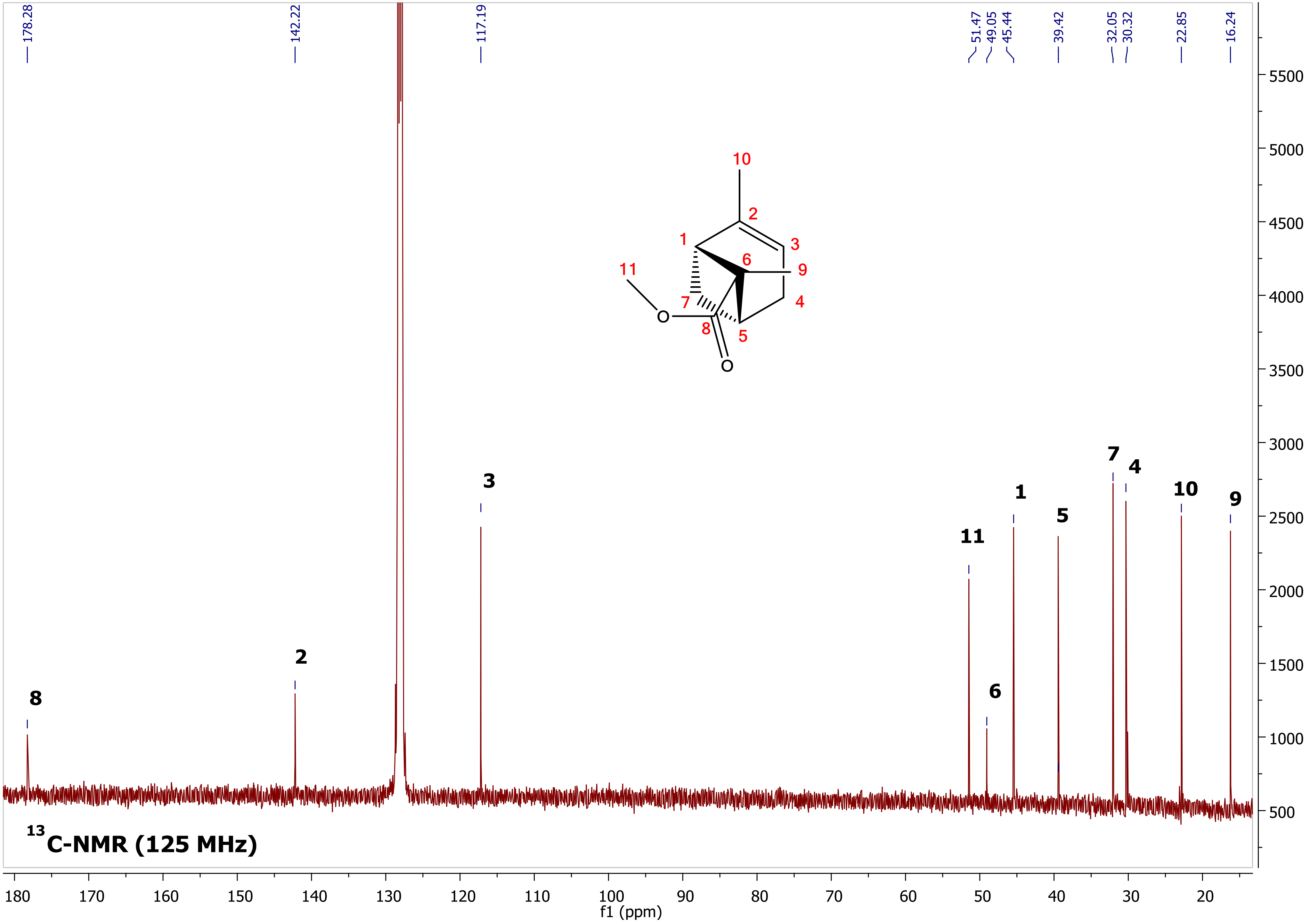

178.28
142.22
117.19
51.47
49.05
45.44
39.42
32.05
30.32
22.85
16.24
10
2
1
3
11
6
9
7
4
8
5
O
O
8
2
3
11
6
1
5
7
4
10
9
^{13}C-NMR (125 MHz)
f1 (ppm)

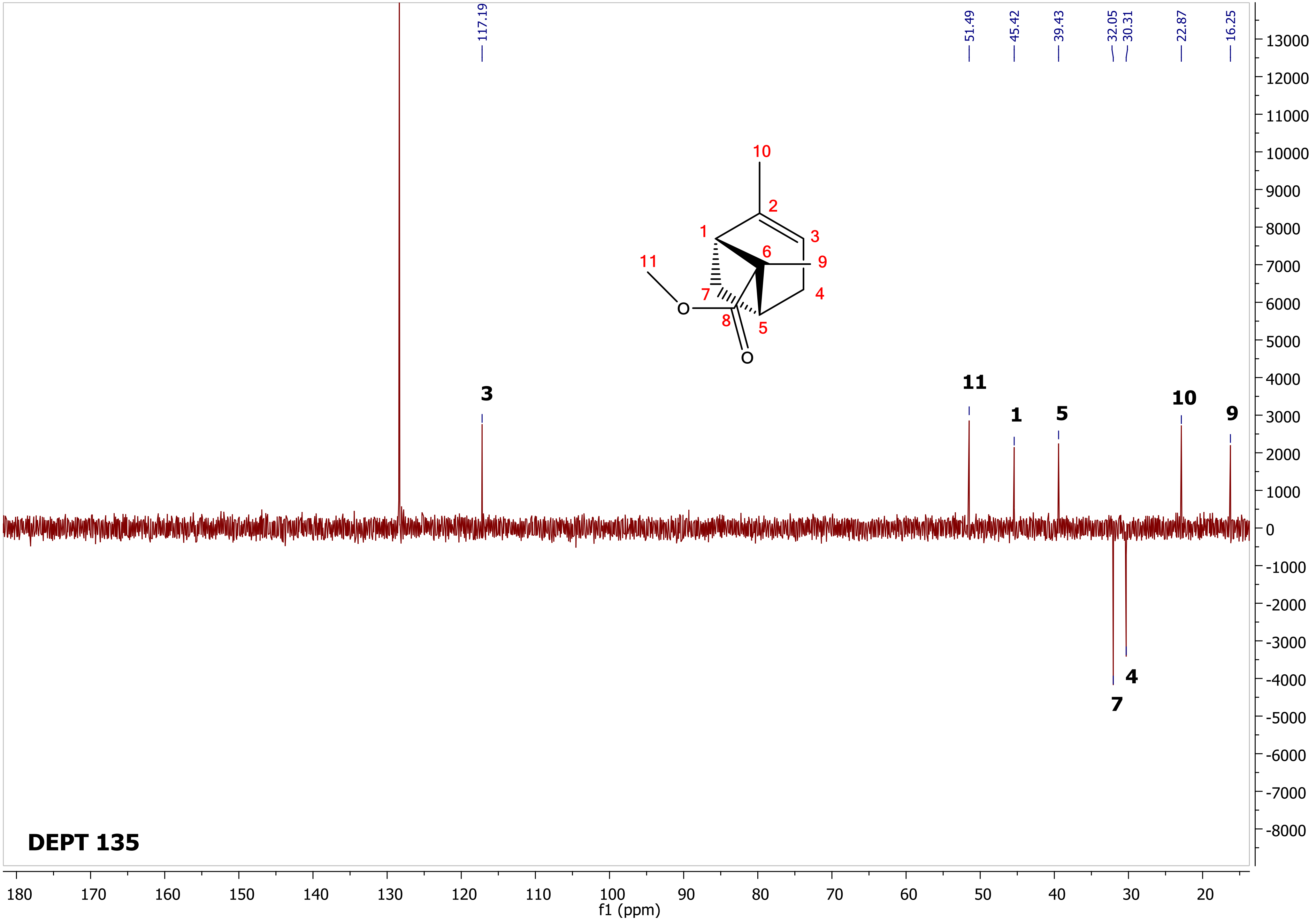

117.19
51.49
45.42
39.43
32.05
30.31
22.87
16.25
10
2
1
3
6
9
11
7
4
O
8
5
O
3
11
1
5
10
9
4
7
DEPT 135
180
170
160
150
140
130
120
110
100
90
80
70
60
50
40
30
20
f1 (ppm)
13000
12000
11000
10000
9000
8000
7000
6000
5000
4000
3000
2000
1000
0
-1000
-2000
-3000
-4000
-5000
-6000
-7000
-8000

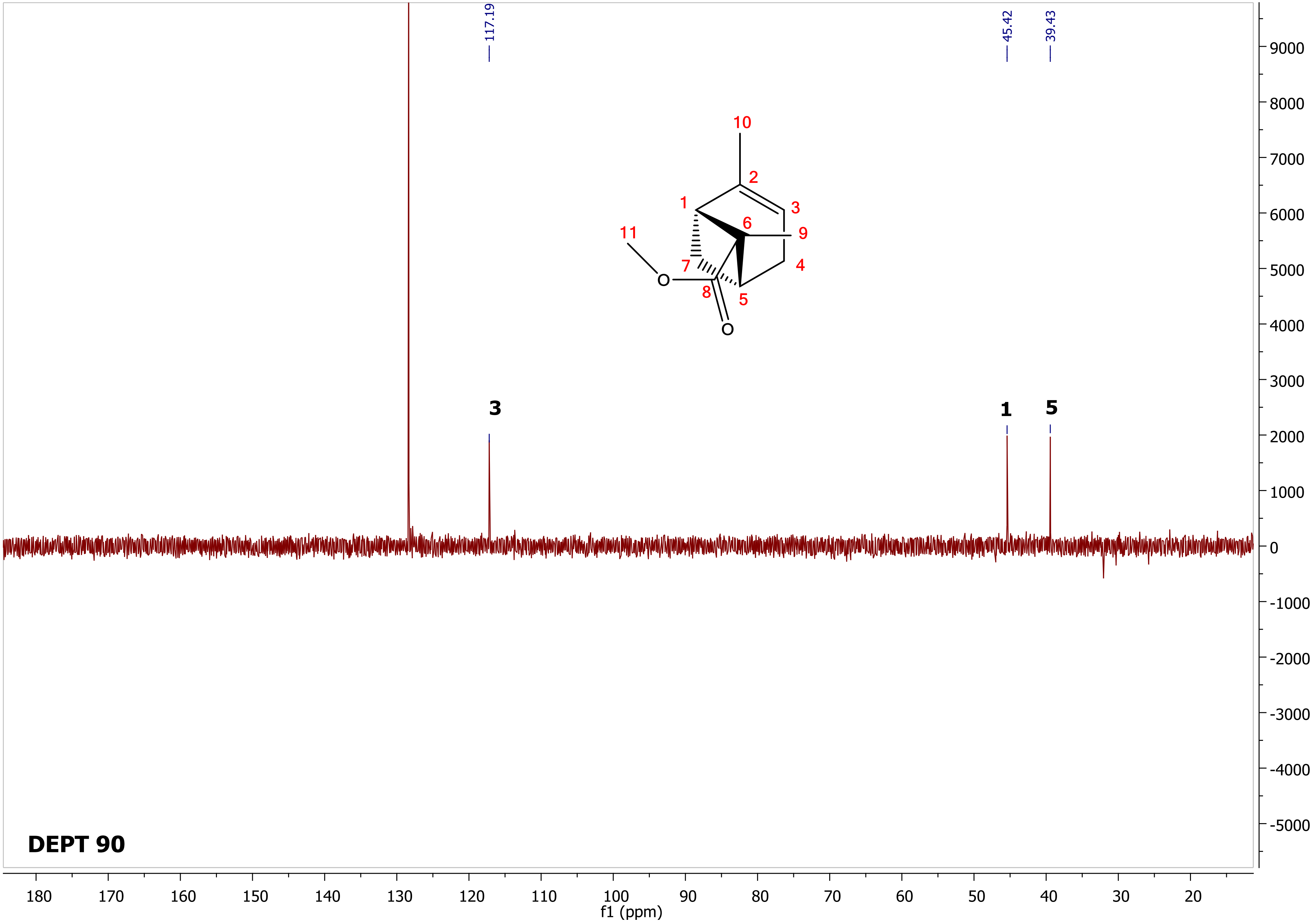
117.19
45.42
39.43
10
2
1
3
6
9
11
7
4
O
8
5
O
3
1
5
DEPT 90
f1 (ppm)

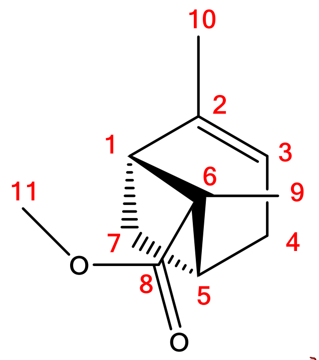
10
2
1
3
6
11
9
7
4
O
8
5
O

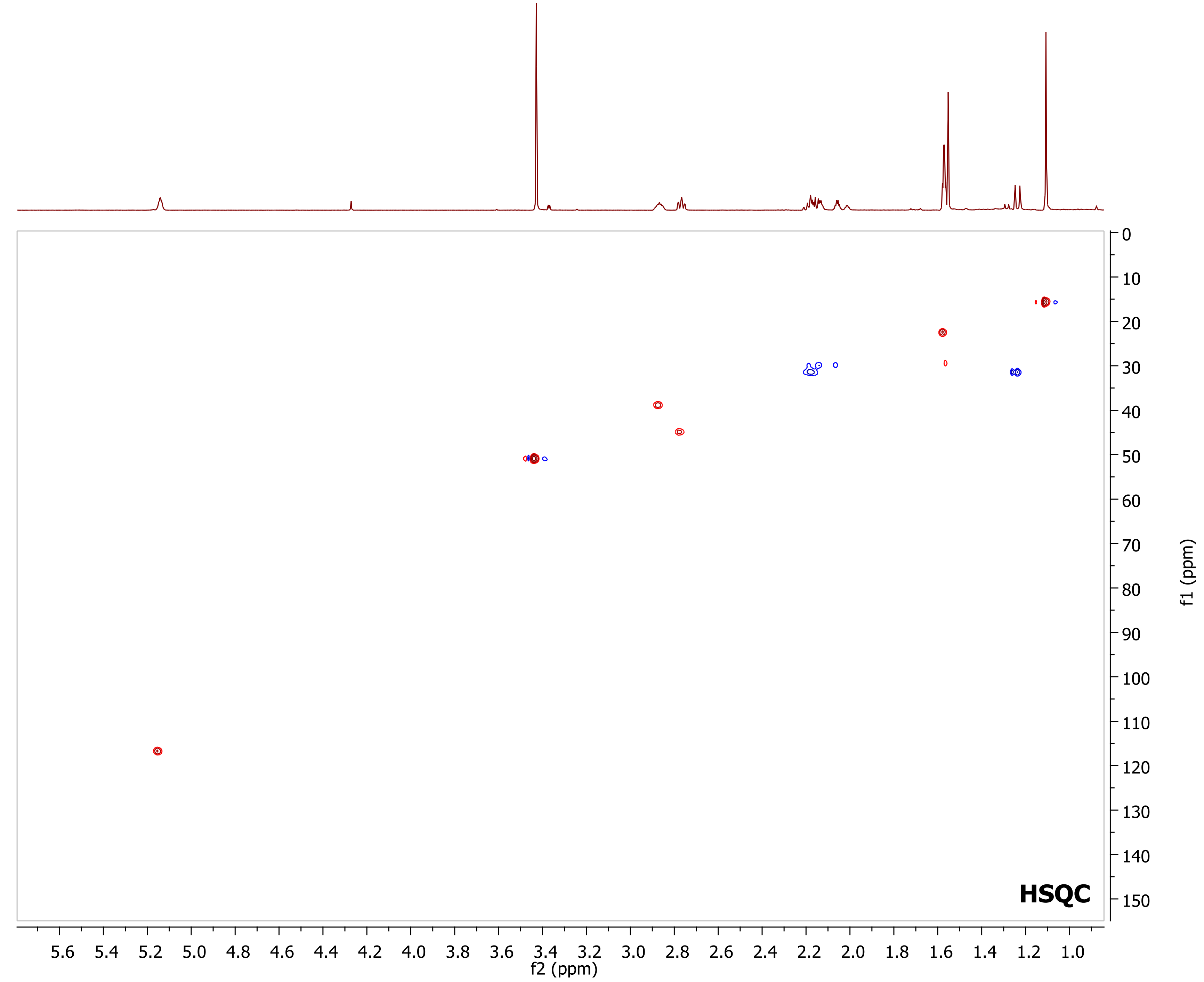
HSQC
f1 (ppm)
f2 (ppm)
5.6
5.4
5.2
5.0
4.8
4.6
4.4
4.2
4.0
3.8
3.6
3.4
3.2
3.0
2.8
2.6
2.4
2.2
2.0
1.8
1.6
1.4
1.2
1.0
0
10
20
30
40
50
60
70
80
90
100
110
120
130
140
150

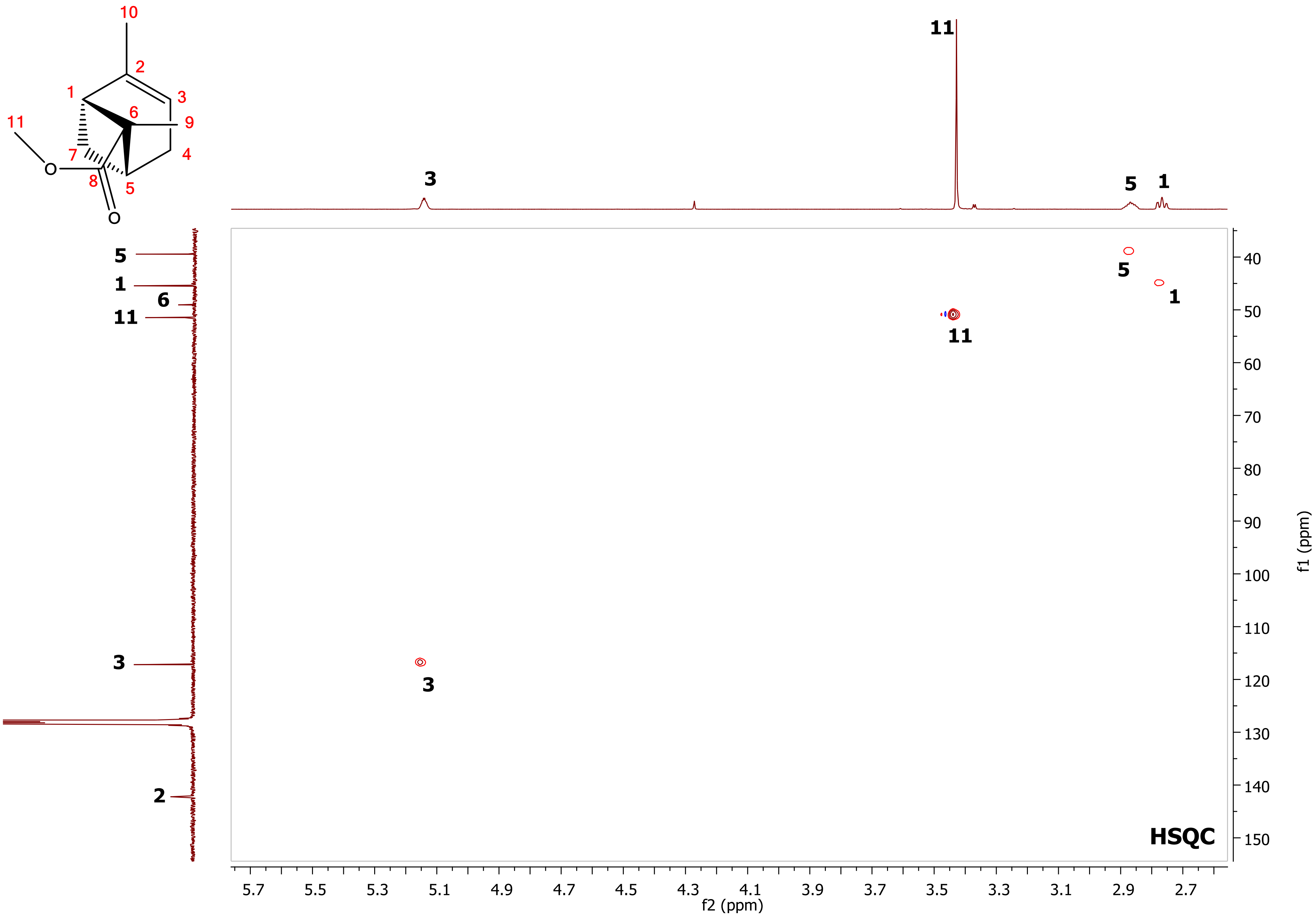

10
2
1
3
11
6
9
7
4
O
8
5
O
3
11
5
1
5
1
6
11
3
2
5
1
11
3
HSQC
40
50
60
70
80
90
100
110
120
130
140
150
f1 (ppm)
5.7
5.5
5.3
5.1
4.9
4.7
4.5
4.3
4.1
3.9
3.7
3.5
3.3
3.1
2.9
2.7
f2 (ppm)

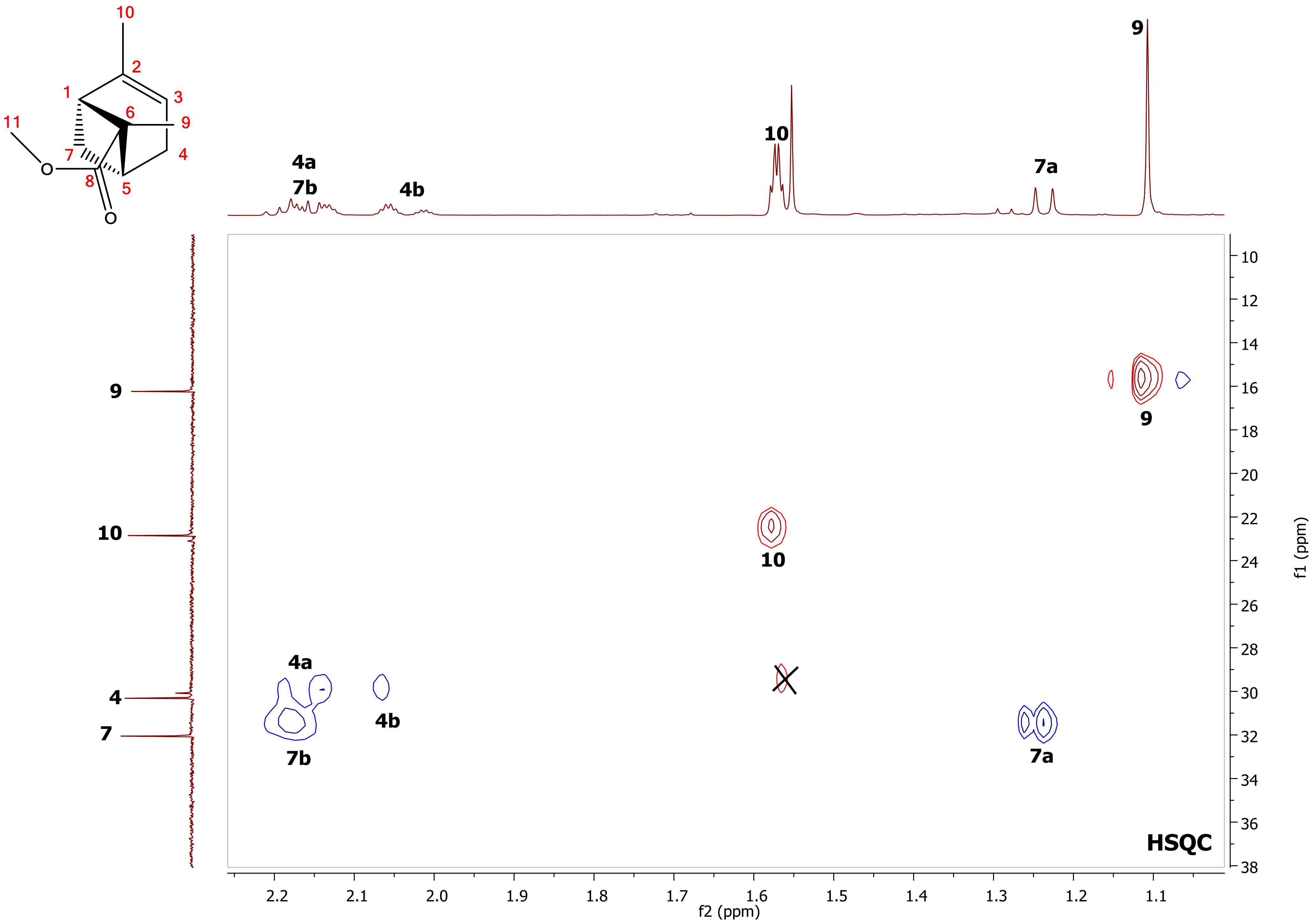

10
2
1
3
6
9
11
7
4
O
8
5
O
4a
7b
4b
10
7a
9
10
4
7
9
10
4a
4b
7b
7a
HSQC
f2 (ppm)
f1 (ppm)

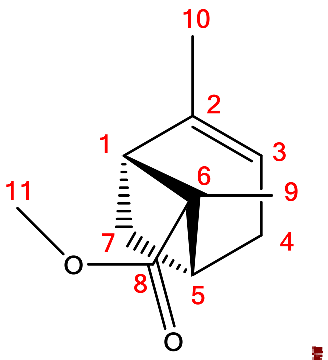
10
2
1
3
6
9
11
7
4
O
8
5
O

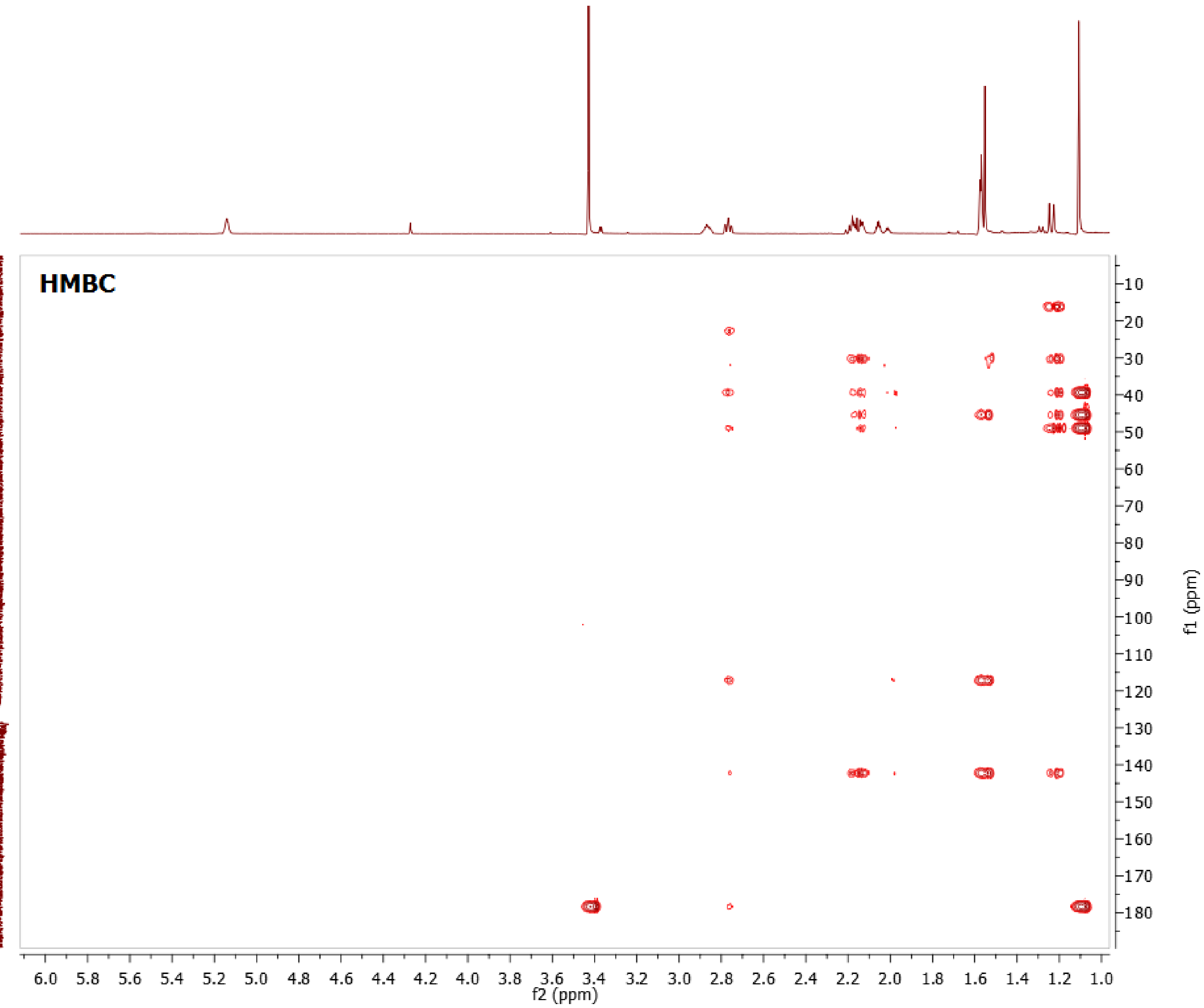
HMBC
6.0
5.8
5.6
5.4
5.2
5.0
4.8
4.6
4.4
4.2
4.0
3.8
3.6
3.4
3.2
3.0
2.8
2.6
2.4
2.2
2.0
1.8
1.6
1.4
1.2
1.0
f2 (ppm)
10
20
30
40
50
60
70
80
90
100
110
120
130
140
150
160
170
180
f1 (ppm)

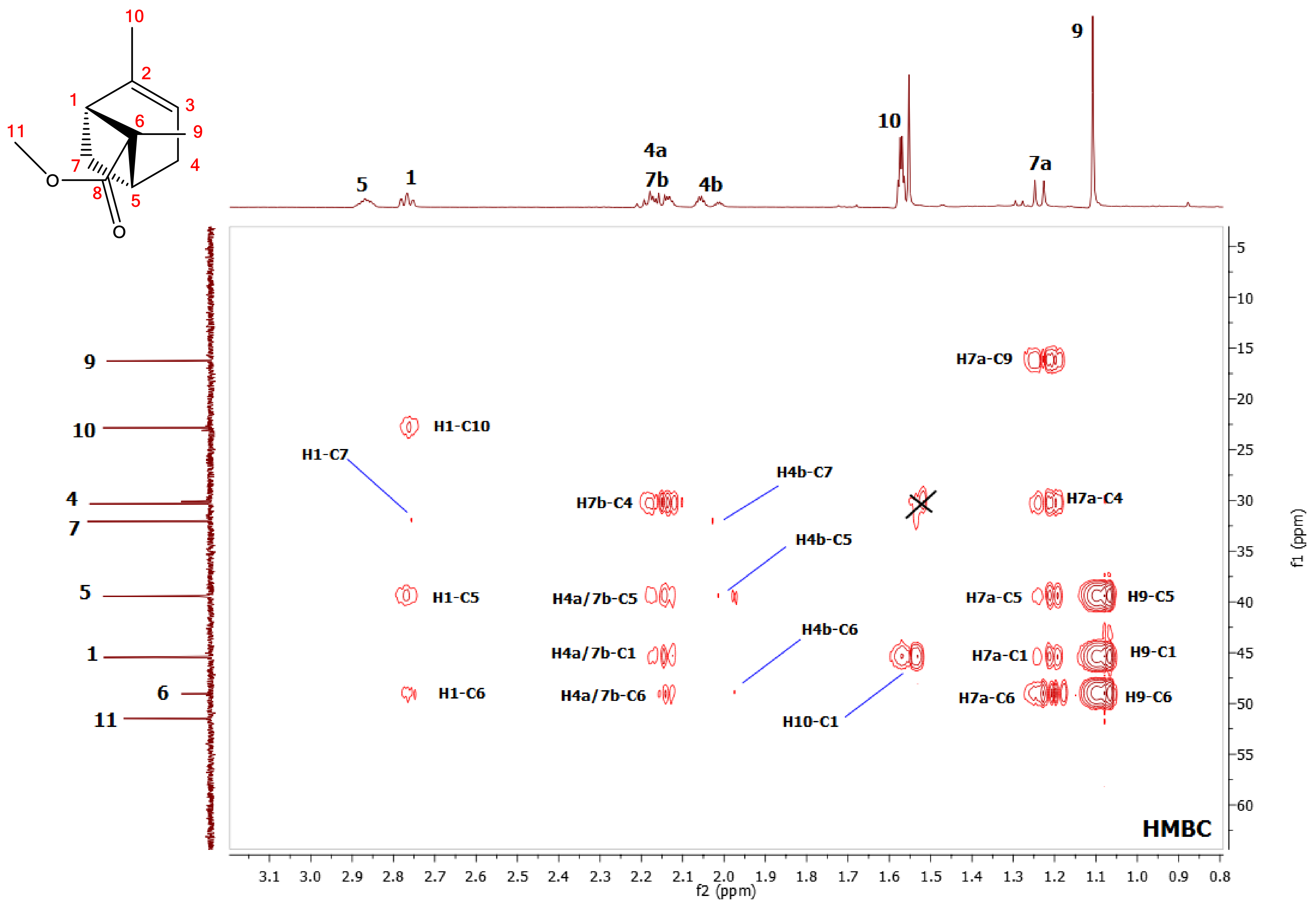

10
2
1
3
6
9
11
7
4
O
8
5
O
9
10
4a
7b
4b
5
1
7a
9
10
4
7
5
1
6
11
H7a-C9
H1-C10
H1-C7
H4b-C7
H7b-C4
H7a-C4
H4b-C5
H1-C5
H4a/7b-C5
H7a-C5
H9-C5
H4b-C6
H4a/7b-C1
H7a-C1
H9-C1
H1-C6
H4a/7b-C6
H7a-C6
H9-C6
H10-C1
HMBC
f2 (ppm)
f1 (ppm)

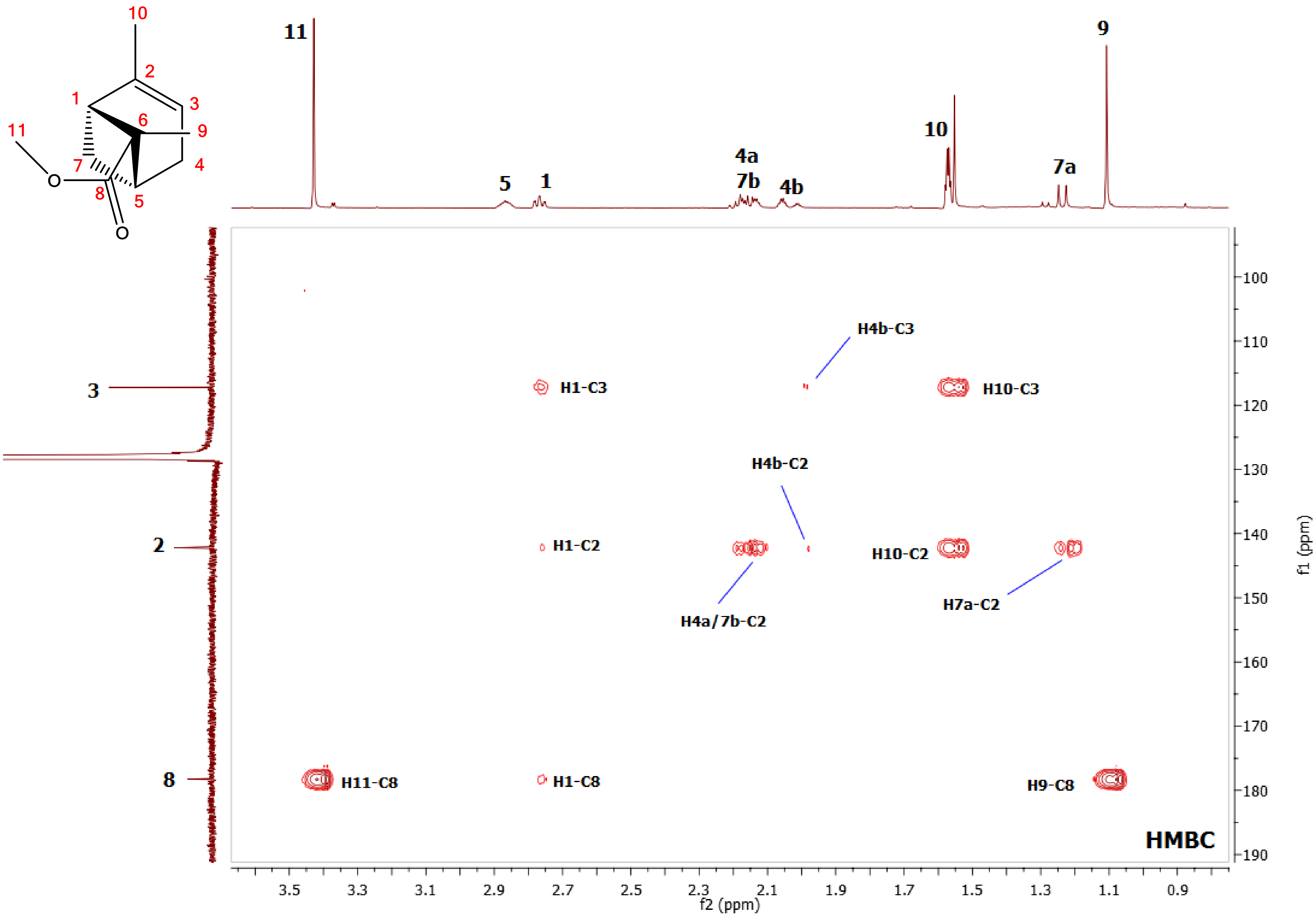

10
2
1
3
6
9
11
7
4
O
8
5
O
11
5
1
4a
7b
4b
10
7a
9
3
2
8
H4b-C3
H1-C3
H10-C3
H4b-C2
H1-C2
H10-C2
H4a/7b-C2
H7a-C2
H11-C8
H1-C8
H9-C8
HMBC
f1 (ppm)
f2 (ppm)

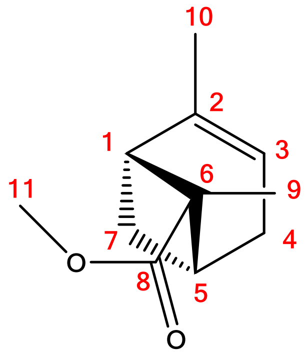
10
2
1
3
11
6
9
7
O
4
8
5
O

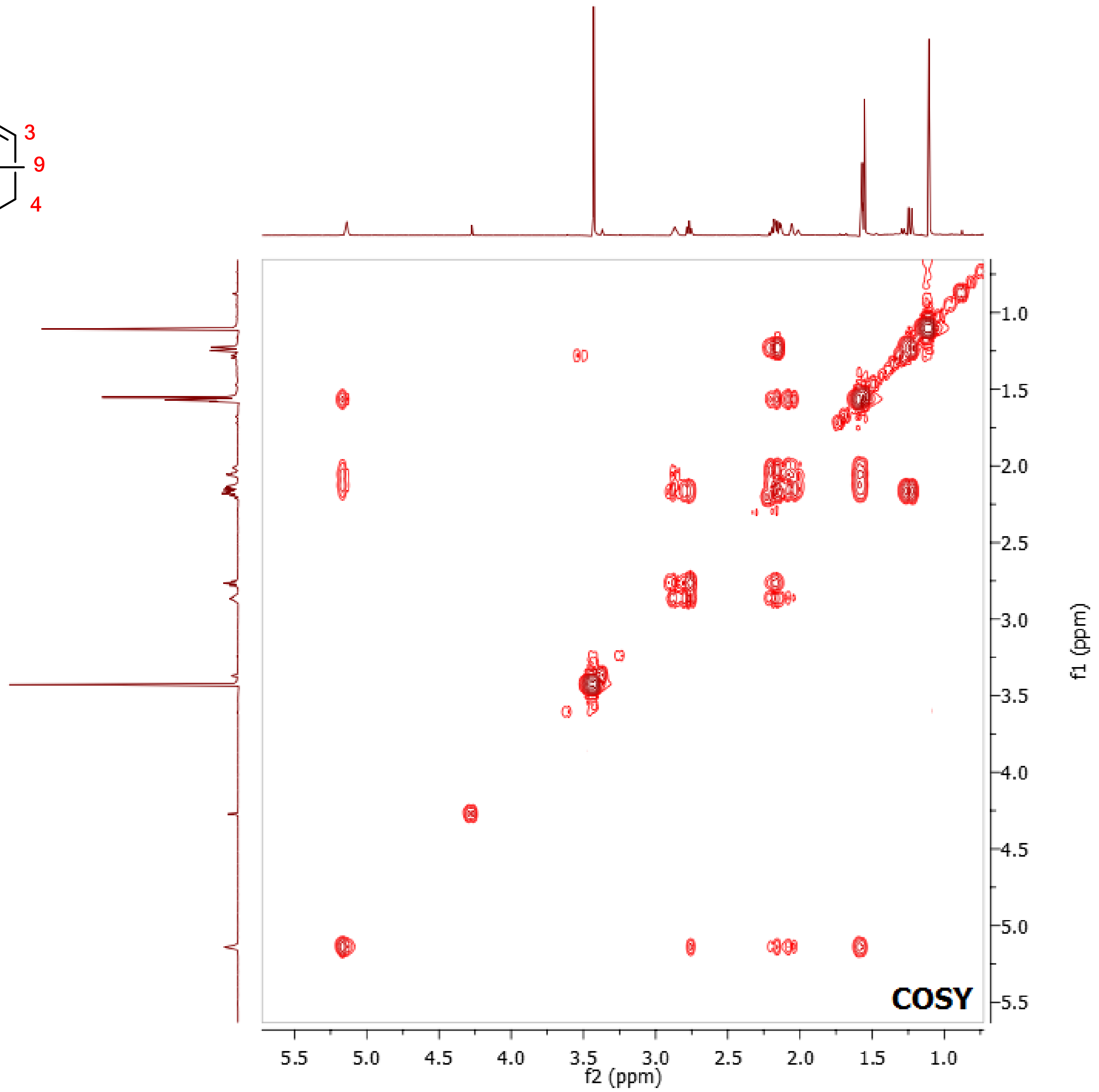
COSY
f2 (ppm)
f1 (ppm)
5.5
5.0
4.5
4.0
3.5
3.0
2.5
2.0
1.5
1.0

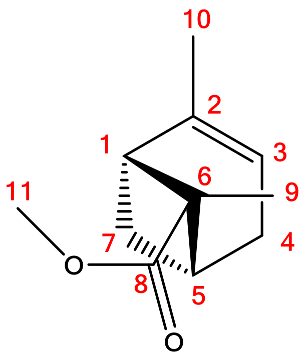
10
2
1
3
6
9
11
7
4
O
8
5
O

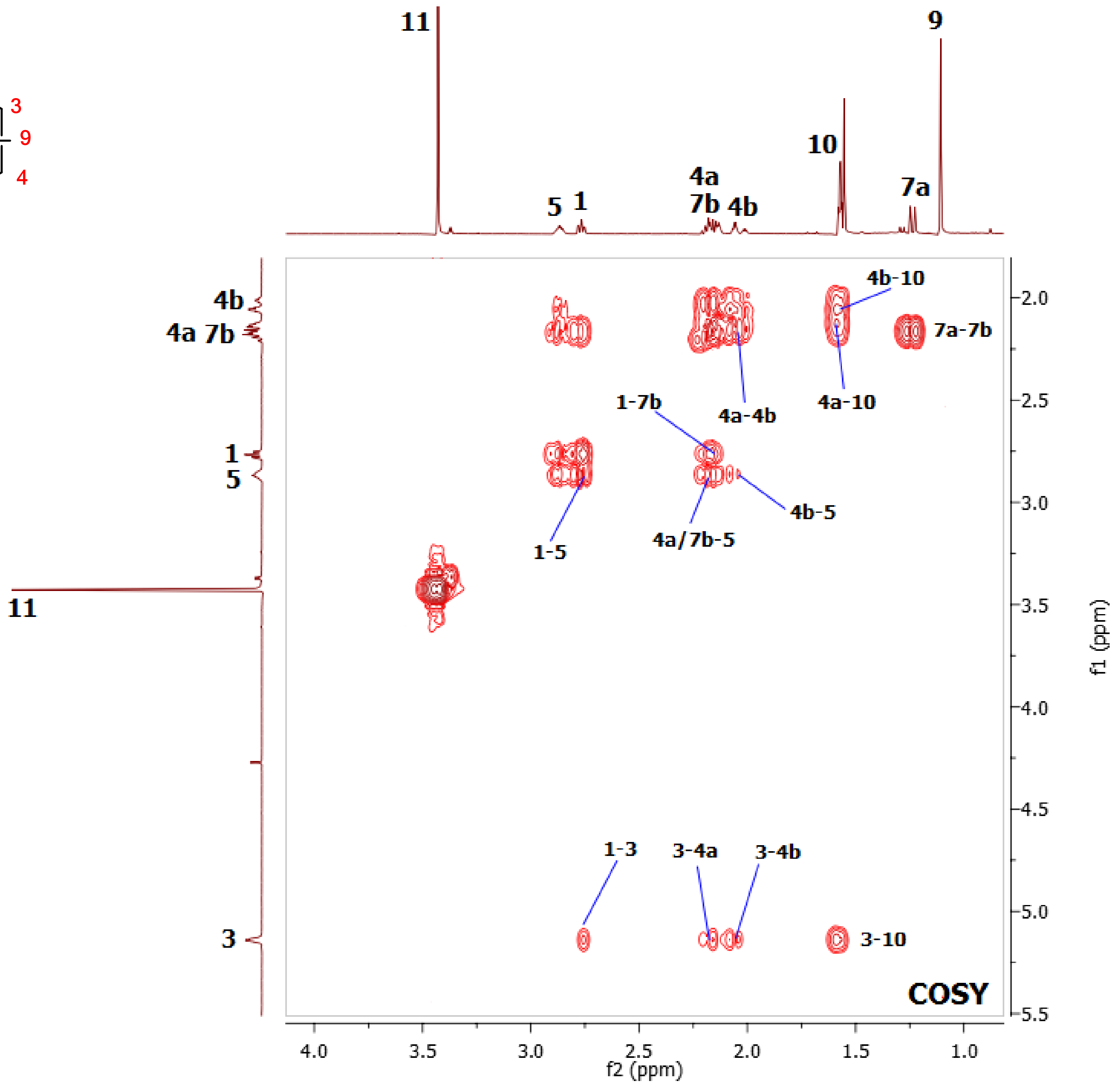
11
5 1
4a
7b 4b
10
7a
9
4b
4a 7b
1
5
11
3
4b-10
7a-7b
1-7b
4a-4b
4a-10
4b-5
4a/7b-5
1-5
1-3
3-4a
3-4b
3-10
COSY
f2 (ppm)
f1 (ppm)
4.0
3.5
3.0
2.5
2.0
1.5
1.0
2.0
2.5
3.0
3.5
4.0
4.5
5.0
5.5

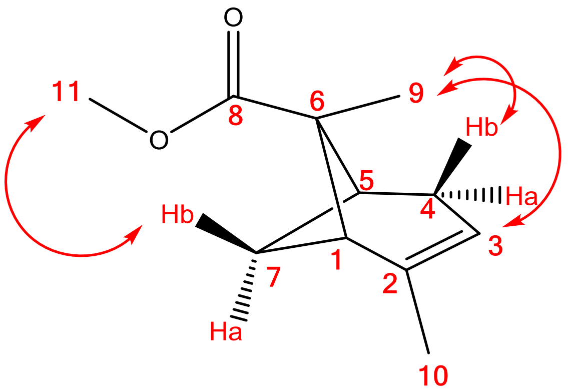

Key NOEs for compound **D**

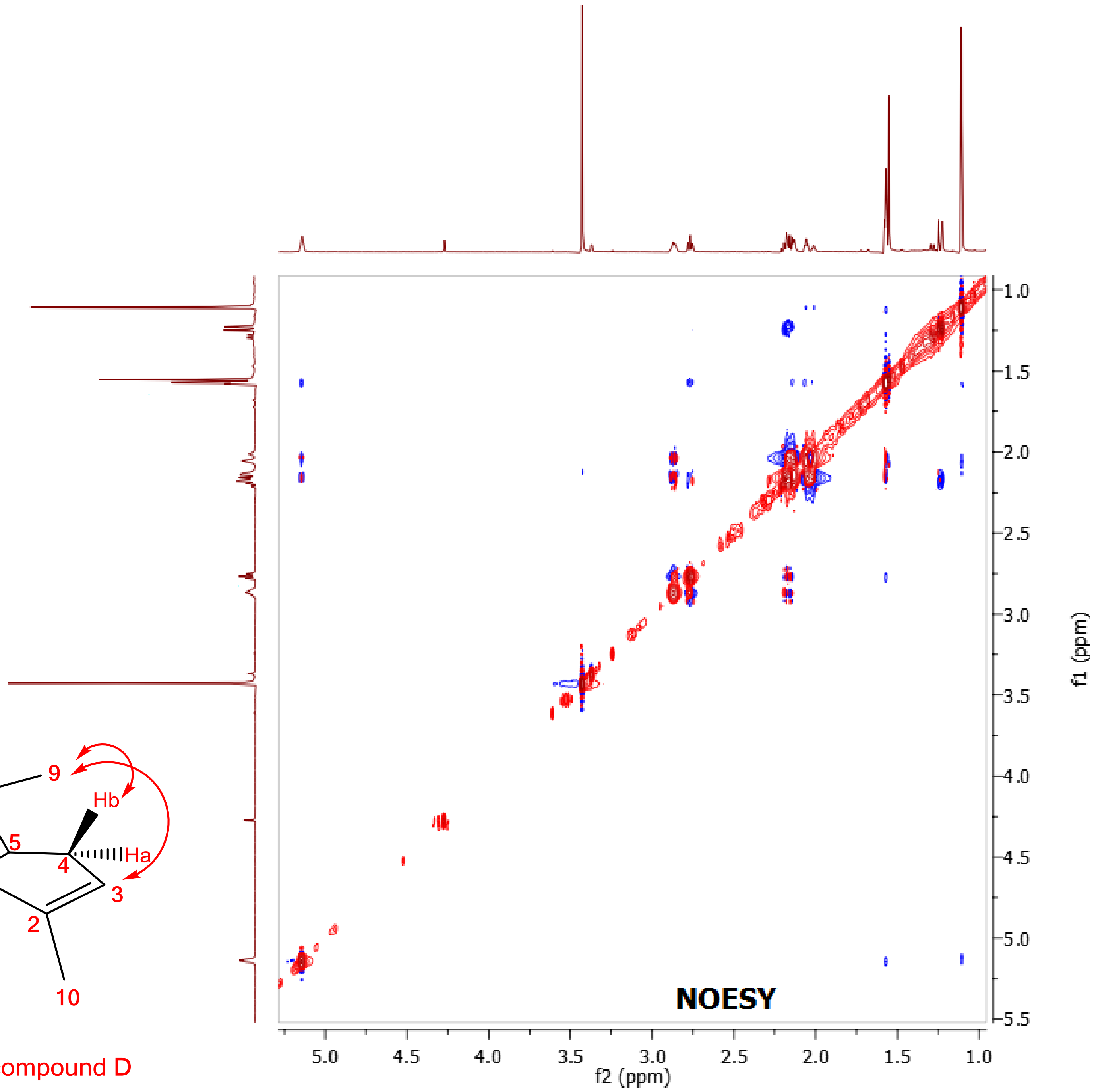

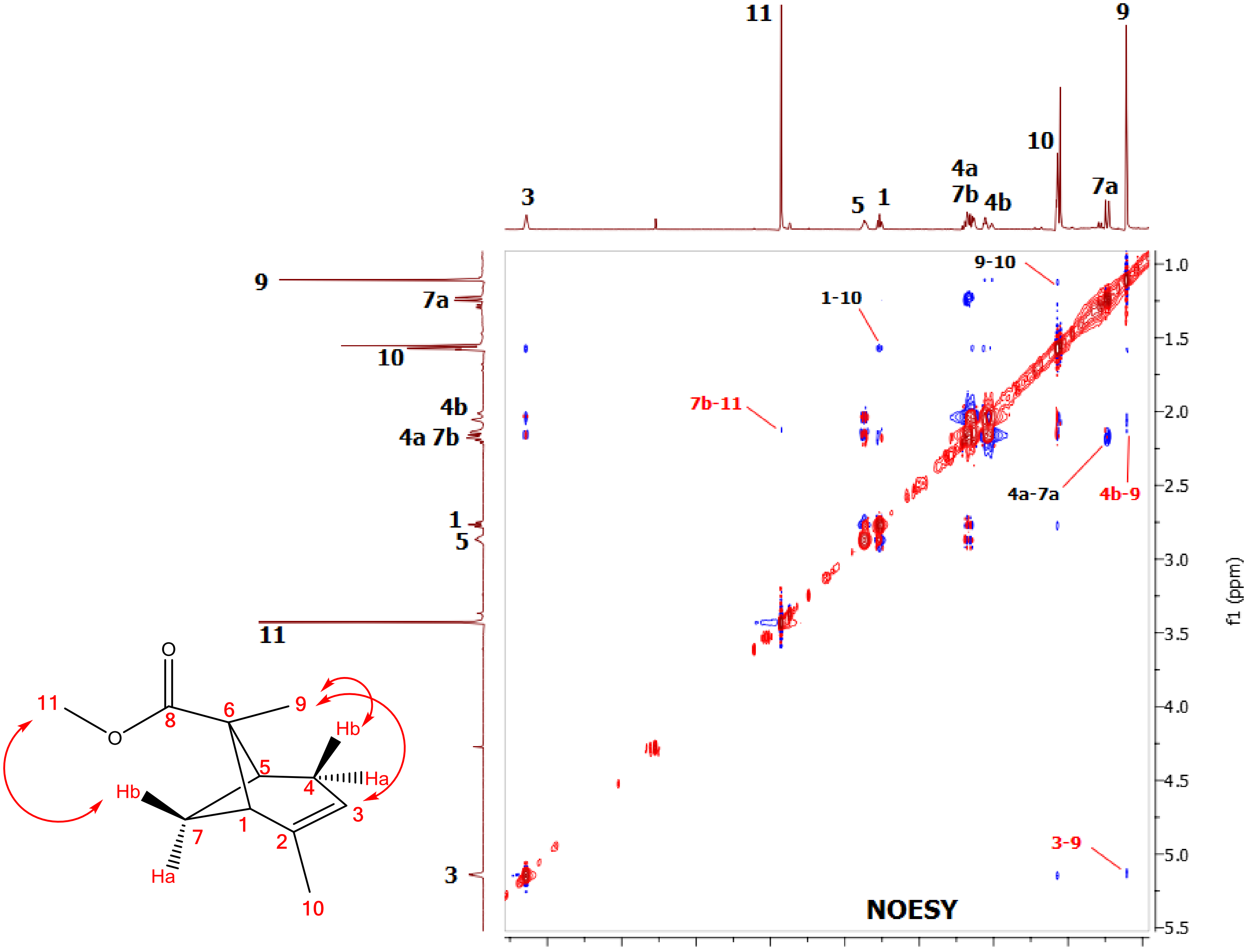

Key NOEs for compound **D**
